# Variable Selection for Fixed and Random Effects in Multilevel Functional Mixed Effects Models

**DOI:** 10.1002/sim.70694

**Published:** 2026-08-09

**Authors:** Rahul Ghosal, Marcos Matabuena, Enakshi Saha

**Affiliations:** ^1^ Department of Epidemiology and Biostatistics University of South Carolina Columbia USA; ^2^ Mohamed bin Zayed University of Artificial Intelligence Abu Dhabi UAE

**Keywords:** accelerometer data, MAP estimation, multilevel functional mixed effects model, NHANES, random effects selection, variable selection

## Abstract

We develop a new method for simultaneously selecting fixed and random effects in a multilevel functional regression model. The proposed method is motivated by accelerometer‐derived physical activity data from the 2011 to 2012 cohort of the National Health and Nutrition Examination Survey (NHANES), with the aim of identifying age and race‐specific heterogeneity in covariate effects on the diurnal pattern of physical activity across the lifespan. Existing methods for variable selection in function‐on‐scalar regression have primarily been designed for fixed effect selection and for single‐level functional data. In high‐dimensional multilevel functional regression, the presence of cluster‐specific heterogeneity in covariate effects could be detected through sparsity in fixed and random effects, and for this purpose, we propose a multilevel functional mixed effects selection (MuFuMES) method. The fixed and random functional effects are modelled using splines, with spike‐and‐slab group lasso (SSGL) priors on the unknown parameters of interest, and a computationally efficient MAP estimation approach is employed for mixed effect selection through an Expectation Conditional Maximization (ECM) algorithm. Numerical analysis using simulation study illustrates the satisfactory selection accuracy of the variable selection method in having a negligible false‐positive and false‐negative rate. The proposed method is applied to the NHANES 2011‐12 accelerometer data, where it effectively identifies age and race‐specific heterogeneity in covariate effects on the diurnal pattern of physical activity, recovering biologically meaningful insights.

## 1 Introduction

Functional regression models [1, 2] are widely used for modelling dynamic effects of scalar and functional covariates on a functional response of interest, varying over some continuous index such as time, space, age, or other similar domain. Functional regression models have diverse applications in various disciplines ranging from agriculture [3, 4], growth curve modelling [5, 6], economics [7], biological sciences [8, 9], imaging [10, 11, 12], physical activity research [13, 14, 15], and many other areas. When each observational unit has a unique functional observation associated with them, the data structure is referred to as single‐level functional data. Multiple methods have been developed over the last two decades for smooth estimation and prediction [16, 17, 18, 19] for such single‐level functional regression models. See [20] and the references therein for existing modelling approaches in single‐level functional data.

With the rapid development of technology in biomedical sciences, multiple functional curves can now be measured for each observational unit of interest, for example, functional observations over multiple longitudinal visits [21], hierarchical structure or nested functional data [22] with multiple replications for each unit of interest, leading to a *multilevel* functional data structure. Multiple regression and modelling approaches [23, 24, 25, 26, 27, 28, 29, 30, 31] have been developed for such “second‐generation” and multilevel functional data, accounting for the within‐cluster correlation.

Functional regression models are increasingly becoming high‐dimensional due to the advancement of sensors and medical devices [32], which can enable the collection of multiple functional observations from different modalities along with a large number of scalar covariates such as demographic, clinical, and genetic information of subjects or units. Several variable selection methods have been developed in single‐level functional regression models using both frequentist [33, 34, 35, 36, 37, 38] and Bayesian approaches [14, 39, 40, 41], which are aimed at estimating the dynamic association of the influential predictors on the functional response of interest. These methods enable the selection of the functional fixed effects, thus enhancing interpretability. While multiple regression frameworks have been developed for functional mixed effects models [42] and multilevel functional regression models [24, 25, 27, 43, 44], high dimensionality in such hierarchical or clustered functional data can pose significant computation challenges.

### 1.1 Motivating Application

**FIGURE 1:**
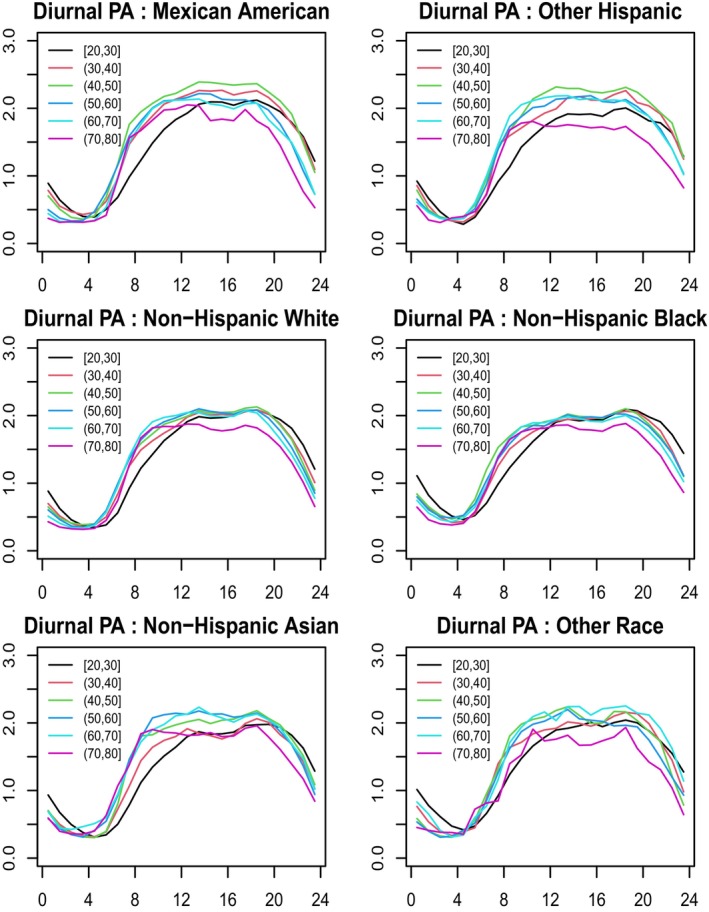
The average diurnal patterns of PA (log‐transformed) for the six age and ethnicity groups in NHANES 2011‐12.

Our motivating application comes from the accelerometer‐derived physical activity data in the 2011‐2012 cohort of the National Health and Nutrition Examination Survey (NHANES). We are interested in identifying and understanding whether there exists any age and race‐specific heterogeneity in the effects of key demographic (e.g., gender), clinical (e.g., BMI), lifestyle (e.g., diet), and socioeconomic covariates (e.g., income) on the diurnal patterns of physical activity (PA) across the lifespan. Previous research in the NHANES 2003–2006 cohorts has demonstrated considerable heterogeneity in the effect of aging and race/ethnicity on summary‐level PA metrics and diurnal PA patterns [27, 45, 46, 47, 48]. However, the key drivers of the diurnal PA patterns, and the age and race‐specific heterogeneity in their effects have been less explored. NHANES 2011‐2012 reports individuals' acceleration in Monitor Independent Movement Summary (MIMS) unit [49]. Figure 1 displays the observed diurnal pattern in PA among adults across six age groups (20−30,30−40,…,70−80) and six races (ethnicities). It can be observed that there exists considerable heterogeneity in the diurnal PA patterns among the age‐groups and across races. Our objective is to develop a method that can simultaneously (i) identify key drivers of diurnal patterns in PA, (ii) detect covariates with age or race‐specific heterogeneity in their effects, and (iii) estimate their dynamic associations with PA.

### 1.2 Contributions

The existing methods for variable selection in single‐level functional data pointed above cannot handle such clustered functional data or achieve joint selection of fixed and random functional effects. Understanding the cluster‐specific heterogeneity in covariate effects on the functional outcome requires accurate identification of the fixed and random functional effects in a multilevel functional mixed effects model. However, due to its computational complexity, joint variable selection methods for fixed and random functional effects have been less explored for the multilevel functional mixed effect model. While several methods have been developed for variable selection for fixed and random effects in classical linear mixed‐effects models [50, 51, 52, 53, 54, 55] using frequentist and Bayesian approaches, these methods cannot impose group‐level sparsity, a fundamental requirement for nonparametric and functional regression. To fill this research gap, in this article, we propose a new method for simultaneously selecting fixed and random effects in a multilevel functional regression model. We model the fixed and random smooth functional effects using splines and impose spike‐and‐slab group lasso (SSGL) priors on both the fixed effect basis coefficients [41, 56] and random effect covariance parameters, represented by a Cholesky decomposition of the covariance matrix [51, 52]. We develop a computationally efficient approach for mixed effect selection through an expectation conditional maximization (ECM) algorithm which derives maximum a posteriori (MAP) estimates [41, 57, 58]. The SSGL prior has been shown to perform adaptive shrinkage [41, 56] compared to other group‐based penalties. The resulting MAP estimator enjoys exact sparsity for the fixed and random functional effects and under a non‐separable beta‐Bernoulli prior on the mixing proportions and enables sharing of information across the fixed and random functional effects. The proposed method provides a unified framework for joint selection of important fixed and random functional effects, while also simultaneously estimating these dynamic effects. To the best of our knowledge, this is the first work proposing fixed and random effect selection in a multilevel functional regression model and therefore also providing a first group selection method of fixed and random effects in high‐dimensional linear mixed effects models.

The key methodological contributions of this article include (i) A joint variable selection approach of fixed and random functional effects in multilevel functional mixed models using a semiparametric spline‐based formulation. (ii) Employing a computationally efficient MAP estimation strategy using an Expectation Conditional Maximization (ECM) algorithm under a spike‐and‐slab group lasso (SSGL) prior on the fixed effect coefficients and random effect covariance parameters of interest corresponding to a Cholesky decomposition of the random effect covariance matrix. (iii) An automated data‐driven procedure for tuning parameter selection for fixed and random effects, which are allowed to be different for these two components, thus accounting for the scale difference in the two kinds of parameters. The proposed method also directly provides a way of doing group‐level selection of fixed and random effects in linear mixed effects models. Note that the proposed method for the multilevel functional mixed effects selection (MuFuMES) is flexible and can accommodate both scalar or functional covariates, which may or may not vary within the repeated observations of the clusters.

Numerical analysis using simulations illustrates the satisfactory selection and estimation accuracy of the proposed method. Finally, the proposed MuFuMES method is applied to accelerometer data from the 2011 to 2012 cohort of the National Health and Nutrition Examination Survey (NHANES) to identify and understand the age and race‐specific heterogeneity in covariate effects on the diurnal patterns of physical activity across the lifespan. The rest of this article is organized as follows. We introduce the modeling framework and the multilevel functional mixed effect model in Section 2 and illustrate the proposed variable selection method MuFuMES. The finite sample performance of the proposed method is investigated via simulations in Section 3. Real data application of the proposed MuFuMES method is demonstrated on the NHANES 2011‐12 accelerometer data in Section 4. We conclude with a discussion on the strengths and limitations of the proposed method and some possible extensions of this work in Section 5.

## 2 Methodology

### 2.1 Modeling Framework

We denote the functional response for the jth replication within the ith cluster as $Y_{ij}(s),s\in 𝒮$ (i=1,…,n, $j=1,\ldots ,J_{i}$), where 𝒮 is a compact domain. In practice, the functional response is often observed on a finite set of points. In this article, we assume the functions are observed on a dense and regular grid ${𝒯}_{m}=\{s_{1},s_{2},\ldots ,s_{m}\}\subset 𝒮=[0,1]$, without loss of generality, although this can be relaxed and easily extended to accommodate more general scenarios, for example, sparse and irregular domain. For example, in our motivating application, $Y_{ij}(s)$ denotes the diurnal PA pattern of the jth participant within the ith cluster (age‐by‐race cluster) at time‐of‐day 𝒮. The fixed effect predictors of interest are denoted by $X_{ij}={\left(X_{ij1},\ldots ,X_{ijp}\right)}^{T}\in {\mathbb{R}}^{p}$ (e.g., BMI, Gender, Income, etc.) and the random effect predictors of interest are denoted by $Z_{ij}={\left(Z_{ij1},\ldots ,Z_{ijq}\right)}^{T}\in {\mathbb{R}}^{q}$. Often we set $Z_{ij}=X_{ij}$ but this is not necessary [27, 51]. We allow for both fixed and random intercepts, by absorbing them in the $X_{ij1},Z_{ij1}$, respectively. We posit the following multilevel functional mixed effects model,

(1) $$Y_{ij}(s)=\overset{p}{\underset{k=1}{\sum }}X_{ijk}\beta_{k}(s)+\overset{q}{\underset{r=1}{\sum }}Z_{ijr}u_{ir}(s)+v_{ij}(s)+\epsilon_{ij}(s).$$

The functional fixed effects (including intercepts) are denoted by $\beta_{k}(\cdot ),k=1,\ldots ,p$. The functional random effects (including intercepts) are denoted by $u_{ir}(\cdot ),r=1,\ldots ,q$, which are assumed to be smooth [35, 37] and zero mean stochastic processes. Furthermore, we assume strict exogeneity, that is, $u_{ir}(\cdot )$ is independent of the covariate vectors $X_{ij}$ and $Z_{ij}$ for all i,j. Under these and standard regularity conditions (full rank design matrices), the fixed and random functional effects are identifiable, since $E(Y_{ij}(s)|X_{ij},Z_{ij})=\sum_{k=1}^{p}X_{ijk}\beta_{k}(s)$ under these assumptions, ensuring the random effects do not systematically absorb part of the fixed effects. Here, $v_{ij}(s)$ denotes the cluster‐subject specific deviation (for subject j within cluster i) which is assumed to be a zero mean stochastic process with unknown covariance structure and independent of cluster‐specific effects $u_{ir}(s)$. The measurement error $\epsilon_{ij}(s)$ are assumed to be i.i.d, independent of $u_{ir}(s),v_{ij}(s)$ and distributed as $N(0,\sigma^{2})$. The within‐curve correlations are introduced through the functional random effects $u_{ir}(\cdot )$ and $v_{ij}(\cdot )$. Note that, for each fixed s∈𝒮, the model (1) corresponds to the classical linear mixed effects model [51, 52]. Hence, the proposed model serves as a generalization of the linear mixed effects model in functional domains. In this article, our primary objective is to simultaneously select the important fixed and random predictors among $X_{ij},Z_{ij}$ and to also estimate their smooth effects. We illustrate the multilevel functional mixed effects selection (MuFuMES) approach in the following section.

### 2.2 Multilevel Functional Mixed Effects Selection

We model the fixed functional effects using cubic B‐spline basis expansions as $\beta_{k}(s)=\sum_{u=1}^{d}\gamma_{ku}B_{ku}(s)$ (k=1,…,p), here $\gamma_{k}={\left(\gamma_{k1},\ldots ,\gamma_{kd}\right)}^{T}$ are unknown fixed basis coefficients. Similarly, we model the cluster‐level functional random effects using cubic B‐spline basis expansions as $u_{ir}(s)=\sum_{v=1}^{d^{\prime }}\eta_{irv}B_{rv}(s)$ (r=1,…,q), where $\eta_{ir}={\left(\eta_{ir1},\ldots ,\eta_{ird^{\prime }}\right)}^{T}$ are random coefficients. Finally, the cluster‐subject level functional random error $v_{ij}(s)$ is also modelled using a cubic B‐spline basis expansion as $v_{ij}(s)=\sum_{l=1}^{L}\zeta_{ijl}B_{l}(s)$. In this article, we have used B‐spline basis functions for both these effects; however, in general, any basis functions can be used. Plugging in these basis expansions in model (1), we get

(2) $$\begin{array}{ll} Y_{ij}(s) & =\overset{p}{\underset{k=1}{\sum }}X_{ijk}\overset{d}{\underset{u=1}{\sum }}\gamma_{ku}B_{ku}(s)+\overset{q}{\underset{r=1}{\sum }}Z_{ijr}\overset{d^{\prime }}{\underset{v=1}{\sum }}\eta_{irv}B_{rv}(s)\\ & \;+\overset{L}{\underset{l=1}{\sum }}\zeta_{ijl}B_{l}(s)+\epsilon_{ij}(s)\\ & =\overset{p}{\underset{k=1}{\sum }}\overset{d}{\underset{u=1}{\sum }}\gamma_{ku}X_{ijk}B_{ku}(s)+\overset{q}{\underset{r=1}{\sum }}\overset{d^{\prime }}{\underset{v=1}{\sum }}\eta_{irv}Z_{ijr}B_{rv}(s)\\ & \;+\overset{L}{\underset{l=1}{\sum }}\zeta_{ijl}B_{l}(s)+\epsilon_{ij}(s)\\ & ={\tilde{X}}_{ij}{\left(s\right)}^{T}\gamma +{\tilde{Z}}_{ij}{\left(s\right)}^{T}\eta_{i}+{\tilde{W}}_{ij}{\left(s\right)}^{T}\zeta_{ij}+\epsilon_{ij}(s). \end{array}$$

Here ${\tilde{X}}_{ij}{\left(s\right)}^{T}$ denotes the stacked row vector of ${\left\{X_{ijk}B_{ku}(s)\right\}}_{k=1,u=1}^{p,d}$ with length dp, ${\tilde{Z}}_{ij}{\left(s\right)}^{T}$ denotes the stacked row vector of ${\left\{Z_{ijr}B_{rv}(s)\right\}}_{r=1,v=1}^{q,d^{\prime }}$ with length $d^{\prime }q$ and ${\tilde{W}}_{ij}{\left(s\right)}^{T}=(B_{1}(s),B_{2}(s),\ldots ,B_{L}(s))$. Similarly $\gamma ={\left(\gamma_{11},\gamma_{12},\ldots ,\gamma_{pd}\right)}^{T}$ is the stacked fixed effect basis coefficients, $\eta_{i}={\left(\eta_{i11},\eta_{i12},\ldots ,\eta_{iqd^{\prime }}\right)}^{T}$ is the stacked random effect basis coefficients for cluster i, and $\zeta_{ij}={\left(\zeta_{ij1},\zeta_{ij2},\ldots ,\zeta_{ijL}\right)}^{T}$. Now stacking the terms $Y_{ij}(s),\epsilon_{ij}(s)$, and ${\tilde{X}}_{ij}{\left(s\right)}^{T},{\tilde{Z}}_{ij}{\left(s\right)}^{T}$, ${\tilde{W}}_{ij}{\left(s\right)}^{T}$ first across the observed functional domain $𝒮=\{s_{1},s_{2},\ldots ,s_{m}\}$ and then across replications $j=1,\ldots ,J_{i}$ we can reformulate (2) as,

(3) $$\begin{array}{l} Y_{i}={𝕏}_{i}\gamma +{\mathbb{Z}}_{i}\eta_{i}+{𝕎}_{i}\zeta_{i}+\epsilon_{i}. \end{array}$$

Here 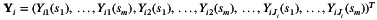 is the stacked response vector and $\epsilon_{i}$ is defined analogously. ${𝕏}_{i}={\left({\tilde{X}}_{i1}(s_{1}),\ldots ,{\tilde{X}}_{i1}(s_{m}),\ldots ,{\tilde{X}}_{iJ_{i}}(s_{1}),\ldots ,{\tilde{X}}_{iJ_{i}}(s_{m})\right)}^{T}$ is the stacked known basis matrix of dimension $mJ_{i}\times dp$. We denote by the stacked matrix ${\mathbb{Z}}_{i}={\left({\tilde{Z}}_{i1}(s_{1}),\ldots ,{\tilde{Z}}_{i1}(s_{m}),\ldots ,{\tilde{Z}}_{iJ_{i}}(s_{1}),\ldots ,{\tilde{Z}}_{iJ_{i}}(s_{m})\right)}^{T}$, a known basis matrix of dimension $mJ_{i}\times d^{\prime }q$ and similarly define ${𝕎}_{i}$. We assume in model (3), $\eta_{i}∼N(0,𝔻)$, $\zeta_{i}∼N(0,\Omega )$ and stacked error vector $\epsilon_{i}∼N(0,\sigma^{2}𝕀)$, where 𝕀 is the identity matrix of dimension $mJ_{i}\times mJ_{i}$, and they are independent and identically distributed across i. Note that this ensures that the random effects $u_{ir}(\cdot ),r=1,\ldots ,q$ are zero mean.

For the purpose of selecting the important fixed and random functional predictors, it is crucial to impose sparsity in the fixed effect coefficients γ and eliminate appropriate variance components in 𝔻 [50, 51, 52] of the cluster‐level random effects. For this purpose, we use a Cholesky decomposition technique [52] of the covariance matrix $𝔻=𝕃{𝕃}^{T}$, where we enforce diag(𝕃)>0 to guarantee uniqueness of the decomposition and ensure 𝕃 is identifiable. Such Cholesky decomposition or modified Cholesky decompositions have been used in the linear mixed models for enforcing sparsity in the cluster‐level random effect components [50, 51, 52]. Sparsity can be enforced in the fixed functional effects by enforcing group sparsity in the fixed effect coefficients γ [36, 41]. This ensures the functional effect is zero only if all the basis coefficients corresponding to the coefficient function are zero, thus giving rise to a natural group selection problem. For enforcing sparsity in the random functional effects, we will illustrate how a group‐based shrinkage on the rows of 𝕃 can be used. Writing the random coefficient $\eta_{i}=𝕃b_{i}$, where $b_{i}∼N(0,{𝕀}_{d^{\prime }q})$, we reformulate model (3) as,

(4) $$\begin{array}{l} Y_{i}={𝕏}_{i}\gamma +{\mathbb{Z}}_{i}𝕃b_{i}+{𝕎}_{i}\zeta_{i}+\epsilon_{i}. \end{array}$$

We also assume that 𝔻 is positive semidefinite, which allows certain components of $\eta_{i}=𝕃bi$ to be zero, with probability 1. Next, we endow the unknown parameters γ and 𝕃, Ω with appropriate priors and illustrate a MAP estimation strategy that maximizes the posterior distribution using an Expectation Conditional Maximization (ECM) algorithm [41, 58].

### 2.3 Prior Specification for Fixed and Random Effect Parameters

We use a spike‐and‐slab group lasso (SSGL) prior [41, 56] on the vector of basis coefficients $\gamma ={\left(\gamma_{1}^{T},\ldots ,\gamma_{p}^{T}\right)}^{T}$ to enforce fixed effect selection, where the grouping corresponds to each functional fixed effect $\beta_{k}(\cdot )$. The SSGL prior is given by,

(5) $$\begin{array}{l} \pi (\gamma |\theta )=\overset{p}{\underset{k=1}{\prod }}[(1-\theta )\Psi (\gamma_{k}|\lambda_{0})+\theta \Psi (\gamma_{k}|\lambda_{1})]. \end{array}$$

Here, θ∈(0,1) denotes a mixing proportion, capturing the expected proportion of nonzero $\gamma_{k}$' coefficient vectors. We denote by Ψ(·|λ) a multivariate Laplace density with hyperparameter λ,

(6) $$\begin{array}{l} \Psi (\gamma_{k}|\lambda )=\frac{\lambda^{d}e^{-\lambda ||\gamma_{k}|{|}_{2}}}{2^{d}\pi^{(d-1)/2}\Gamma ((d+1)/2)},\;k=1,\ldots ,p. \end{array}$$

Under the above SSGL prior denoted as $SSGL(\lambda_{0},\lambda_{1},\theta )$, the posterior mode for γ becomes exactly sparse [56] and hence can used for simultaneous estimation and variable selection. Now, to enforce sparsity in the random effects, we use a group‐based shrinkage on the rows of 𝕃 in (4). In particular, let us denote a partition of the $d^{\prime }q\times d^{\prime }q$ lower triangular matrix 𝕃 as $𝕃={\left[{𝕃}_{1}^{T},\ldots ,{𝕃}_{q}^{T}\right]}^{T}$, where each ${𝕃}_{r}$ (r=1,…q) has $d^{\prime }$ rows and $d^{\prime }q$ columns. Now if an entire block ${𝕃}_{r}$ is zero, the variance‐covariance components in 𝔻 corresponding to the random effects $\eta_{ir}=(\eta_{ir1},\ldots ,\eta_{ird^{\prime }})$ and it's covariance with $\eta_{is}$ (s≠r) is zero, which ensures that the functional random effects $u_{ir}(\cdot )$ is zero for all i. We further denote the stacked (by row) nonzero elements of the matrix ${𝕃}_{r}$ as ${\tilde{L}}_{r}$ and define $\tilde{L}={\left({\tilde{L}}_{1}^{T},\ldots ,{\tilde{L}}_{q}^{T}\right)}^{T}$. We endow on $\tilde{L}$ a SSGL prior $SSGL(\nu_{0},\nu_{1},\theta^{*})$, given by

(7) $$\begin{array}{l} \pi (\tilde{L}|\theta^{*})=\overset{q}{\underset{r=1}{\prod }}[(1-\theta^{*})\Psi ({\tilde{L}}_{r}|\nu_{0})+\theta^{*}\Psi ({\tilde{L}}_{r}|\nu_{1})]. \end{array}$$

Here, $\theta^{*}\in (0,1)$ is a mixing proportion capturing the expected proportion of nonzero ${\tilde{L}}_{r}$ and the multivariate Laplace density $\Psi ({\tilde{L}}_{r}|\nu )$ is given by,

(8) $$\begin{array}{l} \Psi ({\tilde{L}}_{r}|\nu )=\frac{\nu^{N_{r}}e^{-\nu ||{\tilde{L}}_{r}|{|}_{2}}}{2^{N_{r}}\pi^{(N_{r}-1)/2}\Gamma ((N_{r}+1)/2)},\;r=1,\ldots ,q. \end{array}$$

Here, $N_{r}$ denotes the length of the vector ${\tilde{L}}_{r}$ and hence the $SSGL(\nu_{0},\nu_{1},\theta^{*})$ prior adopts to the varying group size in $\tilde{L}$, ensuring that the overall amount of shrinkage remains comparable. In the SSGL priors (5,7), we set $\lambda_{0}>>\lambda_{1}$ (and similarly $\nu_{0}>>\nu_{1}$) so the spike component $\Psi (\gamma_{k}|\lambda_{0})$ (or $\Psi ({\tilde{L}}_{r}|\nu_{0})$) is heavily concentrated around the zero vector. The slab component $\Psi (\gamma_{k}|\lambda_{1})$ (or $\Psi ({\tilde{L}}_{r}|\nu_{1})$) prevents shrinking parameters with large magnitude. This is an advantage of SSGL over other group penalties such as group lasso [59] or its nonconvex extensions such as the group minimax concave penalty (MCP), since SSGL can perform adaptive shrinkage due to the two tuning parameters (spike and slab) controlling the sparsity as opposed to one. Hence, the groups with larger coefficients can be identified due to the minimal shrinkage imposed by the slab component. Another key innovation of the proposed SSGL prior for mixed effect selection is that we enhance flexibility by allowing the fixed and random components to have different degrees of sparsity, accounting for their scale difference via different sets of tuning parameters $\left(\lambda_{0},\lambda_{1}\right)$, and $\left(\nu_{0},\nu_{1}\right)$.

The unknown mixing proportions θ and $\theta^{*}$ are endowed with beta priors,

(9) $$\theta ∼ℬ(a_{0},b_{0}),\theta^{*}∼ℬ(a_{1},b_{1}).$$

Here, $a_{0},b_{0},a_{1},b_{1}>0$ are positive constant hyperparameters. These priors on θ and $\theta^{*}$ render our Bayesian penalty in (5, 7) non‐separable, making the groups $\gamma_{k}$ and ${𝕃}_{r}$ a priori dependent [41, 56], and enabling sharing of information across groups. For particular choices of hyperparameters such as $a_{0}=1,b_{0}=p$ and $a_{1}=1,b_{1}=q$, the SSGL prior has been shown to perform automatic multiplicity adjustment and favor parsimonious models in higher dimensions [60]. For the covariance parameter Ω we use an Inverse‐Wishart prior which is conditionally conjugate,

(10) Ω∼IW(ν,Δ),ν>L−1,Δis positive definite.

Finally, for the error variance $\sigma^{2}$, we use the Inverse‐Gamma prior,

(11) $$\sigma^{2}∼IG(c_{0}/2,d_{0}/2),$$

where $c_{0},d_{0}$ are hyperparameters. We have used $c_{0}=1,d_{0}=1$ throughout this article, which results in a weakly informative prior. Next, based on the model formulation in (4) and the above priors, we illustrate a computationally efficient and scalable Expectation Conditional Maximization (ECM) algorithm that maximizes the posterior distribution, that is, performs a MAP estimation.

### 2.4 ECM Algorithm for MAP Estimation

Let us denote the set of unknown parameters in model (4) and in priors (5, 7, 9, 10, 11) as $\Phi =\{\gamma ,\tilde{L},b,\zeta ,\theta ,\theta^{*},\Omega ,\sigma^{2}\}$. Here $b={\left(b_{1}^{T},\ldots ,b_{n}^{T}\right)}^{T}$ and $\zeta ={\left(\zeta_{1}^{T},\ldots ,\zeta_{n}^{T}\right)}^{T}$. Note that the matrix 𝕃 in model (4) is directly related to $\tilde{L}$ as $\text{vec}(𝕃)=𝕁\tilde{L}$, for a non‐singular ${\left(d^{\prime }q\right)}^{2}\times \frac{d^{\prime }q(d^{\prime }q+1)}{2}$ dimensional matrix 𝕁 [52] which transforms $\tilde{L}$ to vec(𝕃) (vectorization of the matrix 𝕃). Hence, we can rewrite the model (4) as, $Y_{i}={𝕏}_{i}\gamma +(b_{i}^{T}⊗{\mathbb{Z}}_{i})𝕁\tilde{L}+{𝕎}_{i}\zeta_{i}+\epsilon_{i}.$ For obtaining the MAP estimator, the log‐posterior density (up to an additive constant) is given by,

(12) $$\begin{array}{ll} & \log\{\pi (\Phi |Y,𝕏,\mathbb{Z},𝕎)\}\\ & \;=-\frac{N}{2}\log(\sigma^{2})-\overset{n}{\underset{i=1}{\sum }}\frac{||Y_{i}-{𝕏}_{i}\gamma -(b_{i}^{T}⊗{\mathbb{Z}}_{i})𝕁\tilde{L}-{𝕎}_{i}\zeta_{i}|{|}_{2}^{2}}{2\sigma^{2}}\\ & \;-\frac{1}{2}\overset{n}{\underset{i=1}{\sum }}b_{i}^{T}b_{i}+\overset{p}{\underset{k=1}{\sum }}\log\{(1-\theta )\lambda_{0}^{d}e^{-\lambda_{0}\|\gamma_{k}{\|}_{2}}+\theta \lambda_{1}^{d}e^{-\lambda_{1}\|\gamma_{k}{\|}_{2}}\}\\ & \;+\overset{q}{\underset{r=1}{\sum }}\log\{(1-\theta^{*})\nu_{0}^{d}e^{-\nu_{0}\|{\tilde{L}}_{r}{\|}_{2}}+\theta^{*}\nu_{1}^{d}e^{-\nu_{1}\|{\tilde{L}}_{r}{\|}_{2}}\}\\ & \;+(a_{0}-1)\log\theta +(b_{0}-1)\log(1-\theta )\\ & \;+(a_{1}-1)\log\theta^{*}+(b_{1}-1)\log(1-\theta^{*})\\ & \;-\left(\frac{c_{0}+2}{2}\right)\log\sigma^{2}-\frac{d_{0}}{2\sigma^{2}}\\ & \;+\frac{n}{2}\log(\det(\Omega^{-1}))-\frac{1}{2}\overset{n}{\underset{i=1}{\sum }}\zeta_{i}^{T}\zeta_{i}+\frac{\nu +L+1}{2}\log(\det(\Omega^{-1}))\\ & \;-\frac{1}{2}\text{tr}(\Delta \Omega^{-1}). \end{array}$$

Now to perform variable selection for the fixed and random effects, we further introduce latent binary indicators $\tau_{k}\in \{0,1\},k=1,\ldots ,p$ and $\tau_{r}^{*}\in \{0,1\},r=1,\ldots ,q$, which indicates if the effect is coming from the slab component or the spike component. In particular, we posit the following hierarchical formulation for the SSGL priors $SSGL(\lambda_{0},\lambda_{1},\theta )$ and $SSGL(\nu_{0},\nu_{1},\theta^{*})$ as the marginal priors under beta‐Bernoulli priors π(τ|θ) and $\pi (\tau^{*}|\theta^{*})$,

(13) $$\begin{array}{ll} \pi (\gamma |\tau ) & =\overset{p}{\underset{k=1}{\prod }}[(1-\tau_{k})\Psi (\gamma_{k}|\lambda_{0})+\tau_{k}\Psi (\gamma_{k}|\lambda_{1})],\\ & \;\pi (\tau |\theta )=\overset{p}{\underset{k=1}{\prod }}\theta^{\tau_{k}}{\left(1-\theta \right)}^{1-\tau_{k}}, \end{array}$$

and similarly,

(14) $$\begin{array}{ll} \pi (\tilde{L}|\tau^{*}) & =\overset{q}{\underset{r=1}{\prod }}[(1-\theta^{*})\Psi ({\tilde{L}}_{r}|\nu_{0})+\theta^{*}\Psi ({\tilde{L}}_{r}|\nu_{1})],\\ & \;\pi (\tau^{*}|\theta^{*})=\overset{q}{\underset{r=1}{\prod }}{\left(\theta^{*}\right)}^{\tau_{r}^{*}}{\left(1-\theta^{*}\right)}^{1-\tau_{r}^{*}}, \end{array}$$

where $\tau ={\left(\tau_{1},\ldots ,\tau_{p}\right)}^{T}$ and $\tau^{*}={\left(\tau_{1}^{*},\ldots ,\tau_{q}^{*}\right)}^{T}$, are unknown. The augmented log‐posterior $\log\{\pi (\Phi ,\tau ,\tau^{*}|Y,𝕏,\mathbb{Z},𝕎)\}$ is given by:

(15) $$\begin{array}{ll} & \log\{\pi (\Phi ,\tau ,\tau^{*}|Y,𝕏,\mathbb{Z},𝕎)\}\\ & \;=-\frac{N}{2}\log(\sigma^{2})-\overset{n}{\underset{i=1}{\sum }}\frac{||Y_{i}-{𝕏}_{i}\gamma -(b_{i}^{T}⊗{\mathbb{Z}}_{i})𝕁\tilde{L}-{𝕎}_{i}\zeta_{i}|{|}_{2}^{2}}{2\sigma^{2}}\\ & \;-\frac{1}{2}\overset{n}{\underset{i=1}{\sum }}b_{i}^{T}b_{i}+\overset{p}{\underset{k=1}{\sum }}\log\{(1-\tau_{k})\lambda_{0}^{d}e^{-\lambda_{0}\|\gamma_{k}{\|}_{2}}+\tau_{k}\lambda_{1}^{d}e^{-\lambda_{1}\|\gamma_{k}{\|}_{2}}\}\\ & \;+\overset{q}{\underset{r=1}{\sum }}\log\{(1-\tau_{r}^{*})\nu_{0}^{d}e^{-\nu_{0}\|{\tilde{L}}_{r}{\|}_{2}}+\tau_{r}^{*}\nu_{1}^{d}e^{-\nu_{1}\|{\tilde{L}}_{r}{\|}_{2}}\}\\ & \;+\left(a_{0}-1+\overset{p}{\underset{k=1}{\sum }}\tau_{k}\right)\log\theta +\left(b_{0}-1+p-\overset{p}{\underset{k=1}{\sum }}\tau_{k}\right)\log(1-\theta )\\ & \;+(a_{1}-1+\overset{q}{\underset{r=1}{\sum }}\tau_{r}^{*})\log\theta^{*}+\\ & \;(b_{1}-1+q-\overset{q}{\underset{r=1}{\sum }}\tau_{r}^{*})\log(1-\theta^{*})-\left(\frac{c_{0}+2}{2}\right)\log\sigma^{2}-\frac{d_{0}}{2\sigma^{2}}\\ & \;+\frac{n}{2}\log(\det(\Omega^{-1}))-\frac{1}{2}\overset{n}{\underset{i=1}{\sum }}\zeta_{i}^{T}\zeta_{i}+\frac{\nu +L+1}{2}\log(\det(\Omega^{-1}))\\ & \;-\frac{1}{2}\text{tr}(\Delta \Omega^{-1}). \end{array}$$

Treating the indicators $\tau ,\tau^{*}$ as missing data, we follow an ECM algorithm [61] to calculate the expected log‐posterior $Q(\Phi |\Phi^{\left(t-1\right)})=E_{\tau ,\tau^{*}}(log\{\pi (\Phi ,\tau ,\tau^{*}|Y,𝕏,\mathbb{Z},𝕎)\}|\Phi^{\left(t-1\right)})$ given the current parameter estimates at (t−1)th stage in the first step (E‐step) and then iteratively maximize the expected log‐posterior $Q(\Phi |\Phi^{\left(t-1\right)})$ iteratively with respect to the parameters of interest Φ (CM step). The E and CM steps are iterated until convergence. The explicit details of the E and the CM steps are presented below. We also summarize the key steps as an algorithm in Algorithm 1.

ALGORITHM 1 **ECM algorithm for MAP estimation under MuFuMES.**

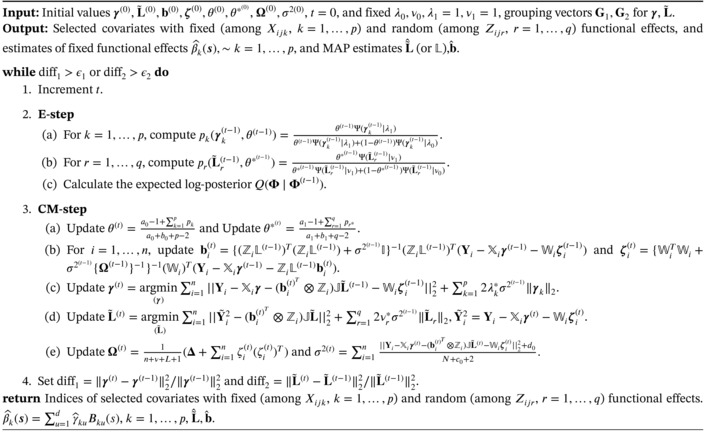

#### 2.4.1 E Step

For calculating the expected log‐posterior $Q(\Phi |\Phi^{\left(t-1\right)})$ based on equation (15) of the paper, we first obtain $E(\tau_{k}|Y,𝕏,\mathbb{Z},𝕎,\Phi^{\left(t-1\right)})$ and $E(\tau_{r}^{*}|Y,𝕏,\mathbb{Z},𝕎,\Phi^{\left(t-1\right)})$.

(16) $$\begin{array}{l} E(\tau_{k}|Y,𝕏,\mathbb{Z},𝕎,\Phi^{\left(t-1\right)})=p_{k}(\gamma_{k}^{\left(t-1\right)},\theta^{\left(t-1\right)}), \end{array}$$

(17) $$\begin{array}{l} p_{k}(\gamma_{k},\theta )=\frac{\theta \Psi (\gamma_{k}|\lambda_{1})}{\theta \Psi (\gamma_{k}|\lambda_{1})+(1-\theta )\Psi (\gamma_{k}|\lambda_{0})},k=1,\ldots ,p. \end{array}$$

Here, $p_{k}(\gamma_{k},\theta )$ is the conditional posterior probability of $\gamma_{k}$ coming from the slab distribution rather than the spike component. For notational simplicity, we denote $p_{k}(\gamma_{k}^{\left(t-1\right)},\theta^{\left(t-1\right)})$ computed given the current ((t−1)‐th stage) estimates as $p_{k}$. Similarly, we obtain

(18) $$\begin{array}{l} E(\tau_{r}^{*}|Y,𝕏,\mathbb{Z},𝕎,\Phi^{\left(t-1\right)})=p_{r}({\tilde{L}}_{r}^{\left(t-1\right)},\theta^{{*}^{\left(t-1\right)}}), \end{array}$$

(19) $$\begin{array}{l} p_{r}({\tilde{L}}_{r},\theta^{*})=\frac{\theta^{*}\Psi ({\tilde{L}}_{r}|\nu_{1})}{\theta^{*}\Psi ({\tilde{L}}_{r}|\nu_{1})+(1-\theta^{*})\Psi ({\tilde{L}}_{r}|\nu_{0})},r=1,\ldots ,q. \end{array}$$

We denote $p_{r}({\tilde{L}}_{r}^{\left(t-1\right)},\theta^{{*}^{\left(t-1\right)}})$ computed given the current ((t−1)‐th stage) estimates as $p_{r}^{*}$. Next, we define $\lambda_{k}^{*}=\lambda_{0}(1-p_{k})+\lambda_{1}p_{k}$ for k=1,…,p and $\nu_{r}^{*}=\nu_{0}(1-p_{r}^{*})+\nu_{1}p_{r}^{*}$ for r=1,…,q. It can be seen that $E_{\tau }(\log\{(1-\tau_{k})\lambda_{0}^{d}e^{-\lambda_{0}\|\gamma_{k}{\|}_{2}}+\tau_{k}\lambda_{1}^{d}e^{-\lambda_{1}\|\gamma_{k}{\|}_{2}}\})=-\lambda_{k}^{*}\|\gamma_{k}{\|}_{2}$ up to an additive constant which does on depend on the parameters in Φ. Similarly, we have $E_{\tau^{*}}(\log\{(1-\theta^{*})\nu_{0}^{d}e^{-\nu_{0}\|{\tilde{L}}_{r}{\|}_{2}}+\theta^{*}\nu_{1}^{d}e^{-\nu_{1}\|{\tilde{L}}_{r}{\|}_{2}})=-\nu_{r}^{*}\|{\tilde{L}}_{r}{\|}_{2}$ up to an additive constant which does not depend on the parameters in Φ. Plugging in these conditional expectations into $Q(\Phi |\Phi^{\left(t-1\right)})=E_{\tau ,\tau^{*}}(\log\{\pi (\Phi ,\tau ,\tau^{*}|Y,𝕏,\mathbb{Z},𝕎)\}|\Phi^{\left(t-1\right)})$ we have,

(20) $$\begin{array}{ll} & Q(\Phi |\Phi^{\left(t-1\right)})\\ & \;=E_{\tau ,\tau^{*}}(\log\{\pi (\Phi ,\tau ,\tau^{*}|Y,𝕏,\mathbb{Z},𝕎)\}|\Phi^{\left(t-1\right)})\\ & \;=C-\frac{N}{2}\log(\sigma^{2})\\ & \;-\overset{n}{\underset{i=1}{\sum }}\frac{||Y_{i}-{𝕏}_{i}\gamma -(b_{i}^{T}⊗{\mathbb{Z}}_{i})𝕁\tilde{L}-{𝕎}_{i}\zeta_{i}|{|}_{2}^{2}}{2\sigma^{2}}\\ & \;-\frac{1}{2}\overset{n}{\underset{i=1}{\sum }}b_{i}^{T}b_{i}-\overset{p}{\underset{k=1}{\sum }}\lambda_{k}^{*}\|\gamma_{k}{\|}_{2}-\overset{q}{\underset{r=1}{\sum }}\nu_{r}^{*}\|{\tilde{L}}_{r}{\|}_{2}\\ & \;+\left(a_{0}-1+\overset{p}{\underset{k=1}{\sum }}p_{k}\right)\log\theta +\left(b_{0}-1+p-\overset{p}{\underset{k=1}{\sum }}p_{k}\right)\log(1-\theta )\\ & \;+\left(a_{1}-1+\overset{q}{\underset{r=1}{\sum }}p_{r^{*}}\right)\log\theta^{*}+\\ & \;\left(b_{1}-1+q-\overset{q}{\underset{r=1}{\sum }}p_{r}^{*}\right)\log(1-\theta^{*})-\left(\frac{c_{0}+2}{2}\right)\log\sigma^{2}-\frac{d_{0}}{2\sigma^{2}},\\ & \;+\frac{n}{2}\log(\det(\Omega^{-1}))-\frac{1}{2}\overset{n}{\underset{i=1}{\sum }}\zeta_{i}^{T}\zeta_{i}+\frac{\nu +L+1}{2}\log(\det(\Omega^{-1}))\\ & \;-\frac{1}{2}\text{tr}(\Delta \Omega^{-1}), \end{array}$$

where C is a constant not depending on the parameters.

#### 2.4.2 CM Step

In the CM step, we maximize $Q(\Phi |\Phi^{\left(t-1\right)})$ in (5) with respect to the parameters $\Phi =\{\gamma ,\tilde{L},b,\zeta ,\theta ,\theta^{*},\Omega ,\sigma^{2}\}$ through two iterative steps.

##### 2.4.2.1 Step 1

In the first CM step, we optimize $Q(\Phi |\Phi^{\left(t-1\right)})$ with respect to $\left(\theta ,\theta^{*},b,\zeta \right)$ holding $\left(\gamma ,\tilde{L},\Omega ,\sigma^{2}\right)$ fixed at the current value.

$$(\theta^{\left(t\right)},\theta^{*(t)},b^{\left(t\right)},\zeta^{\left(t\right)})=\underset{\left(\theta ,\theta^{*},b,\zeta \right)}{\text{argmax}}\{Q(\Phi |\Phi^{\left(t-1\right)})\}{|}_{\gamma^{\left(t-1\right)},{\tilde{L}}^{\left(t-1\right)},\Omega^{\left(t-1\right)},\sigma^{2^{\left(t-1\right)}}}.$$

##### 2.4.2.2 Step 2

In the second CM step, we optimize $Q(\Phi |\Phi^{\left(t-1\right)})$ with respect to $\left(\gamma ,\tilde{L},\Omega ,\sigma^{2}\right)$ holding $\left(\theta ,\theta^{*},b,\zeta \right)$ fixed at the updated current value.

$$(\gamma^{\left(t\right)},{\tilde{L}}^{\left(t\right)},\Omega^{\left(t\right)},\sigma^{2^{\left(t\right)}})=\underset{\left(\gamma ,\tilde{L},\Omega ,\sigma^{2}\right)}{\text{argmax}}\{Q(\Phi |\Phi^{\left(t-1\right)})\}{|}_{\theta^{\left(t\right)},\theta^{*(t)},b^{\left(t\right)},\zeta^{\left(t\right)}}.$$

Next, we present the computational details for performing steps 1 and 2. For step 1, based on $Q(\Phi |\Phi^{\left(t-1\right)})$ in Equation (5), we see that both $\theta^{\left(t\right)}$ and $\theta^{{*}^{\left(t\right)}}$ have close form updates:

(21) $$\begin{array}{l} \theta^{\left(t\right)}=\frac{a_{0}-1+\sum_{k=1}^{p}p_{k}}{a_{0}+b_{0}+p-2}, \end{array}$$

and

(22) $$\begin{array}{l} \theta^{{*}^{\left(t\right)}}=\frac{a_{1}-1+\sum_{r=1}^{q}p_{r^{*}}}{a_{1}+b_{1}+q-2}. \end{array}$$

The random effect parameter $b_{i}$ (for i=1,…,n) based on (5) are updated as,

(23) $$\begin{array}{ll} b_{i}^{\left(t\right)} & ={\left\{{\left({\mathbb{Z}}_{i}{𝕃}^{\left(t-1\right)}\right)}^{T}({\mathbb{Z}}_{i}{𝕃}^{\left(t-1\right)})+\sigma^{2^{\left(t-1\right)}}𝕀\right\}}^{-1}{\left({\mathbb{Z}}_{i}{𝕃}^{\left(t-1\right)}\right)}^{T}\\ & \;(Y_{i}-{𝕏}_{i}\gamma^{\left(t-1\right)}-{𝕎}_{i}\zeta_{i}^{\left(t-1\right)}),i=1,\ldots ,n, \end{array}$$

where 𝕃 is as in model (4) of the paper and we have $vec({𝕃}^{\left(t-1\right)})=𝕁{\tilde{L}}^{\left(t-1\right)}$. The random effect parameter $\zeta_{i}$ (for i=1,…,n) based on (5) are then updated as,

(24) $$\begin{array}{ll} \zeta_{i}^{\left(t\right)} & ={\left\{{𝕎}_{i}^{T}{𝕎}_{i}+\sigma^{2^{\left(t-1\right)}}{\left\{\Omega^{\left(t-1\right)}\right\}}^{-1}\right\}}^{-1}{\left({𝕎}_{i}\right)}^{T}(Y_{i}-{𝕏}_{i}\gamma^{\left(t-1\right)}\\ & \;-{\mathbb{Z}}_{i}{𝕃}^{\left(t-1\right)}b_{i}^{\left(t\right)}),i=1,\ldots ,n. \end{array}$$

For the step 2 of the CM algorithm, we first update γ by maximizing the expected log‐posterior (5) with respect to γ holding $\left(\theta^{\left(t\right)},\theta^{*(t)},b^{\left(t\right)},\zeta^{\left(t\right)}\right)$ and ${\tilde{L}}^{\left(t-1\right)},\Omega^{\left(t-1\right)},\sigma^{2^{\left(t-1\right)}}$ fixed. This optimization can be reduced to,

(25) $$\begin{array}{ll} \gamma^{\left(t\right)} & =\underset{\left(\gamma \right)}{\text{argmax}}-\overset{n}{\underset{i=1}{\sum }}\frac{||Y_{i}-{𝕏}_{i}\gamma -(b_{i}^{{\left(t\right)}^{T}}⊗{\mathbb{Z}}_{i})𝕁{\tilde{L}}^{\left(t-1\right)}-{𝕎}_{i}\zeta_{i}^{\left(t\right)}|{|}_{2}^{2}}{2\sigma^{2^{\left(t-1\right)}}}\\ & \;-\overset{p}{\underset{k=1}{\sum }}\lambda_{k}^{*}\|\gamma_{k}{\|}_{2}\\ & =\underset{\left(\gamma \right)}{\text{argmin}}\;\overset{n}{\underset{i=1}{\sum }}||Y_{i}-{𝕏}_{i}\gamma -(b_{i}^{{\left(t\right)}^{T}}⊗{\mathbb{Z}}_{i})𝕁{\tilde{L}}^{\left(t-1\right)}-{𝕎}_{i}\zeta_{i}^{\left(t\right)}|{|}_{2}^{2}\\ & \;+\overset{p}{\underset{k=1}{\sum }}2\lambda_{k}^{*}\sigma^{2^{\left(t-1\right)}}\|\gamma_{k}{\|}_{2}\\ & =\underset{\left(\gamma \right)}{\text{argmin}}\;\overset{n}{\underset{i=1}{\sum }}||{\tilde{Y}}_{i}^{1}-{𝕏}_{i}\gamma |{|}_{2}^{2}+\overset{p}{\underset{k=1}{\sum }}2\lambda_{k}^{*}\sigma^{2^{\left(t-1\right)}}\|\gamma_{k}{\|}_{2}, \end{array}$$

where ${\tilde{Y}}_{i}^{1}=Y_{i}-(b_{i}^{{\left(t\right)}^{T}}⊗{\mathbb{Z}}_{i})𝕁{\tilde{L}}^{\left(t-1\right)}-{𝕎}_{i}\zeta_{i}^{\left(t\right)}$. This can now be identified as a group‐LASSO problem [59], with adaptive group‐specific weights. We use the coordinate descent algorithm by [62] for the above optimization. Next, we update $\tilde{L}$ by maximizing (5) with respect to $\tilde{L}$, holding $\left(\theta^{\left(t\right)},\theta^{*(t)},b^{\left(t\right)},\zeta^{\left(t\right)}\right)$ and $\gamma^{\left(t\right)},\Omega^{\left(t-1\right)},\sigma^{2^{\left(t-1\right)}}$ fixed. This optimization similarly reduces to the following group‐LASSO type problem with varying group size and adaptive weights:

(26) $$\begin{array}{ll} {\tilde{L}}^{\left(t\right)} & =\underset{\left(\tilde{L}\right)}{\text{argmax}}-\overset{n}{\underset{i=1}{\sum }}\frac{||Y_{i}-{𝕏}_{i}\gamma^{\left(t\right)}-(b_{i}^{{\left(t\right)}^{T}}⊗{\mathbb{Z}}_{i})𝕁\tilde{L}-{𝕎}_{i}\zeta_{i}^{\left(t\right)}|{|}_{2}^{2}}{2\sigma^{2^{\left(t-1\right)}}}\\ & \;-\overset{q}{\underset{r=1}{\sum }}\nu_{r}^{*}\|{\tilde{L}}_{r}{\|}_{2}\\ & =\underset{\left(\tilde{L}\right)}{\text{argmin}}\;\overset{n}{\underset{i=1}{\sum }}||{\tilde{Y}}_{i}^{2}-(b_{i}^{{\left(t\right)}^{T}}⊗{\mathbb{Z}}_{i})𝕁\tilde{L}|{|}_{2}^{2}\\ & \;+\overset{q}{\underset{r=1}{\sum }}2\nu_{r}^{*}\sigma^{2^{\left(t-1\right)}}\|{\tilde{L}}_{r}{\|}_{2}, \end{array}$$

where ${\tilde{Y}}_{i}^{2}=Y_{i}-{𝕏}_{i}\gamma^{\left(t\right)}-{𝕎}_{i}\zeta_{i}^{\left(t\right)}$. Finally, Ω and $\sigma^{2}$ are updated in Step 2, with closed‐form updates given by:

(27) $$\begin{array}{l} \Omega^{\left(t\right)}=\frac{1}{n+\nu +L+1}(\Delta +\overset{n}{\underset{i=1}{\sum }}\zeta_{i}^{\left(t\right)}{\left(\zeta_{i}^{\left(t\right)}\right)}^{T}), \end{array}$$

(28) $$\begin{array}{l} \sigma^{2^{\left(t\right)}}=\overset{n}{\underset{i=1}{\sum }}\frac{||Y_{i}-{𝕏}_{i}\gamma^{\left(t\right)}-(b_{i}^{{\left(t\right)}^{T}}⊗{\mathbb{Z}}_{i})𝕁{\tilde{L}}^{\left(t\right)}-{𝕎}_{i}\zeta_{i}^{\left(t\right)}|{|}_{2}^{2}+d_{0}}{N+c_{0}+2}. \end{array}$$

Here, $N=m(\sum_{i=1}^{n}J_{i})$ denotes the total number of observations.

Remark 1 In the context of the multilevel functional mixed effects model considered in this paper, high‐dimensionality refers to the number of parameters that need to be estimated in relation to the cluster size. Note that the dimensionality of the number of unknown parameters in Φ is driven by all the pd spline basis coefficients for fixed effects γ and the number of variance‐covariance parameters in the matrix 𝔻 or equivalently 𝕃, which has $d^{\prime }q\times (d^{\prime }q+1)/2$ or 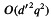 parameters, and this can be significantly larger than the cluster size n in the multilevel functional mixed effects model.

### 2.5 Choice of Tuning Parameters

The proposed MuFuMES method illustrated above involves several tuning parameters: $\lambda_{0}>>\lambda_{1}$ and $\nu_{0}>>\nu_{1}$, which are the spike and slab parameters corresponding to the fixed effect and random effect variance components, respectively. We fix the slab parameters in the SSGL priors $\text{SSGL}(\lambda_{0},\lambda_{1},\theta )$ and $\text{SSGL}(\nu_{0},\nu_{1},\theta^{*})$ to be $\lambda_{1}=1,\nu_{1}=1$. The spike parameters are chosen in a data‐driven way, based on a two‐dimensional grid search, from a grid of decreasing $\lambda_{0}$ values and $\nu_{0}$ values [41]. To accommodate the varying group sizes in ${\tilde{L}}_{r}$, we additionally scale up $\nu_{0}$ by $\sqrt{N_{r}}$ for each group while implementing the group‐LASSO optimization [56] within the CM step. The optimal $\left(\lambda_{0},\nu_{0}\right)$ combination is chosen based on a BIC‐type criterion [41, 51] defined as:

(29) $$BIC(\lambda_{0},\nu_{0})=-2\overset{n}{\underset{i=1}{\sum }}\ell (Y_{i},\hat{\Phi })+log(N)\times df_{\lambda_{0},\nu_{0}}.$$

Here $\hat{\Phi }$ denotes the MAP estimator, ℓ is the marginal log‐likelihood of $Y_{i}$ based on model (4) with marginal covariance $\sum_{i}={\mathbb{Z}}_{i}𝔻{\mathbb{Z}}_{i}^{T}+{𝕎}_{i}\Omega {𝕎}_{i}^{T}+\sigma^{2}𝕀$. Here, $df_{\lambda_{0},\nu_{0}}$ denotes the total number of nonzero elements in the MAP estimator $\hat{\gamma }$ and $\hat{\tilde{L}}$. The hyperparameters are set to $a_{0}=1,b_{0}=p$ and $a_{1}=1,b_{1}=q$ favoring parsimonious models. We also set the hyperparameters ν=L+2,Δ=𝕀, and $c_{0}=d_{0}=1$, resulting in weakly informative priors. The number of basis functions $d,d^{\prime }$ controls the smoothness of the functional coefficients. In this article, we have used a moderate number of basis functions to control the smoothness of the fixed and random functional effects using a truncated basis approach [63]. Note that the BIC criterion $BIC(\lambda_{0},\nu_{0})$ implicitly depends on $d,d^{\prime }$ and can also be considered as $BIC(\lambda_{0},\nu_{0},d,d^{\prime })$. In the application, we have chosen $d^{\prime }d^{\prime }$ in a data‐driven way based on this BIC criterion along with $\lambda_{0},\nu_{0}$.

## 3 Simulation Study

We investigate the performance of the proposed MuFuMES method using numerical simulations. We generate observations following a multilevel functional mixed effects model (denoted as Scenario A) given by,

(30) $$Y_{ij}(s)=\overset{8}{\underset{k=1}{\sum }}X_{ijk}\beta_{k}(s)+\overset{8}{\underset{r=1}{\sum }}Z_{ijr}u_{ir}(s)+v_{ij}(s)+\epsilon_{ij}(s),s\in [0,1],$$

for clusters i=1,…,n and replications $j=1,\ldots ,J_{i}$. In this model, we consider p=8 covariates for functional fixed effects (including a fixed intercept) and q=8 covariates for functional random effects (including a random intercept). The fixed effects coefficient functions are given by $\beta_{1}(s)=8sin(2\pi s)$ (intercept), $\beta_{2}(s)=2\phi (s,0.6,0.15^{2})$, $\beta_{3}(s)=2.5\phi (s,0.6,0.15^{2})$, $\beta_{4}(s)=3cos(2\pi s)$, $\beta_{5}(s)=5sin(2\pi s)+5cos(2\pi s)$ and $\beta_{k}(s)=0$ for k=6,7,…,8. Here, we denote by $\phi (s,a,b^{2})$ the density at s for Normal distribution with mean a and variance $b^{2}$. So, only the first 5 fixed effect covariates are relevant. The fixed effect covariates $X_{ijk}$ are independently generated from a $𝒩(0,2^{2})$ distribution for k=2,…,8 and $X_{ij1}=1$ (for all i,j) corresponds to the intercept. The random effect covariates $Z_{ijr}$ are exactly the same as the fixed effect covariates $X_{ijk}$ [51] in this scenario. The functional random effects are given by $u_{i1}(s)=c_{i1}sin(2\pi s)+d_{i1}cos(2\pi s)$ (random intercept), where $c_{i1}∼𝒩(0,3^{2}\sigma_{B}^{2}),d_{i1}∼𝒩(0,1.5^{2}\sigma_{B}^{2})$. Similarly, $u_{i4}(s)=c_{i4}sin(2\pi s)+d_{i4}cos(2\pi s)+e_{i4}sin(\pi s)+f_{i4}cos(\pi s)$, where $c_{i4}∼𝒩(0,1.5^{2}\sigma_{B}^{2}),d_{i4}∼𝒩(0,0.75^{2}\sigma_{B}^{2}),e_{i4}∼𝒩(0,0.5^{2}\sigma_{B}^{2}),f_{i4}∼𝒩(0,0.25^{2}\sigma_{B}^{2})$. The rest of the functional random effects $u_{ik}(s)$ are considered to be zero. Hence, only the first and the fourth random effects of the covariates are important. We set $\sigma_{B}^{2}$ based on $SNR_{B}=0.5$, where $SNR_{B}$ is the standard deviation of the fixed effects functions divided by the standard deviation of the random effects [27, 42]. The cluster‐subject‐level functional random effect $v_{ij}(s)$ is given by $v_{ij}(s)=v_{ij1}\sin(\pi s)+v_{ij2}\cos(\pi s)$, where $v_{ij1}∼𝒩(0,0.8^{2}\sigma_{S}^{2}),v_{ij2}∼𝒩(0,0.4^{2}\sigma_{S}^{2})$. We set $\sigma_{S}^{2}$ based on $SNR_{S}=2$, where $SNR_{S}$ is the standard deviation of the fixed effects functions divided by the standard deviation of the cluster‐visit level random effects. The random errors $\epsilon_{ij}(s)∼𝒩(0,\sigma_{\epsilon }^{2})$, where $\sigma_{\epsilon }$ is chosen based on a signal to noise ratio of $SNR_{\epsilon }=4$, which represents the standard deviation of the linear predictors (fixed and random predictors combined) divided by the standard deviation of the noise $\sigma_{\epsilon }$. The functional response $Y_{ij}(s)$ is observed on a grid of m=10 equidistant time points in S=[0,1]. Cluster size n∈{25,50,100} is considered for this scenario, and $J_{i}=J=10$ replications are considered within each cluster. We generate 100 replicated data sets to assess the performance of the proposed variable selection method. We also explore an additional simulation scenario (Scenario B), where the fixed and random effect covariates differ, and a high‐dimensional scenario (Scenario C), with p=100 functional fixed effects, cluster size n=25 (ensuring p>>n), and q=10 functional random effects. The details of these additional scenarios are presented in appendix A of the Supporting Information.

### 3.1 Simulation Results

#### 3.1.1 Scenario A

We evaluate the performance of the proposed MuFuMES method in terms of selection accuracy and estimation accuracy. We have used $d=d^{\prime }=10$ cubic B‐spline basis functions for modelling the fixed and random functional coefficients for n=25,50 and $d=d^{\prime }=12$ for n=100, allowing the basis dimension to grow with the sample size. We also compare the performance of MuFuMES to existing group selection methods that can handle the multilevel functional mixed effects model (1) or group‐selection in its longitudinal mixed effects model representation in (3). Among existing candidates with available implementation for comparison, we identified. (I) The glmmLasso R package [64] which can perform fixed effect selection in linear mixed models with an $L_{1}$ penalization. However, this method cannot handle this scenario since it can only do fixed effect selection and does not impose group penalization, which is essential for selection of functional effects. (II) The grpreg R package [62], which can do group selection of the fixed effects using group LASSO [59], but is unable to handle random effects or account for clustering in the data. We use the longitudinal mixed effects model representation in (3) for the selection of the functional fixed effects and compare its performance to the proposed MuFuMES for fixed effect selection. (III) The glmmPen R package [55] which uses a Monte Carlo Expectation Conditional Minimization (MCECM) algorithm for fixed and random effect selection, but this is again not directly applicable or comparable to our case as this does not handle group selection of fixed effects (or structured group selection of random effects). As an example, with a 80‐dimensional fixed effect parameters (γ) and a 80‐dimensional random effect parameter $\eta_{i}$ leading to 3240 covariance parameters in 𝕃, we found a very high computational cost of glmmPen method (e.g., the method did not converge for a single replication after running 24 h) with cluster size cluster size n=25 and total number of observations N=2500. This is expected, as this method was not developed for functional mixed‐effect selection. Hence, after reviewing, we compare the selection and estimation performance of the proposed method to its closest competitor (II) the fixed effect selection performance from group LASSO implemented using grpreg. Table 1 reports the selection performance of MuFuMES for the selection of the fixed effects and random effects in terms of true positive and false positive rates. The performance of group LASSO is also reported for the fixed effects as a competing method. The tuning parameters of the MuFuMES method are chosen based on the BIC criterion (16) and the tuning parameters of group LASSO are chosen based on a 10‐fold cross‐validation [62].

**TABLE 1:** Average true positive rate (TP), false positive rate (FP) for fixed (TPF, FPF) and random (TPR, FPR) effects of the MuFuMES method, Scenario A. The performance of group LASSO is reported in parentheses for the fixed (TPF, FPF) effects.

Sample Size	TPF	FPF	TPR	FPR
n=25	0.93 (0.998)	0.003 (0.64)	0.98	0
n=50	0.99 (0.996)	0(0.76)	1	0.002
n=100	0.99 (1)	0 (0.73)	0.99	0.003

**FIGURE 2:**
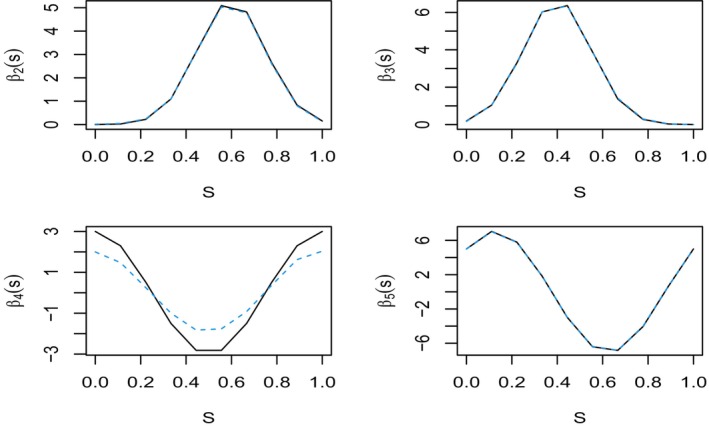
Displayed are the true (solid) and M.C mean (dashed) of estimated functional slopes $\beta_{k}(s)$ (k=2,…,5) from MuFuMES, n=100.

The proposed MuFuMES method is again seen to have a negligible false positive rate (<5% in all cases) and a high true positive rate for both fixed and random functional effects across all the sample sizes. However, the naive group LASSO can be seen to have a very high false positive rate for the fixed effects, reported previously in [36]. To assess the estimation performance, we again display the Monte Carlo (MC) mean estimates (averaged estimated coefficient function over 100 replications) of the functional fixed effect slopes $\beta_{k}(s)$ (k=2,3,4,5) in Figure 2 for sample size n=100 for this scenario. The estimated coefficient functions closely capture the true effects in most cases. Interestingly, in this scenario, we observe the estimated fixed effect $\beta_{4}(s)$ to be more biased than the other estimates, which might be due to the covariate $X_{ij4}$ having both a fixed and a random slope.

We report the mean integrated squared error (MISE) of the functional fixed effect slope estimates from the MuFuMES method in Table 2. We compare these performances with the estimates from the group LASSO‐based approach, which only selects fixed effects and does not account for random effects and hence clustering in the data.

**TABLE 2:** Mean integrated squared error (MISE) of the functional fixed effect slope estimates from the MuFuMES method, scenario B. The performance of group LASSO is reported in parentheses.

Sample Size	MISE $\beta_{2}(\cdot )$	MISE $\beta_{3}(\cdot )$	MISE $\beta_{4}(\cdot )$	MISE $\beta_{5}(\cdot )$
n=25	0.19 (1.25)	0.18 (1.05)	4.40 (4.46)	0.20 (1.37)
n=50	0.08 (0.526)	0.07 (0.540)	2.08 (3.24)	0.09 (0.55)
n=100	0.04 (0.29)	0.04 (0.30)	1.61 (1.79)	0.04 (0.28)

We can observe that properly accounting for the random effects and clustering in our MuFuMES method dramatically improves the estimation performance of the fixed effect parameters in most cases, as compared to naive group LASSO. Interestingly, even for the covariate $X_{ij4}$, which has both a fixed and a random slope, the estimation performance of the MuFuMES estimator $\hat{\beta_{4}}(\cdot )$ is comparable or better than the group LASSO estimator, particularly for smaller sample sizes.

The results from the additional simulation scenarios B, C are presented in appendix A of the Supporting Information. Overall, our simulation results illustrate that the proposed MuFuMES method is able to select the true fixed and random functional effects with high accuracy and also provides an impressive estimation performance by accounting for clustering in the data.

## 4 NHANES 2011‐12 Case Study

We consider identifying and investigating the age and race‐specific heterogeneity in the effect of key demographic, lifestyle, and socioeconomic covariates, on the diurnal functional patterns of physical activity (PA) among adults based on the accelerometer data from the 2011 to 2012 cohort of NHANES. The NHANES is a nationally representative sample of the non‐institutionalized US population and provides a broad range of descriptive summaries related to health and nutrition. In addition, in NHANES 2011‐2012, accelerometer data were collected using the wrist‐worn ActiGraph GT3X+ accelerometer (ActiGraph of Pensacola, FL). Participants were asked to wear the physical activity monitor continually for seven full days and remove it on the morning of the 9th day. We focus on the minute‐level accelerometer data, which reports individuals' acceleration in Monitor Independent Movement Summary (MIMS) unit, an open‐source, device‐independent universal summary metric [49]. Particularly, we consider the MIMS triaxial value (sum of X,Y,Z axis MIMS) reported per minute as the measure of physical activity. Our final sample for analysis consists of 3402 adults (aged more than 20 years) with available physical activity data (physical activity monitoring available at least ten hours per day for at least four days) and covariate information (reported in Supporting Information: Table S3 of appendix B). We considered gender, body mass index (BMI), healthy eating index (HEI) score [65] as a measure of diet quality (ranging from 0‐100, higher indicating better), combined grip strength (potential indicator of frailty, [66]), and income quantified by the ratio of family income to poverty (INDFMPIR) (a measure of income relative to the poverty threshold, with higher value indicating higher income relative to the poverty line) as potential drivers of physical activity across the lifespan, which also might show race and age‐specific heterogeneity. Table S1 in the Supporting Information also presents the descriptive statistics of this sample.

As illustrated in Figure 1 there exists considerable heterogeneity in the diurnal PA patterns among the six age‐groups (20−30,30−40,…,70+) and the six reported races/ethnicities. In this article, we are interested in identifying the key drivers of diurnal patterns of PA and also identifying the covariates having cluster‐specific (age or race‐specific) heterogeneity in their effects on diurnal PA patterns. We define our outcome as $Y_{ij}(s)$ as the daily MIMS at minute s for the j th subject within cluster i=1,2,…,36 (age‐by‐race groups). This is calculated based on averaging all the available MIMS at time s across days for the subject j within cluster i, that is, averaging ${\left\{Y_{ijd}(s)\right\}}_{d=1}^{7}$ to represent the typical daily pattern of PA.

Since, the raw minute‐level MIMS profile can be noisy, to extract and understand smooth diurnal patterns of PA we pre‐smooth the data [37] using 1 h windows, resulting in hourly observations ${\left\{Y_{ij}(s_{1}),Y_{ij}(s_{2}),\ldots ,Y_{ij}(s_{m})\right\}}_{i=1,j=1}^{36,J_{i}}$, m=24 and ${𝒯}_{m}=\{s_{1},s_{2},\ldots ,s_{m}\}$ are equi‐spaced grid points between 12.30 a.m. (midpoint of the first window) to 11.30 p.m. (midpoint of last window). This also helps in reducing the computational complexity of handling minute‐level data in functional models (see [27] for a fast multi‐level estimation approach) and the additional complexity coming from our proposed SSGL‐based MuFuMES method with increasing m. We consider the following multilevel functional mixed effects model for the diurnal PA $Y_{ij}(s)$:

(31) $$\begin{array}{l} Y_{ij}(s)=\overset{11}{\underset{k=1}{\sum }}X_{ijk}\beta_{k}(s)+\overset{8}{\underset{r=1}{\sum }}Z_{ijr}u_{ir}(s)+v_{ij}(s)+\epsilon_{ij}(s). \end{array}$$

Here, $X_{ij1}$ is the column corresponding to the fixed functional intercept, $\left(X_{ij2},X_{ij3},X_{ij4},X_{ij5},X_{ij6}\right)$ corresponds to the covariates BMI, Gender (female), INDFMPIR, MGDCGSZ (grip strength), HEI, respectively, for the j th replication in the i th cluster (age‐by‐race). Additionally, the covariates $X_{ijk}∼𝒩(0,1),k=7,8,\ldots ,11$ are generated as pseudo‐covariates and added as fixed effect covariates to assess the performance of the proposed variable selection method [67, 68]. Similarly, $Z_{ij1}$ is the column corresponding to the random functional intercept, $\left(Z_{ij2},Z_{ij3},Z_{ij4},Z_{ij5},Z_{ij6}\right)$ corresponds to the covariates BMI, Gender (female), INDFMPIR, MGDCGSZ (grip strength), HEI respectively for the j th replication in the i th cluster. We also add pseudo‐covariates $Z_{ij7}=X_{ij7},Z_{ij8}=X_{ij8}$ to further assess the selection performance. We apply the proposed MuFuMES method to select the important fixed and random functional effects. All the continuous covariates are standardized before applying MuFuMES. The optimal number of basis functions, and the spike parameters $\left(\lambda_{0},\nu_{0}\right)$ were chosen using a grid‐search based on the proposed BIC criterion in Section 2.5. The optimally selected values of the spike parameters were $\lambda_{0}=350,\nu_{0}=30$, and we used 7 cubic B‐spline basis functions to model the functional fixed intercept and 5 cubic B‐spline basis functions to model the functional fixed and random slope parameters (as well as the random functional intercept). The selection result from MuFuMES is reported in Table 3.

**TABLE 3:** Selection of fixed and random functional effects in the NHANES application from the MuFuMES method. Variables being selected are indicated by selection = 1 and 0 otherwise.

Fixed	Selection	Random	Selection
Intercept	1	Intercept	1
BMI	1	BMI	1
Gender	1	Gender	1
INDFMPIR	1	INDFMPIR	1
MGDCGSZ	1	MGDCGSZ	1
HEI	0	HEI	1
$X_{7}$ (pseudo)	0	$Z_{7}$ (pseudo)	0
$X_{8}$ (pseudo)	0	$Z_{8}$ (pseudo)	0
$X_{9}$ (pseudo)	0		
$X_{10}$ (pseudo)	0		
$X_{11}$ (pseudo)	0		

**FIGURE 3:**
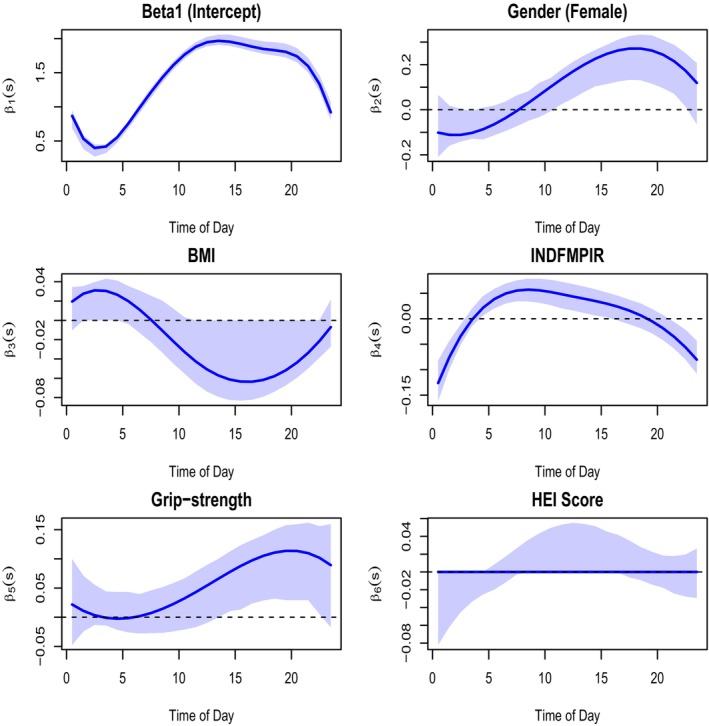
Estimated functional fixed effects in the NHANES application from the MuFuMES method (solid line) and bootstrap‐based 95% point‐wise confidence intervals (shaded region).

We observe that the variables BMI, Gender, INDFMPIR (ratio of family income to poverty), and MGDCGSZ (grip strength) are selected as important functional fixed effects (along with the fixed effect functional intercept), highlighting these variables influence the diurnal PA patterns across the lifespan and all races. Among the functional random effects, first, the intercept is selected, underlining that there exists age and race‐specific heterogeneity in the diurnal PA patterns. Furthermore, we observe that BMI, Gender, INDFMPIR (ratio of family income to poverty), MGDCGSZ (grip strength), and HEI (healthy eating index) exhibit age and race‐specific heterogeneity in their effects on the diurnal PA patterns. All the pseudo‐variables (fixed and random) are discarded by the proposed MuFuMES method, highlighting its robust selection performance. We display the estimated functional fixed effects for the selected covariates in Figure 3 (solid line, for HEI score, the estimate is zero). We also provide a measure of uncertainty of the estimated functional effects using a resampling cluster bootstrap approach [27, 69]. The bootstrap estimates are obtained conditional on the chosen tuning parameters in the main application (model 31), and we use percentile bands to construct the 95%point‐wise confidence intervals.

We observe that the fixed effect intercept $\beta_{1}(s)$ captures the overall diurnal pattern of PA. Females can be seen to have a higher diurnal PA during the daytime (positive $\beta_{2}(s)$ after 8 a.m.) and lower PA during the night compared to the males [2, 46]. A higher BMI is observed to be associated (negative $\beta_{3}(s)$) with a lower diurnal PA [70] during the day and a higher PA during night (disrupted sleep) across all age‐groups and races. A higher income is observed to be associated (positive $\beta_{4}(s)$) with a higher diurnal PA during the day between 5 a.m. – 8 p.m. and a lower PA during the night (better sleep) across all age‐groups and races [71, 72]. A higher grip strength is found to be associated [73] with a higher diurnal PA during the afternoon across all age groups and races. For HEI, the effect was not selected by the proposed method, and the confidence intervals illustrate that the association is not significant. Thus, MuFuMES can identify the key drivers of diurnal PA and estimate their dynamic effects, while also identifying the key factors exhibiting age and race‐specific heterogeneity in their effects on the diurnal PA.

It is also possible with MuFuMES to quantify and understand these age and race‐specific heterogeneous effects. For illustration, we display the predicted diurnal PA trajectories from the MuFuMES across the 36 age‐by‐race groups arising from the interaction of six age groups (20−30,30−40,…,70+) and six races (MA=1,OH=2,NHW=3,NHB=4,NHA=6,OR=7, see Supporting Information: Table S1 for description) in Supporting Information: Figures S2–S37. For each of the age‐by‐race clusters, the predicted trajectories for male (reference group) are given by ${\hat{\beta }}_{1}(s)$ for the fixed effect prediction and ${\hat{\beta }}_{1}(s)+{\hat{\beta }}_{1i}(s)$ for the mixed effects prediction (since continuous variables were standardized and hence centered), with the other covariates being held at their average values across the sample. We observe considerable age and race‐specific heterogeneity in the predicted diurnal PA patterns among males. For example, among the Mexican Americans (MA), the age groups 20−30,30−40,40−50 have a higher diurnal PA compared to the average diurnal PA, the age group 50−60 has a dip in the evening (Figure S4), and the age groups 60−70,70+ have considerably lower late afternoon‐evening PA compared to the average population (Figures S5, S6). Similar heterogeneity but possibly different patterns can also be seen among the other races. For example, among the non‐Hispanic white population, the age groups 20−30,30−40,40−50 have a lower diurnal PA (Figures S13–S15) compared to the average population in the morning‐afternoon (4 p.m.) but have a higher diurnal PA in the evening (after 4 p.m.). Interestingly, this trend begins to get reversed in the older ages, and the older white males (age 70+) can be seen to consistently have a lower predicted PA compared to the average population, particularly later in the day (after 12 p.m.). These observations also match with observed diurnal PA trajectories in Figure 1.

MuFuMES is able to successfully identify the key drivers of diurnal PA across the lifespan among all races, and also identify and understand the age and race‐specific heterogeneities in the effect of these drivers on the diurnal patterns of physical activity.

## 5 Discussion

In this article, we have proposed a new method (MuFuMES) for variable selection of fixed and random effects in multilevel functional mixed effects models. To the best of our knowledge, this is the first work exploring variable selection of fixed and random effects in multilevel functional mixed effects models. With the use of spike‐and‐slab group lasso (SSGL) priors on the B‐spline basis coefficients, a computationally efficient MAP estimation approach is employed for mixed effect selection and estimation through an Expectation Conditional Maximization (ECM) algorithm. Numerical analysis using simulation have shown a satisfactory selection accuracy of the variable selection method for both fixed and random functional effects. The method is applied on the NHANES 2011‐12 accelerometer data to identify gender, BMI, poverty income ratio, and grip strength as the key drivers of diurnal PA across the lifespan among all races. The estimated diurnal functional effects highlight the dynamic association of PA with these factors and could be useful for designing time‐of‐day specific PA interventions [74]. Additionally, the method also identifies BMI, gender, poverty income ratio, grip strength, and healthy eating index (HEI) score as the key factors exhibiting age and race‐specific heterogeneity in their effects on the diurnal PA.

Multiple research directions could be explored based on our current work. In this article, we have followed a truncated basis approach [63] and used a moderate number of basis functions to control the smoothness of the fixed and random functional effects, chosen in the application using a BIC criterion. An alternative could be to use a reparametrization [36] following P‐splines commonly used in Bayesian functional regression [24], which can allow a fixed but high number of basis functions. The smoothness can be controlled explicitly with a fixed smoothing parameter (e.g., $\alpha_{smooth}=0.9$ in [24]). This can potentially reduce the computational complexity of choosing the number of basis functions.

In this article, we have focused on the linear effects of the multilevel predictors. This could be extended to accommodate nonlinear effects of the predictors [37] or interaction effects through single index type models [38]. In this article, we have primarily focused on the selection and estimation of the model components in a multilevel functional mixed effects model. The proposed method can be computationally intensive when working with ultra‐dense (e.g., minute‐level activity counts) functional data. If interested in fixed effects inference and estimation, fast and scalable methods, e.g., [27, 29] based on univariate mixed effects models as building blocks, would serve as suitable candidates. For scalable inference on random effects (without selection), recent inferential methods for prediction based on functional random effects [75] would be appealing. We have used a resampling cluster bootstrap strategy to quantify the uncertainty of the fixed effect estimates. Future work would benefit from exploration of uncertainty quantification and inference while doing simultaneous selection in the MuFuMES based on efficient Markov chain Monte Carlo (MCMC) basedapproaches [44]. Post‐selection inference‐based approaches could serve as viable alternatives [76, 77] in this scenario and remain areas for future research.

## Funding

The authors have nothing to report.

## Conflicts of Interest

The authors declare no conflicts of interest.

## Supporting information




**Data S1. Table S1:** Average true positive rate (TP), false positive rate (FP) for fixed (TPF, FPF) and random (TPR, FPR) effects, Scenario B. **Table S2:** Mean integrated squared error (MISE) of the functional fixed effect slope estimates from the MuFuMES method, scenario A. **Table S3:** Average true positive rate (TP), false positive rate (FP) for fixed (TPF, FPF) and random (TPR, FPR) effects, high‐dimensional scenario, p=100 functional fixed effects. **Table S4:** Mean integrated squared error (MISE) of the functional fixed effect slope estimates from the MuFuMES method, high‐dimensional scenario. **Table S5:** List of Predictors and their descriptive statistics (mean and standard de‐ viation for continuous variables and percentages for the categorical variables) in the NHANES 2011‐12 study (N=3402). The races MA, OH, NHW, NHB, NHA, OR re‐ fer to the races Mexican American, Other Hispanic, Non‐Hispanic White, Non‐Hispanic Black, Non‐Hispanic Asian, and Other Races, respectively. **Figure S1:** Displayed are the true (solid) and M.C mean (dashed) of estimated functional slopes $\beta_{k}(s)(k=2,\ldots ,5)$ from MuFuMES, n=100, Scenario B. **Figure S2:** Displayed are the true (solid) and M.C mean (dashed) of estimated functional slopes $\beta_{k}(s)(k=2,\ldots ,5)$ from MuFuMES, n = 25, high‐dimensional scenario. **Figure S3:** Predicted functional trajectories (${\hat{\beta }}_{1}(s)+{\hat{\beta }}_{1i}(s)$) of diurnal MIMS for males from the MuFuMES method in the NHANES application. The other continuous covariates were held at their average values. The solid line corresponds to the fixed effect prediction ${\hat{\beta }}_{1}(s)$ and the dashed line corresponds to the mixed effects prediction ${\hat{\beta }}_{1}(s)+{\hat{\beta }}_{1i}(s)$. The age‐by‐race groups are indicated in the header. **Figure S4:** Predicted functional trajectories (${\hat{\beta }}_{1}(s)+{\hat{\beta }}_{1i}(s)$) of diurnal MIMS for males from the MuFuMES method in the NHANES application. The other continuous covariates were held at their average values. The solid line corresponds to the fixed effect prediction ${\hat{\beta }}_{1}(s)$ and the dashed line corresponds to the mixed effects prediction ${\hat{\beta }}_{1}(s)+{\hat{\beta }}_{1i}(s)$. The age‐by‐race groups are indicated in the header. **Figure S5:** Predicted functional trajectories (${\hat{\beta }}_{1}(s)+{\hat{\beta }}_{1i}(s)$) of diurnal MIMS for males from the MuFuMES method in the NHANES application. The other continuous covariates were held at their average values. The solid line corresponds to the fixed effect prediction ${\hat{\beta }}_{1}(s)$ and the dashed line corresponds to the mixed effects prediction ${\hat{\beta }}_{1}(s)+{\hat{\beta }}_{1i}(s)$. The age‐by‐race groups are indicated in the header. **Figure S6:** Predicted functional trajectories (${\hat{\beta }}_{1}(s)+{\hat{\beta }}_{1i}(s)$) of diurnal MIMS for males from the MuFuMES method in the NHANES application. The other continuous covariates were held at their average values. The solid line corresponds to the fixed effect prediction ${\hat{\beta }}_{1}(s)$ and the dashed line corresponds to the mixed effects prediction ${\hat{\beta }}_{1}(s)+{\hat{\beta }}_{1i}(s)$. The age‐by‐race groups are indicated in the header. **Figure S7:** Predicted functional trajectories (${\hat{\beta }}_{1}(s)+{\hat{\beta }}_{1i}(s)$) of diurnal MIMS for males from the MuFuMES method in the NHANES application. The other continuous covariates were held at their average values. The solid line corresponds to the fixed effect prediction ${\hat{\beta }}_{1}(s)$ and the dashed line corresponds to the mixed effects prediction ${\hat{\beta }}_{1}(s)+{\hat{\beta }}_{1i}(s)$. The age‐by‐race groups are indicated in the header. **Figure S8:** Predicted functional trajectories (${\hat{\beta }}_{1}(s)+{\hat{\beta }}_{1i}(s)$) of diurnal MIMS for males from the MuFuMES method in the NHANES application. The other continuous covariates were held at their average values. The solid line corresponds to the fixed effect prediction ${\hat{\beta }}_{1}(s)$ and the dashed line corresponds to the mixed effects prediction ${\hat{\beta }}_{1}(s)+{\hat{\beta }}_{1i}(s)$. The age‐by‐race groups are indicated in the header. **Figure S9:** Predicted functional trajectories (${\hat{\beta }}_{1}(s)+{\hat{\beta }}_{1i}(s)$) of diurnal MIMS for males from the MuFuMES method in the NHANES application. The other continuous covariates were held at their average values. The solid line corresponds to the fixed effect prediction ${\hat{\beta }}_{1}(s)$ and the dashed line corresponds to the mixed effects prediction ${\hat{\beta }}_{1}(s)+{\hat{\beta }}_{1i}(s)$. The age‐by‐race groups are indicated in the header. **Figure S10:** Predicted functional trajectories (${\hat{\beta }}_{1}(s)+{\hat{\beta }}_{1i}(s)$) of diurnal MIMS for males from the MuFuMES method in the NHANES application. The other continuous covariates were held at their average values. The solid line corresponds to the fixed effect prediction ${\hat{\beta }}_{1}(s)$ and the dashed line corresponds to the mixed effects prediction ${\hat{\beta }}_{1}(s)+{\hat{\beta }}_{1i}(s)$. The age‐by‐race groups are indicated in the header. **Figure S11:** Predicted functional trajectories (${\hat{\beta }}_{1}(s)+{\hat{\beta }}_{1i}(s)$) of diurnal MIMS for males from the MuFuMES method in the NHANES application. The other continuous covariates were held at their average values. The solid line corresponds to the fixed effect prediction ${\hat{\beta }}_{1}(s)$ and the dashed line corresponds to the mixed effects prediction ${\hat{\beta }}_{1}(s)+{\hat{\beta }}_{1i}(s)$. The age‐by‐race groups are indicated in the header. **Figure S12:** Predicted functional trajectories (${\hat{\beta }}_{1}(s)+{\hat{\beta }}_{1i}(s)$) of diurnal MIMS for males from the MuFuMES method in the NHANES application. The other continuous covariates were held at their average values. The solid line corresponds to the fixed effect prediction ${\hat{\beta }}_{1}(s)$ and the dashed line corresponds to the mixed effects prediction ${\hat{\beta }}_{1}(s)+{\hat{\beta }}_{1i}(s)$. The age‐by‐race groups are indicated in the header. **Figure S13:** Predicted functional trajectories (${\hat{\beta }}_{1}(s)+{\hat{\beta }}_{1i}(s)$) of diurnal MIMS for males from the MuFuMES method in the NHANES application. The other continuous covariates were held at their average values. The solid line corresponds to the fixed effect prediction ${\hat{\beta }}_{1}(s)$ and the dashed line corresponds to the mixed effects prediction ${\hat{\beta }}_{1}(s)+{\hat{\beta }}_{1i}(s)$. The age‐by‐race groups are indicated in the header. **Figure S14:** Predicted functional trajectories (${\hat{\beta }}_{1}(s)+{\hat{\beta }}_{1i}(s)$) of diurnal MIMS for males from the MuFuMES method in the NHANES application. The other continuous covariates were held at their average values. The solid line corresponds to the fixed effect prediction ${\hat{\beta }}_{1}(s)$ and the dashed line corresponds to the mixed effects prediction ${\hat{\beta }}_{1}(s)+{\hat{\beta }}_{1i}(s)$. The age‐by‐race groups are indicated in the header. **Figure S15:** Predicted functional trajectories (${\hat{\beta }}_{1}(s)+{\hat{\beta }}_{1i}(s)$) of diurnal MIMS for males from the MuFuMES method in the NHANES application. The other continuous covariates were held at their average values. The solid line corresponds to the fixed effect prediction ${\hat{\beta }}_{1}(s)$ and the dashed line corresponds to the mixed effects prediction ${\hat{\beta }}_{1}(s)+{\hat{\beta }}_{1i}(s)$. The age‐by‐race groups are indicated in the header. **Figure S16:** Predicted functional trajectories (${\hat{\beta }}_{1}(s)+{\hat{\beta }}_{1i}(s)$) of diurnal MIMS for males from the MuFuMES method in the NHANES application. The other continuous covariates were held at their average values. The solid line corresponds to the fixed effect prediction ${\hat{\beta }}_{1}(s)$ and the dashed line corresponds to the mixed effects prediction ${\hat{\beta }}_{1}(s)+{\hat{\beta }}_{1i}(s)$. The age‐by‐race groups are indicated in the header. **Figure S17:** Predicted functional trajectories (${\hat{\beta }}_{1}(s)+{\hat{\beta }}_{1i}(s)$) of diurnal MIMS for males from the MuFuMES method in the NHANES application. The other continuous covariates were held at their average values. The solid line corresponds to the fixed effect prediction ${\hat{\beta }}_{1}(s)$ and the dashed line corresponds to the mixed effects prediction ${\hat{\beta }}_{1}(s)+{\hat{\beta }}_{1i}(s)$. The age‐by‐race groups are indicated in the header. **Figure S18:** Predicted functional trajectories (${\hat{\beta }}_{1}(s)+{\hat{\beta }}_{1i}(s)$) of diurnal MIMS for males from the MuFuMES method in the NHANES application. The other continuous covariates were held at their average values. The solid line corresponds to the fixed effect prediction ${\hat{\beta }}_{1}(s)$ and the dashed line corresponds to the mixed effects prediction ${\hat{\beta }}_{1}(s)+{\hat{\beta }}_{1i}(s)$. The age‐by‐race groups are indicated in the header. **Figure S19:** Predicted functional trajectories (${\hat{\beta }}_{1}(s)+{\hat{\beta }}_{1i}(s)$) of diurnal MIMS for males from the MuFuMES method in the NHANES application. The other continuous covariates were held at their average values. The solid line corresponds to the fixed effect prediction ${\hat{\beta }}_{1}(s)$ and the dashed line corresponds to the mixed effects prediction ${\hat{\beta }}_{1}(s)+{\hat{\beta }}_{1i}(s)$. The age‐by‐race groups are indicated in the header. **Figure S20:** Predicted functional trajectories (${\hat{\beta }}_{1}(s)+{\hat{\beta }}_{1i}(s)$) of diurnal MIMS for males from the MuFuMES method in the NHANES application. The other continuous covariates were held at their average values. The solid line corresponds to the fixed effect prediction ${\hat{\beta }}_{1}(s)$ and the dashed line corresponds to the mixed effects prediction ${\hat{\beta }}_{1}(s)+{\hat{\beta }}_{1i}(s)$. The age‐by‐race groups are indicated in the header. **Figure S21:** Predicted functional trajectories (${\hat{\beta }}_{1}(s)+{\hat{\beta }}_{1i}(s)$) of diurnal MIMS for males from the MuFuMES method in the NHANES application. The other continuous covariates were held at their average values. The solid line corresponds to the fixed effect prediction ${\hat{\beta }}_{1}(s)$ and the dashed line corresponds to the mixed effects prediction ${\hat{\beta }}_{1}(s)+{\hat{\beta }}_{1i}(s)$. The age‐by‐race groups are indicated in the header. **Figure S22:** Predicted functional trajectories (${\hat{\beta }}_{1}(s)+{\hat{\beta }}_{1i}(s)$) of diurnal MIMS for males from the MuFuMES method in the NHANES application. The other continuous covariates were held at their average values. The solid line corresponds to the fixed effect prediction ${\hat{\beta }}_{1}(s)$ and the dashed line corresponds to the mixed effects prediction ${\hat{\beta }}_{1}(s)+{\hat{\beta }}_{1i}(s)$. The age‐by‐race groups are indicated in the header. **Figure S23:** Predicted functional trajectories (${\hat{\beta }}_{1}(s)+{\hat{\beta }}_{1i}(s)$) of diurnal MIMS for males from the MuFuMES method in the NHANES application. The other continuous covariates were held at their average values. The solid line corresponds to the fixed effect prediction ${\hat{\beta }}_{1}(s)$ and the dashed line corresponds to the mixed effects prediction ${\hat{\beta }}_{1}(s)+{\hat{\beta }}_{1i}(s)$. The age‐by‐race groups are indicated in the header. **Figure S24:** Predicted functional trajectories (${\hat{\beta }}_{1}(s)+{\hat{\beta }}_{1i}(s)$) of diurnal MIMS for males from the MuFuMES method in the NHANES application. The other continuous covariates were held at their average values. The solid line corresponds to the fixed effect prediction ${\hat{\beta }}_{1}(s)$ and the dashed line corresponds to the mixed effects prediction ${\hat{\beta }}_{1}(s)+{\hat{\beta }}_{1i}(s)$. The age‐by‐race groups are indicated in the header. **Figure S25:** Predicted functional trajectories (${\hat{\beta }}_{1}(s)+{\hat{\beta }}_{1i}(s)$) of diurnal MIMS for males from the MuFuMES method in the NHANES application. The other continuous covariates were held at their average values. The solid line corresponds to the fixed effect prediction ${\hat{\beta }}_{1}(s)$ and the dashed line corresponds to the mixed effects prediction ${\hat{\beta }}_{1}(s)+{\hat{\beta }}_{1i}(s)$. The age‐by‐race groups are indicated in the header. **Figure S26:** Predicted functional trajectories (${\hat{\beta }}_{1}(s)+{\hat{\beta }}_{1i}(s)$) of diurnal MIMS for males from the MuFuMES method in the NHANES application. The other continuous covariates were held at their average values. The solid line corresponds to the fixed effect prediction ${\hat{\beta }}_{1}(s)$ and the dashed line corresponds to the mixed effects prediction ${\hat{\beta }}_{1}(s)+{\hat{\beta }}_{1i}(s)$. The age‐by‐race groups are indicated in the header. **Figure S27:** Predicted functional trajectories (${\hat{\beta }}_{1}(s)+{\hat{\beta }}_{1i}(s)$) of diurnal MIMS for males from the MuFuMES method in the NHANES application. The other continuous covariates were held at their average values. The solid line corresponds to the fixed effect prediction ${\hat{\beta }}_{1}(s)$ and the dashed line corresponds to the mixed effects prediction ${\hat{\beta }}_{1}(s)+{\hat{\beta }}_{1i}(s)$. The age‐by‐race groups are indicated in the header. **Figure S28:** Predicted functional trajectories (${\hat{\beta }}_{1}(s)+{\hat{\beta }}_{1i}(s)$) of diurnal MIMS for males from the MuFuMES method in the NHANES application. The other continuous covariates were held at their average values. The solid line corresponds to the fixed effect prediction ${\hat{\beta }}_{1}(s)$ and the dashed line corresponds to the mixed effects prediction ${\hat{\beta }}_{1}(s)+{\hat{\beta }}_{1i}(s)$. The age‐by‐race groups are indicated in the header. **Figure S29:** Predicted functional trajectories (${\hat{\beta }}_{1}(s)+{\hat{\beta }}_{1i}(s)$) of diurnal MIMS for males from the MuFuMES method in the NHANES application. The other continuous covariates were held at their average values. The solid line corresponds to the fixed effect prediction ${\hat{\beta }}_{1}(s)$ and the dashed line corresponds to the mixed effects prediction ${\hat{\beta }}_{1}(s)+{\hat{\beta }}_{1i}(s)$. The age‐by‐race groups are indicated in the header. **Figure S30:** Predicted functional trajectories (${\hat{\beta }}_{1}(s)+{\hat{\beta }}_{1i}(s)$) of diurnal MIMS for males from the MuFuMES method in the NHANES application. The other continuous covariates were held at their average values. The solid line corresponds to the fixed effect prediction ${\hat{\beta }}_{1}(s)$ and the dashed line corresponds to the mixed effects prediction ${\hat{\beta }}_{1}(s)+{\hat{\beta }}_{1i}(s)$. The age‐by‐race groups are indicated in the header. **Figure S31:** Predicted functional trajectories (${\hat{\beta }}_{1}(s)+{\hat{\beta }}_{1i}(s)$) of diurnal MIMS for males from the MuFuMES method in the NHANES application. The other continuous covariates were held at their average values. The solid line corresponds to the fixed effect prediction ${\hat{\beta }}_{1}(s)$ and the dashed line corresponds to the mixed effects prediction ${\hat{\beta }}_{1}(s)+{\hat{\beta }}_{1i}(s)$. The age‐by‐race groups are indicated in the header. **Figure S32:** Predicted functional trajectories (${\hat{\beta }}_{1}(s)+{\hat{\beta }}_{1i}(s)$) of diurnal MIMS for males from the MuFuMES method in the NHANES application. The other continuous covariates were held at their average values. The solid line corresponds to the fixed effect prediction ${\hat{\beta }}_{1}(s)$ and the dashed line corresponds to the mixed effects prediction ${\hat{\beta }}_{1}(s)+{\hat{\beta }}_{1i}(s)$. The age‐by‐race groups are indicated in the header. **Figure S33:** Predicted functional trajectories (${\hat{\beta }}_{1}(s)+{\hat{\beta }}_{1i}(s)$) of diurnal MIMS for males from the MuFuMES method in the NHANES application. The other continuous covariates were held at their average values. The solid line corresponds to the fixed effect prediction ${\hat{\beta }}_{1}(s)$ and the dashed line corresponds to the mixed effects prediction ${\hat{\beta }}_{1}(s)+{\hat{\beta }}_{1i}(s)$. The age‐by‐race groups are indicated in the header. **Figure S34:** Predicted functional trajectories (${\hat{\beta }}_{1}(s)+{\hat{\beta }}_{1i}(s)$) of diurnal MIMS for males from the MuFuMES method in the NHANES application. The other continuous covariates were held at their average values. The solid line corresponds to the fixed effect prediction ${\hat{\beta }}_{1}(s)$ and the dashed line corresponds to the mixed effects prediction ${\hat{\beta }}_{1}(s)+{\hat{\beta }}_{1i}(s)$. The age‐by‐race groups are indicated in the header. **Figure S35:** Predicted functional trajectories (${\hat{\beta }}_{1}(s)+{\hat{\beta }}_{1i}(s)$) of diurnal MIMS for males from the MuFuMES method in the NHANES application. The other continuous covariates were held at their average values. The solid line corresponds to the fixed effect prediction ${\hat{\beta }}_{1}(s)$ and the dashed line corresponds to the mixed effects prediction ${\hat{\beta }}_{1}(s)+{\hat{\beta }}_{1i}(s)$. The age‐by‐race groups are indicated in the header. **Figure S36:** Predicted functional trajectories (${\hat{\beta }}_{1}(s)+{\hat{\beta }}_{1i}(s)$) of diurnal MIMS for males from the MuFuMES method in the NHANES application. The other continuous covariates were held at their average values. The solid line corresponds to the fixed effect prediction ${\hat{\beta }}_{1}(s)$ and the dashed line corresponds to the mixed effects prediction ${\hat{\beta }}_{1}(s)+{\hat{\beta }}_{1i}(s)$. The age‐by‐race groups are indicated in the header. **Figure S37:** Predicted functional trajectories (${\hat{\beta }}_{1}(s)+{\hat{\beta }}_{1i}(s)$) of diurnal MIMS for males from the MuFuMES method in the NHANES application. The other continuous covariates were held at their average values. The solid line corresponds to the fixed effect prediction ${\hat{\beta }}_{1}(s)$ and the dashed line corresponds to the mixed effects prediction ${\hat{\beta }}_{1}(s)+{\hat{\beta }}_{1i}(s)$. The age‐by‐race groups are indicated in the header. **Figure S38:** Predicted functional trajectories (${\hat{\beta }}_{1}(s)+{\hat{\beta }}_{1i}(s)$) of diurnal MIMS for males from the MuFuMES method in the NHANES application. The other continuous covariates were held at their average values. The solid line corresponds to the fixed effect prediction ${\hat{\beta }}_{1}(s)$ and the dashed line corresponds to the mixed effects prediction ${\hat{\beta }}_{1}(s)+{\hat{\beta }}_{1i}(s)$. The age‐by‐race groups are indicated in the header.

## Data Availability

Software implementation via R [78] and illustration of the proposed framework are included with this article and will be made available on GitHub. The data that support the findings of this study are openly available in CDC website at: https://wwwn.cdc.gov/nchs/nhanes/continuousnhanes/default.aspx.
