# Supplementary material for: Variable Selection for Fixed and Random Effects in Multilevel Functional Mixed Effects Models
Source: Stat Med. 2026 Aug 9;45(18-19):e70694. doi: 10.1002/sim.70694 (PMC13454179; doi:10.1002/sim.70694)
Supplement: Supplementary file 1 [file SIM-45-0-s001.zip › Supplementary Material.pdf]

Supplementary Material for Variable Selection for Fixed and Random Effects in Multilevel Functional Mixed Effects Models

March 31, 2026

1 Appendix A: Additional Simulations

Scenario B

We generate observations following a multilevel functional mixed effects model given by,

$$Y_{ij}(s) = \sum_{k=1}^{11} X_{ijk}\beta_k(s) + \sum_{r=1}^{8} Z_{ijr}u_{ir}(s) + v_{ij}(s) + \epsilon_{ij}(s), s \in [0,1], \tag{1}$$

for clusters $i = 1, \ldots, n$ and replications $j = 1, \ldots, J_i$. In this model, we consider $p = 11$ covariates for functional fixed effects (including a fixed intercept) and $q = 8$ covariates for functional random effects (including a cluster-level random intercept). We denote by $\phi(s, a, b^2)$ the density at s for Normal distribution with mean a and variance b^2. The fixed effects coefficient functions are given by $\beta_1(s) = 8sin(2\pi s)$ (intercept), $\beta_2(s) = 2\phi(s, 0.6, 0.15^2)$, $\beta_3(s) = 2.5\phi(s, 0.6, 0.15^2)$, $\beta_4(s) = 3cos(2\pi s)$, $\beta_5(s) = 5sin(2\pi s) + 5cos(2\pi s)$ and $\beta_k(s) = 0$ for $k = 6, 7, \ldots, 11$. So, only the first 5 fixed effect covariates are relevant. The fixed effect covariates X_{ijk} are independently generated from a $\mathcal{N}(0, 2^2)$ distribution for $k = 2, \ldots, 11$ and $X_{ij1} = 1$ (for all i, j) corresponds to the intercept. The cluster-level functional random effects are given by $u_{i1}(s) = c_{i1}sin(2\pi s) + d_{i1}cos(2\pi s)$ (random intercept), where $c_{i1} \sim \mathcal{N}(0, 3^2\sigma_B^2), d_{i1} \sim \mathcal{N}(0, 1.5^2\sigma_B^2)$. Similarly, $u_{i4}(s) = c_{i4}sin(2\pi s) + d_{i4}cos(2\pi s) + e_{i4}sin(\pi s) + f_{i4}cos(\pi s)$, where $c_{i4} \sim \mathcal{N}(0, 1.5^2\sigma_B^2), d_{i4} \sim \mathcal{N}(0, 0.75^2\sigma_B^2), e_{i4} \sim \mathcal{N}(0, 0.5^2\sigma_B^2), f_{i4} \sim \mathcal{N}(0, 0.25^2\sigma_B^2)$. The rest of the functional random effects $u_{ik}(s)$ are considered to be zero. Hence only the first and the fourth random effect of the covariates are important. In this scenario, Z_{ijr} are independently generated from a $\mathcal{N}(0, 2^2)$ distribution for $r = 2, \ldots, 8$ and $Z_{ij1} = 1$ (for all i, j) corresponds to the random intercept term. We set σ_B^2 based on $SNR_B = 0.5$, where SNR_B is the standard deviation of the fixed effects functions divided by the standard deviation of the random effects (Cui et al., 2022; Scheipl et al., 2015). The cluster-subject-level functional random effect $v_{ij}(s)$ is given by $v_{ij}(s) = v_{ij1}sin(\pi s) + v_{ij2}cos(\pi s)$, where $v_{ij1} \sim \mathcal{N}(0, 0.8^2\sigma_S^2), v_{ij2} \sim \mathcal{N}(0, 0.4^2\sigma_S^2)$. We set σ_S^2 based on $SNR_S = 2$, where SNR_S is the standard deviation of the fixed effects functions divided by the standard deviation of the cluster-visit level random effects. The random errors $\epsilon_{ij}(s) \sim \mathcal{N}(0, \sigma_\epsilon^2)$, where σ_ϵ is chosen based on a signal to noise ratio of $SNR_\epsilon = 4$, which represents the standard deviation of the linear predictors (fixed and random predictors combined) divided by the

standard deviation of the noise σ_ϵ. The functional response $Y_{ij}(s)$ is observed on a grid of $m = 10$ equidistant time points in $S = [0, 1]$. Cluster size $n \in \{25, 50, 100\}$ are considered for this scenario, and $J_i = J = 10$ replications are considered within each cluster.

Simulation Results: Scenario B

The performance of the proposed variable selection (MuFuMES) method is evaluated in terms of selection accuracy and estimation accuracy. The tuning parameters of the MuFuMES method are chosen based on the proposed BIC criterion (26). Table S3 reports the selection performance of MuFuMES for the fixed effects and random effects in terms of true positive and false positive rates for both the fixed and random functional effects.

Table S1: Average true positive rate (TP), false positive rate (FP) for fixed (TPF, FPF) and random (TPR, FPR) effects, Scenario B.

Sample Size	Method	TPF	FPF	TPR	FPR
n=25	MuFuMES	0.95	0.02	1	0.015
n=50	MuFuMES	0.99	0.003	1	0.003
n=100	MuFuMES	1	0.005	1	0.01

We observe that the proposed MuFuMES method has a negligible false positive rate and a high true positive rate for both fixed and random effects across all the sample sizes, illustrating parsimonious and accurate model selection. The performance can also be seen to improve with an increasing sample size. Next, we illustrate the estimation performance of the proposed MuFuMES method. We display the Monte Carlo (MC) mean estimates (averaged estimated coefficient function over 100 replications) of the functional fixed effect slopes $\beta_k(s)$ $(k = 2, 3, 4, 5)$ in Figure S2 for sample size $n = 100$. The estimated coefficient functions are superimposed on the true curves and can be seen to capture the true effects closely, indicating a satisfactory performance of the proposed method in terms of estimation. We also report the mean integrated squared error (MISE) of the functional fixed effect slope estimates in Table S4 across the three sample sizes. The MISE of the functional estimate $\hat{\beta}_k(s)$ is defined as:

$$MISE_k = \frac{1}{B}\sum_{b=1}^{B}\left(\int_0^1 \left(\hat{\beta}_{kb}(s) - \beta_k(s)\right)^2 ds\right),$$

where $\hat{\beta}_{kb}(s)$ represents the estimate of $\beta_k(s)$ for the b-th Monte-Carlo (M.C.) replication,

and B is the total number of replications.

Table S2: Mean integrated squared error (MISE) of the functional fixed effect slope estimates from the MuFuMES method, scenario A.

Sample Size	MISE $\beta_2(\cdot)$	MISE $\beta_3(\cdot)$	MISE $\beta_4(\cdot)$	MISE $\beta_5(\cdot)$
n=25	0.160	0.158	0.154	0.162
n=50	0.071	0.071	0.071	0.075
n=100	0.037	0.038	0.038	0.036

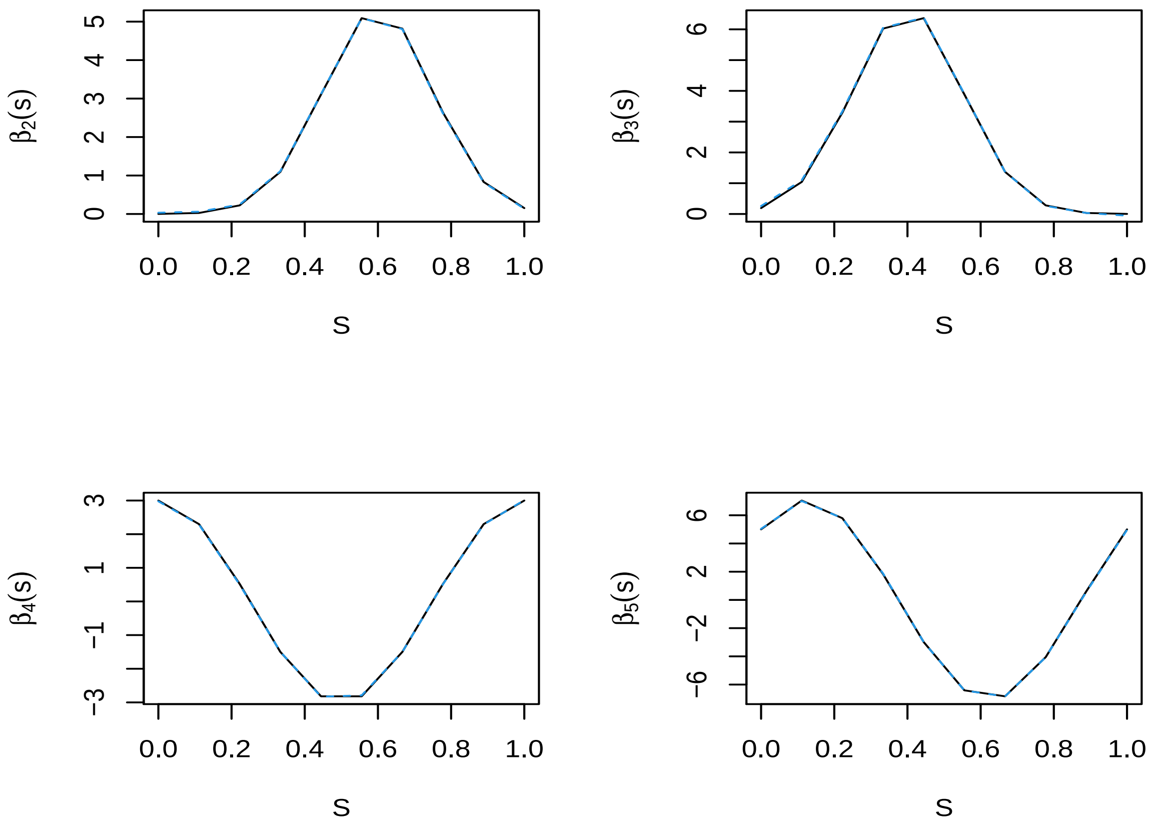

Figure S1: Displayed are the true (solid) and M.C mean (dashed) of estimated functional slopes $\beta_k(s)$ (k=2,...,5) from MuFuMES, $n = 100$, Scenario B.

The proposed MuFuMES method can be seen to estimate the true functional parameters accurately, with MISE decreasing with an increasing sample size, indicating the consistency of the estimators.

Scenario C: High-dimensional scenario

In this scenario, we generate observations following a multilevel functional mixed effects model given by,

$$Y_{ij}(s) = \sum_{k=1}^{100} X_{ijk}\beta_k(s) + \sum_{r=1}^{10} Z_{ijr}u_{ir}(s) + v_{ij}(s) + \epsilon_{ij}(s), s \in [0,1], \tag{2}$$

for clusters $i = 1, \ldots, n$ and replications $j = 1, \ldots, J_i$. In this high-dimensional scenario, we consider $p = 100$ covariates for functional fixed effects (including a fixed intercept) and $q = 10$ covariates for functional random effects (including a cluster-level random intercept). The rest of the simulation design is kept exactly the same as in Scenario B. Cluster size $n = 25$ is considered for this scenario, and $J_i = J = 10$ replications are considered within each cluster.

Table S3: Average true positive rate (TP), false positive rate (FP) for fixed (TPF, FPF) and random (TPR, FPR) effects, high-dimensional scenario, $p = 100$ functional fixed effects.

Sample Size	Method	TPF	FPF	TPR	FPR
n=25	MuFuMES	0.91	0.005	0.93	0

Table S4: Mean integrated squared error (MISE) of the functional fixed effect slope estimates from the MuFuMES method, high-dimensional scenario.

Sample Size	MISE $\beta_2(\cdot)$	MISE $\beta_3(\cdot)$	MISE $\beta_4(\cdot)$	MISE $\beta_5(\cdot)$
n=25	0.36	0.23	0.35	0.28

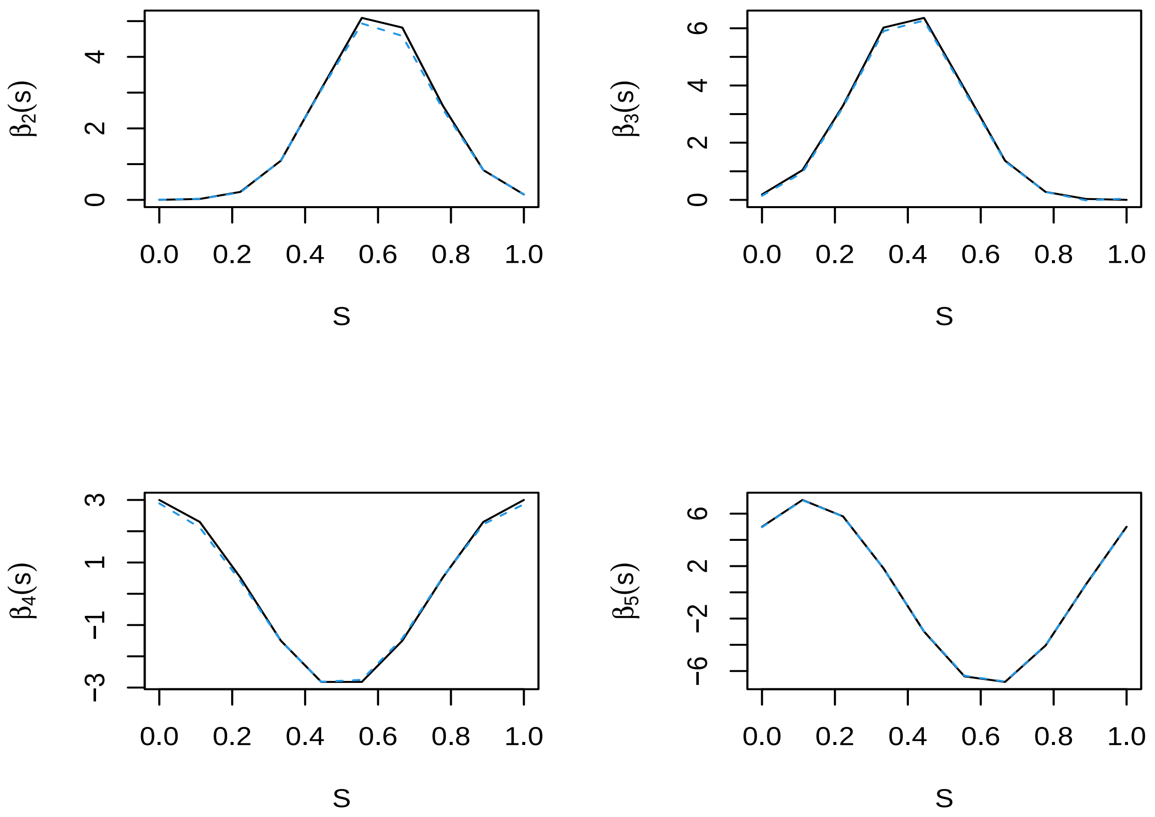

Figure S2: Displayed are the true (solid) and M.C mean (dashed) of estimated functional slopes $\beta_k(s)$ (k=2,...,5) from MuFuMES, $n = 25$, high-dimensional scneario.

The proposed MuFuMES method can be seen to estimate the true functional fixed effects accurately and have a high true positive rate (above 0.9) and a negligible false positive rate (below 0.01) for the functional fixed and random effects, even in this high-dimensional scenario.

2 Appendix B: Supplementary Tables

Table S5: List of Predictors and their descriptive statistics (mean and standard deviation for continuous variables and percentages for the categorical variables) in the NHANES 2011-12 study (N=3402). The races MA, OH, NHW, NHB, NHA, OR refer to the races Mexican American, Other Hispanic, Non-Hispanic White, Non-Hispanic Black, Non-Hispanic Asian, and Other Races, respectively.

Predictor	Description	mean (sd) / %
Gender (female)	Categorical	50.8
Age	Continuous	48.7 (17.6)
BMI	Body Mass Index (Kg/m^2)	29.16 (7)
Race (categorical)	MA, OH, NHW, NHB, NHA, OR	9.3, 9, 41.2, 25.9, 11.6, 2.9
INDFMPIR (Continuous)	Ratio of family income to poverty	2.5 (1.7)
MGDCGSZ (Continuous)	Combined grip strength (Kg)	71.0 (22.4)
HEI (continuous)	Healthy Eating Index	55.2 (13.3)

Predicted cluster trajectories

We display the predicted trajectories of the 36 clusters from the MuFuMES method for the male group, with the other covariates held at the average value. The interaction of six age groups ($20-30, 30-40, \ldots, 70-80$) and six races (MA=1, OH=2, NHW=3, NHB=4, NHA=6, OR=7) leading to the 36 age-by-race groups are indicated in the header of each of the following figures.

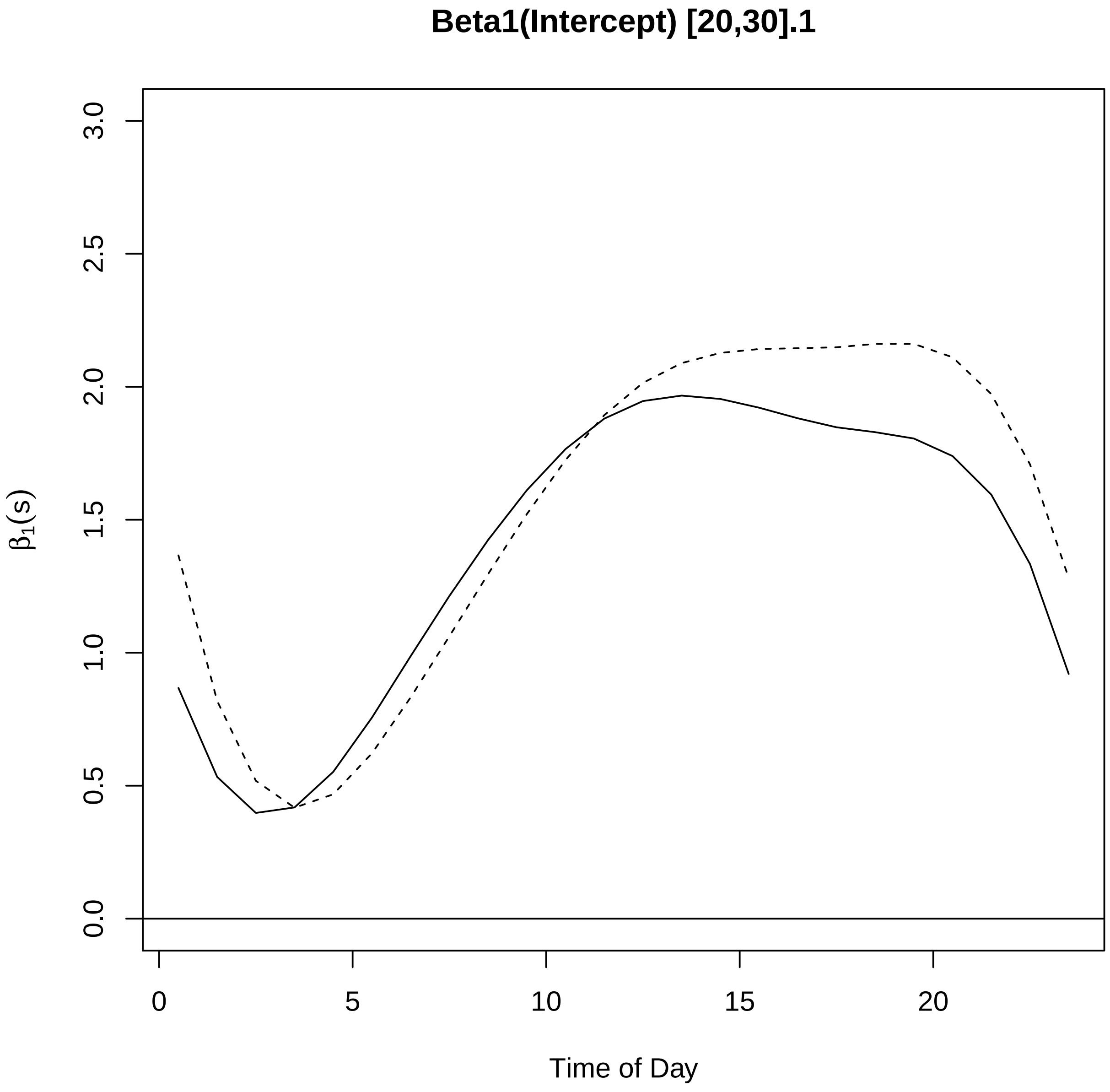

Figure S3: Predicted functional trajectories $(\hat{\beta}_1(s) + \hat{\beta}_{1i}(s))$ of diurnal MIMS for males from the MuFuMES method in the NHANES application. The other continuous covariates were held at their average values. The solid line corresponds to the fixed effect prediction $\hat{\beta}_1(s)$ and the dashed line corresponds to the mixed effects prediction $\hat{\beta}_1(s) + \hat{\beta}_{1i}(s)$. The age-by-race groups are indicated in the header.

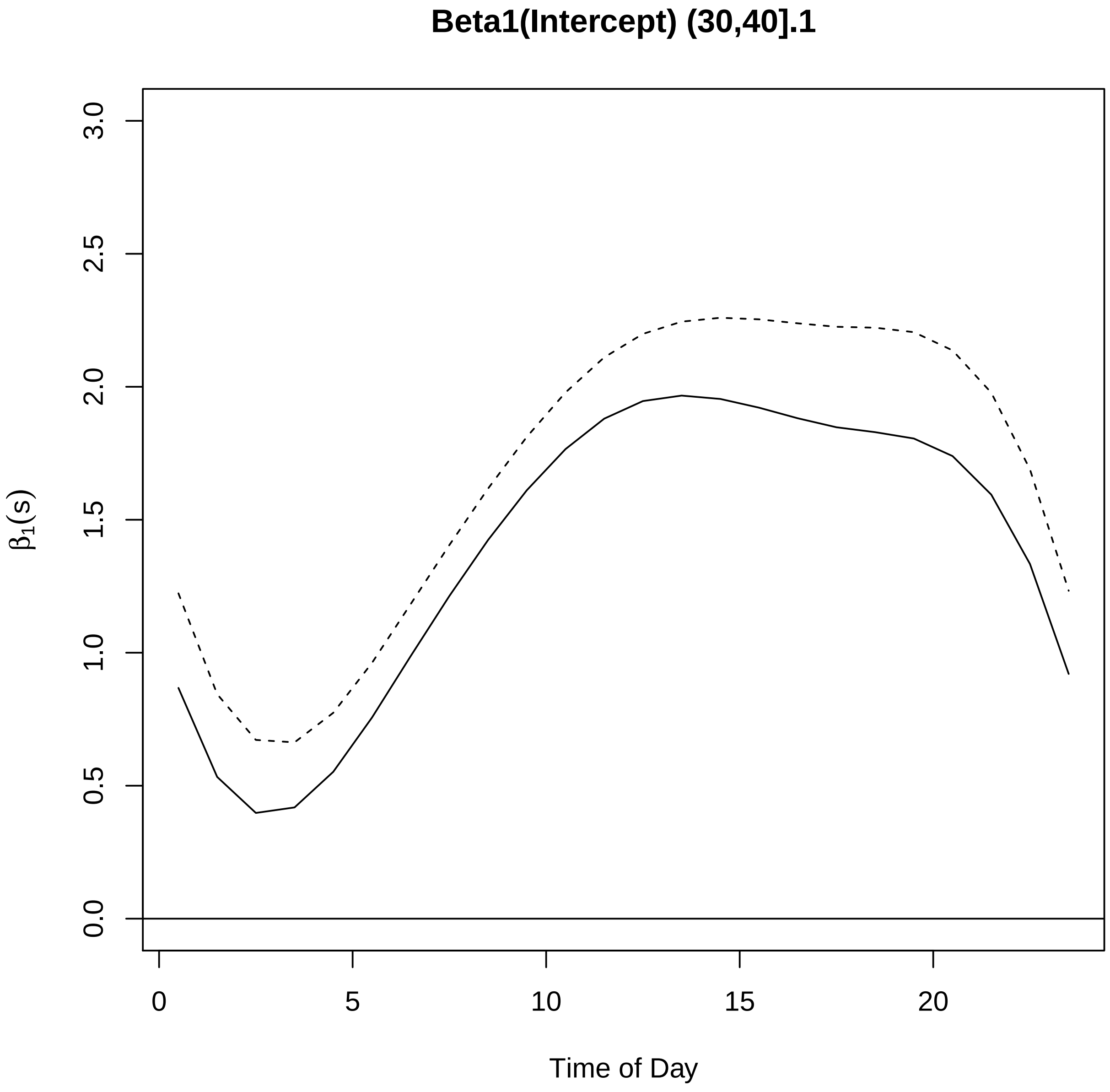

Figure S4: Predicted functional trajectories ($\hat{\beta}_1(s) + \hat{\beta}_{1i}(s)$) of diurnal MIMS for males from the MuFuMES method in the NHANES application. The other continuous covariates were held at their average values. The solid line corresponds to the fixed effect prediction $\hat{\beta}_1(s)$ and the dashed line corresponds to the mixed effects prediction $\hat{\beta}_1(s) + \hat{\beta}_{1i}(s)$. The age-by-race groups are indicated in the header.

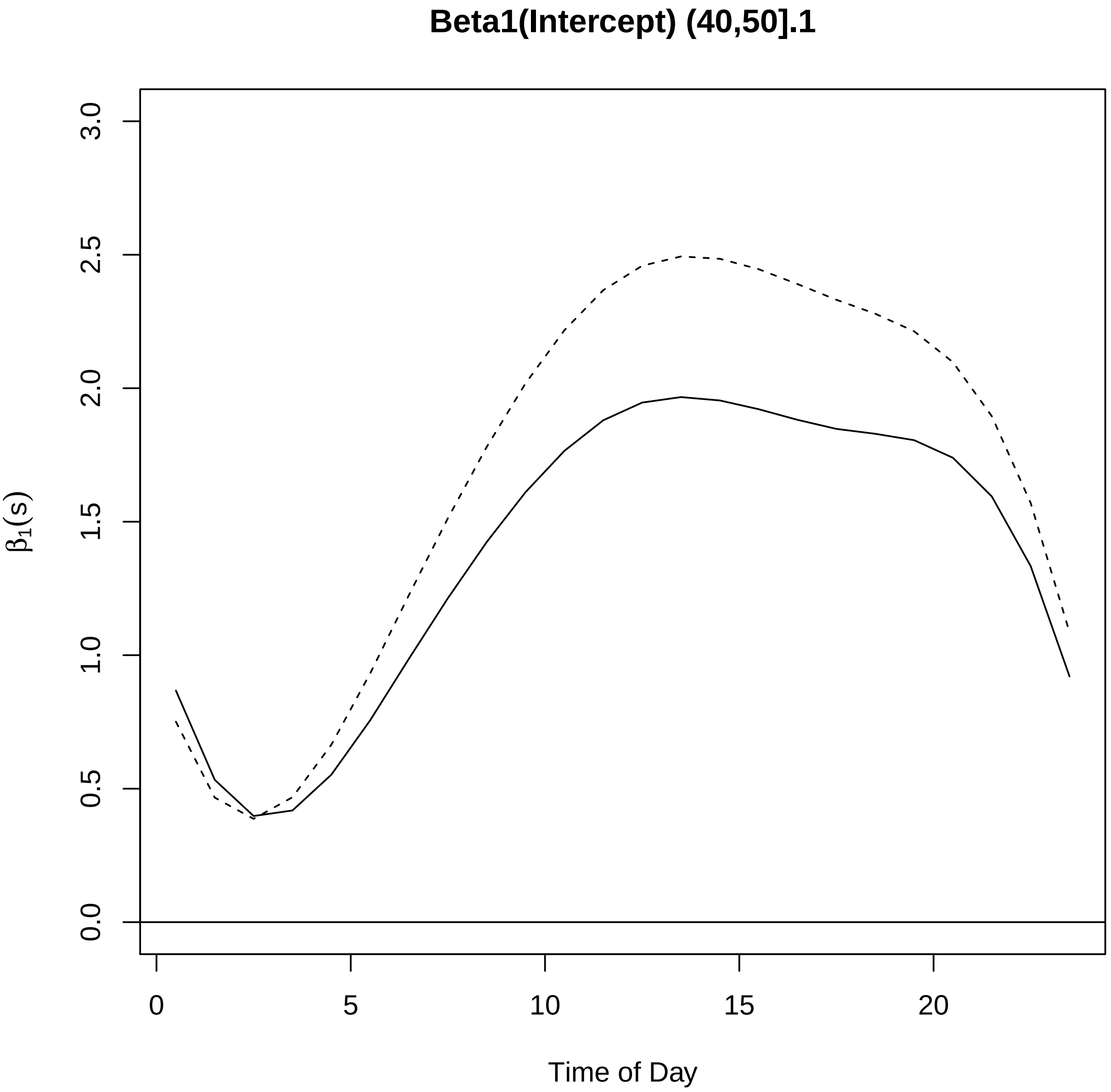

Figure S5: Predicted functional trajectories $(\hat{\beta}_1(s) + \hat{\beta}_{1i}(s))$ of diurnal MIMS for males from the MuFuMES method in the NHANES application. The other continuous covariates were held at their average values. The solid line corresponds to the fixed effect prediction $\hat{\beta}_1(s)$ and the dashed line corresponds to the mixed effects prediction $\hat{\beta}_1(s) + \hat{\beta}_{1i}(s)$. The age-by-race groups are indicated in the header.

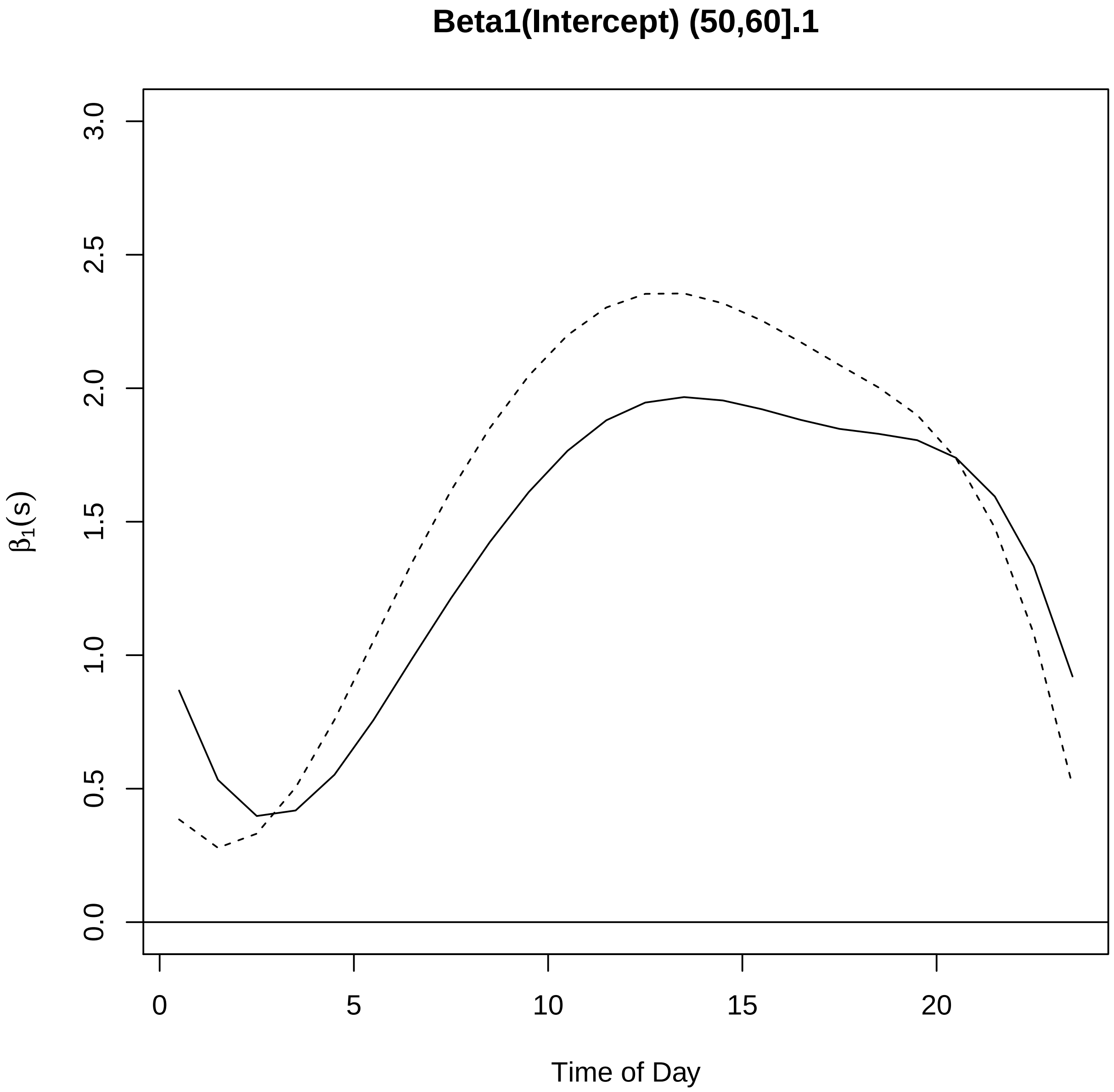

Figure S6: Predicted functional trajectories ($\hat{\beta}_1(s) + \hat{\beta}_{1i}(s)$) of diurnal MIMS for males from the MuFuMES method in the NHANES application. The other continuous covariates were held at their average values. The solid line corresponds to the fixed effect prediction $\hat{\beta}_1(s)$ and the dashed line corresponds to the mixed effects prediction $\hat{\beta}_1(s) + \hat{\beta}_{1i}(s)$. The age-by-race groups are indicated in the header.

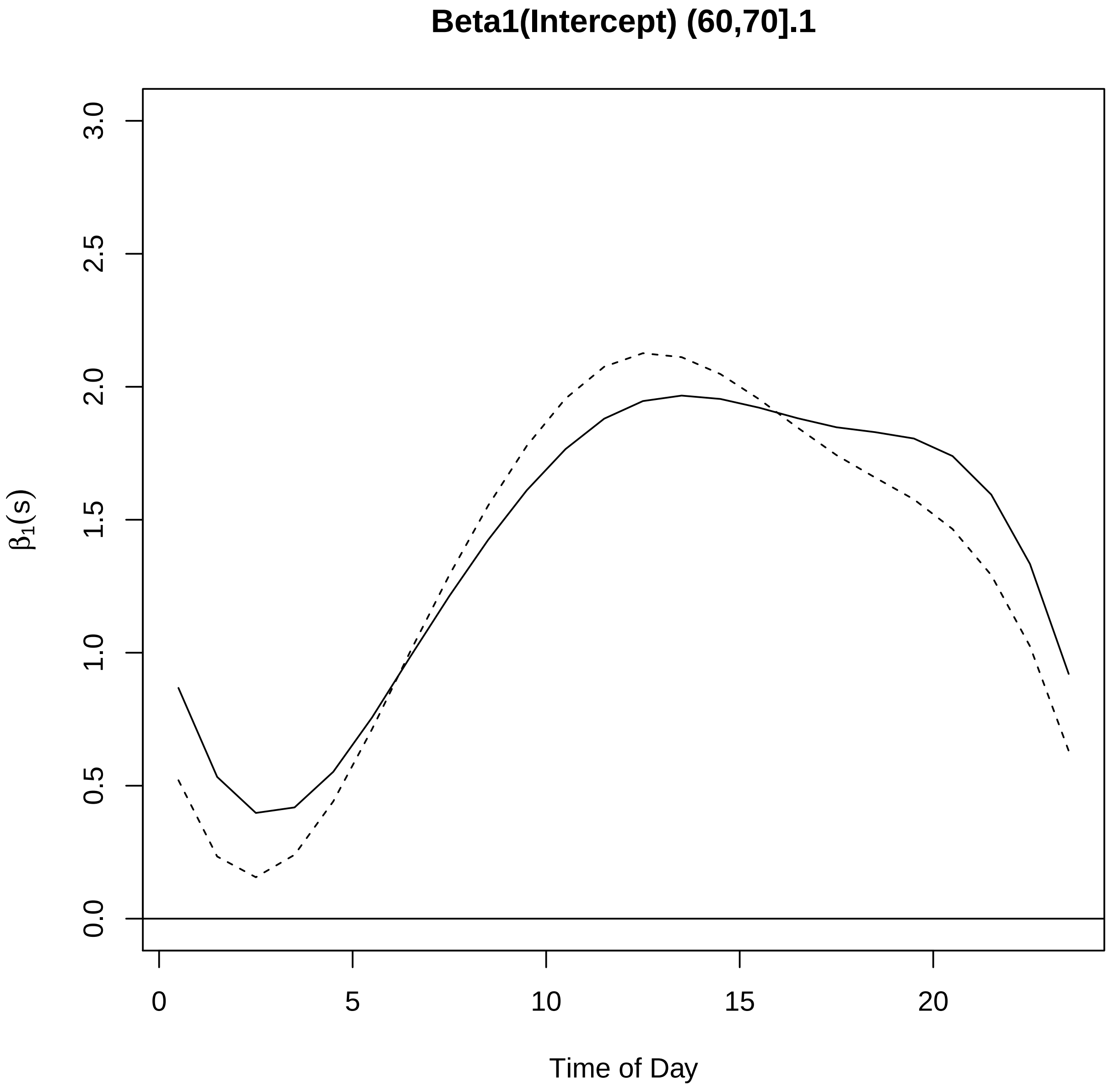

Figure S7: Predicted functional trajectories $(\hat{\beta}_1(s) + \hat{\beta}_{1i}(s))$ of diurnal MIMS for males from the MuFuMES method in the NHANES application. The other continuous covariates were held at their average values. The solid line corresponds to the fixed effect prediction $\hat{\beta}_1(s)$ and the dashed line corresponds to the mixed effects prediction $\hat{\beta}_1(s) + \hat{\beta}_{1i}(s)$. The age-by-race groups are indicated in the header.

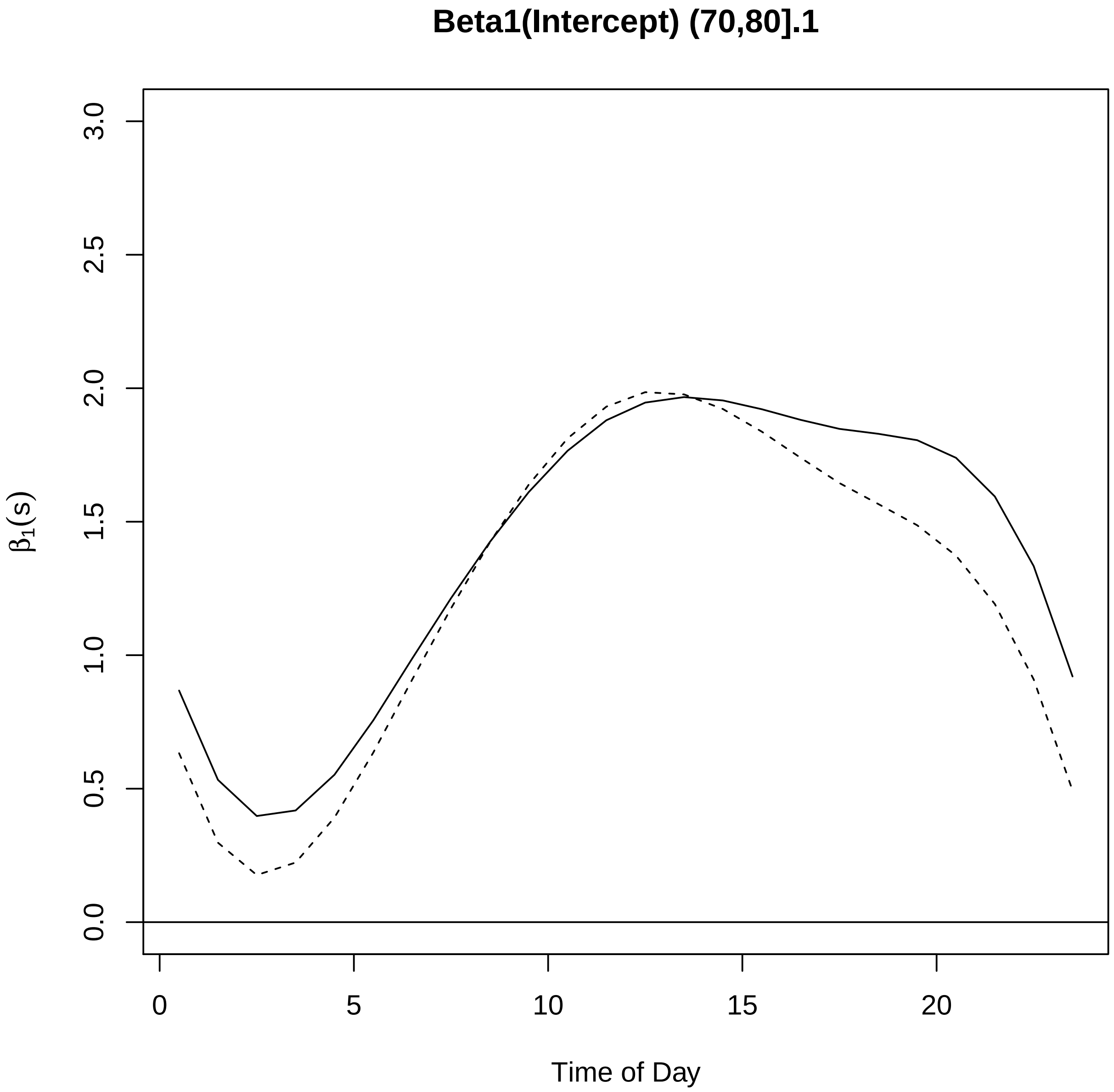

Figure S8: Predicted functional trajectories ($\hat{\beta}_1(s) + \hat{\beta}_{1i}(s)$) of diurnal MIMS for males from the MuFuMES method in the NHANES application. The other continuous covariates were held at their average values. The solid line corresponds to the fixed effect prediction $\hat{\beta}_1(s)$ and the dashed line corresponds to the mixed effects prediction $\hat{\beta}_1(s) + \hat{\beta}_{1i}(s)$. The age-by-race groups are indicated in the header.

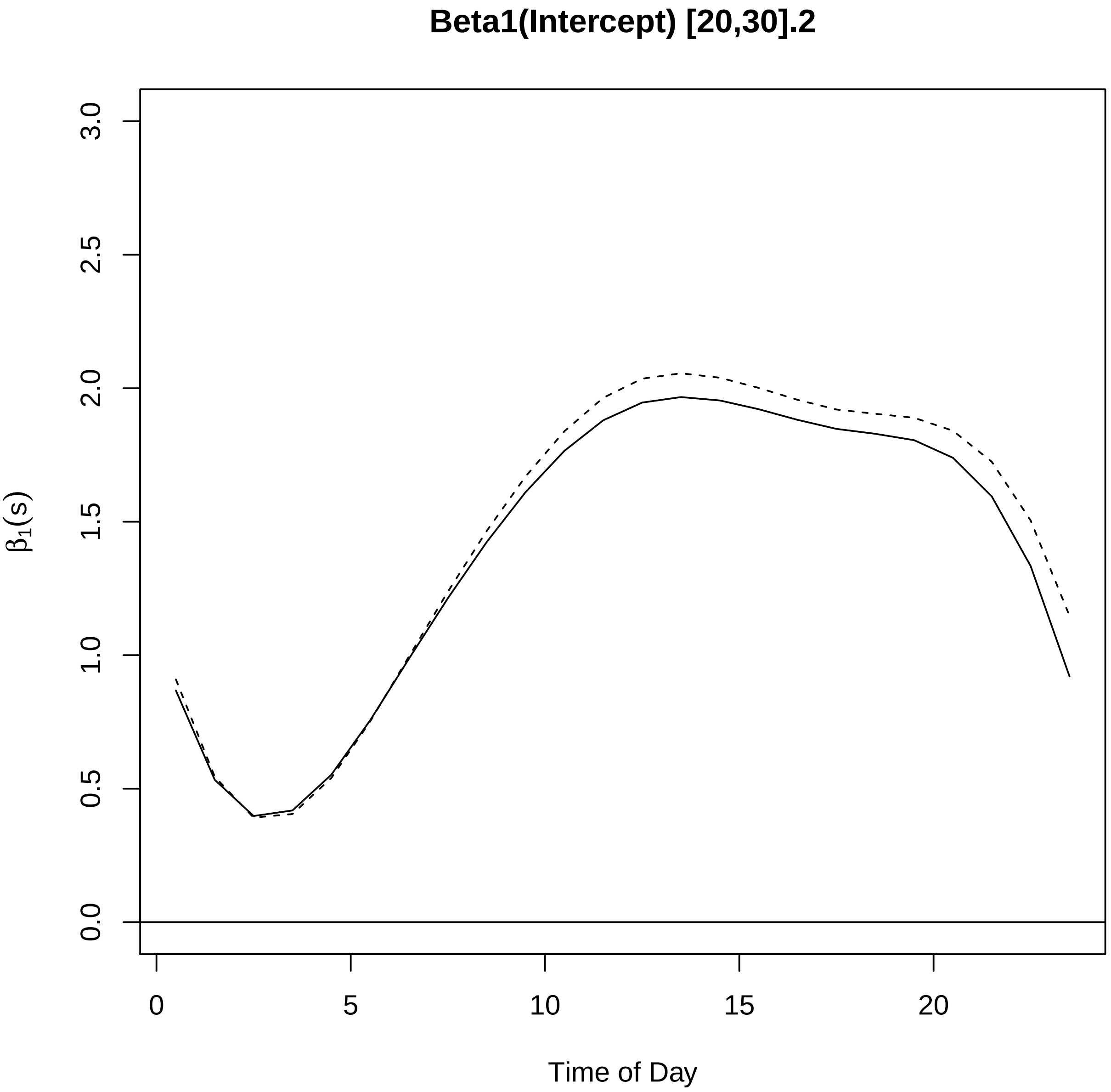

Figure S9: Predicted functional trajectories $(\hat{\beta}_1(s) + \hat{\beta}_{1i}(s))$ of diurnal MIMS for males from the MuFuMES method in the NHANES application. The other continuous covariates were held at their average values. The solid line corresponds to the fixed effect prediction $\hat{\beta}_1(s)$ and the dashed line corresponds to the mixed effects prediction $\hat{\beta}_1(s) + \hat{\beta}_{1i}(s)$. The age-by-race groups are indicated in the header.

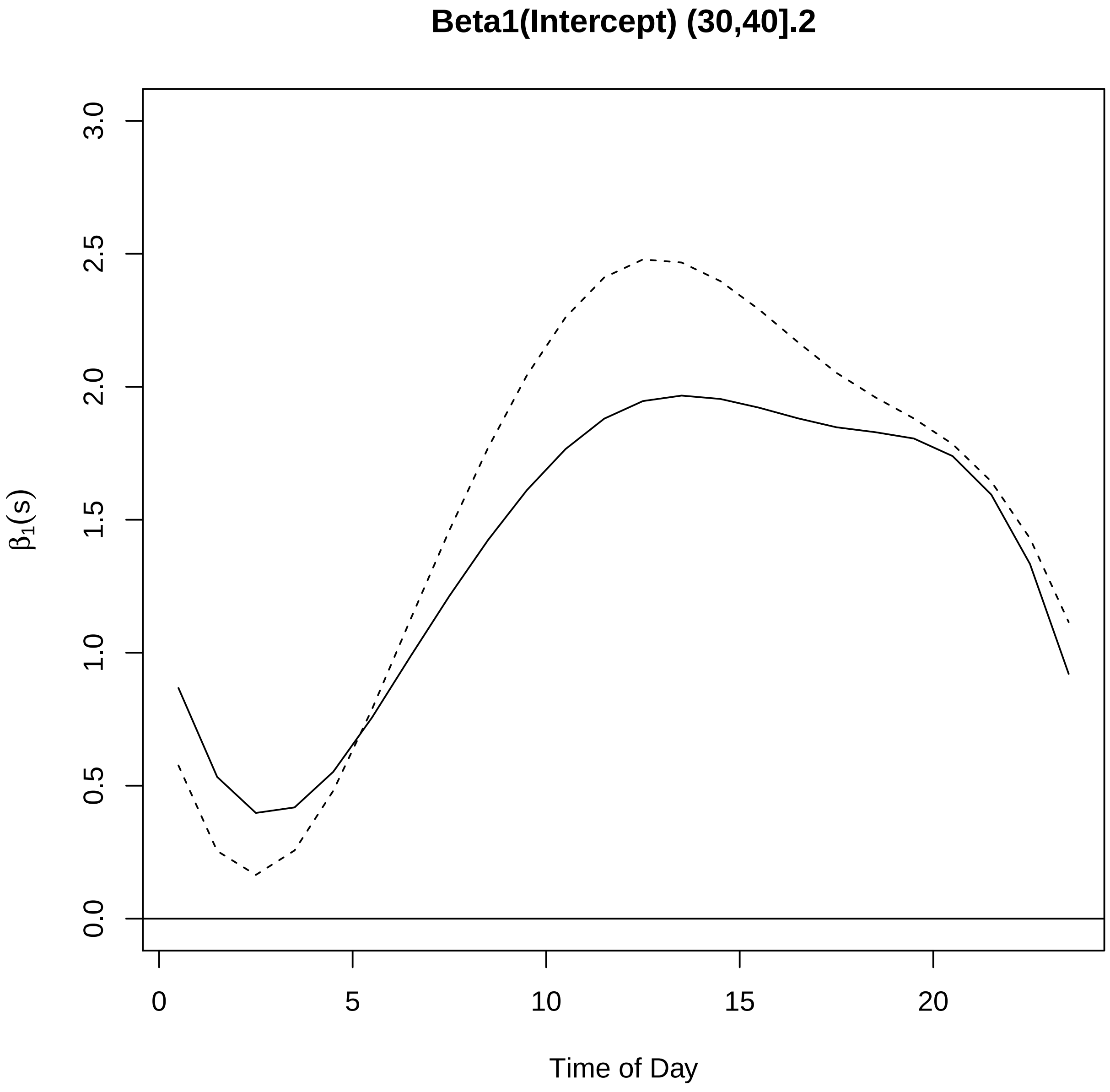

Figure S10: Predicted functional trajectories $(\hat{\beta}_1(s) + \hat{\beta}_{1i}(s))$ of diurnal MIMS for males from the MuFuMES method in the NHANES application. The other continuous covariates were held at their average values. The solid line corresponds to the fixed effect prediction $\hat{\beta}_1(s)$ and the dashed line corresponds to the mixed effects prediction $\hat{\beta}_1(s) + \hat{\beta}_{1i}(s)$. The age-by-race groups are indicated in the header.

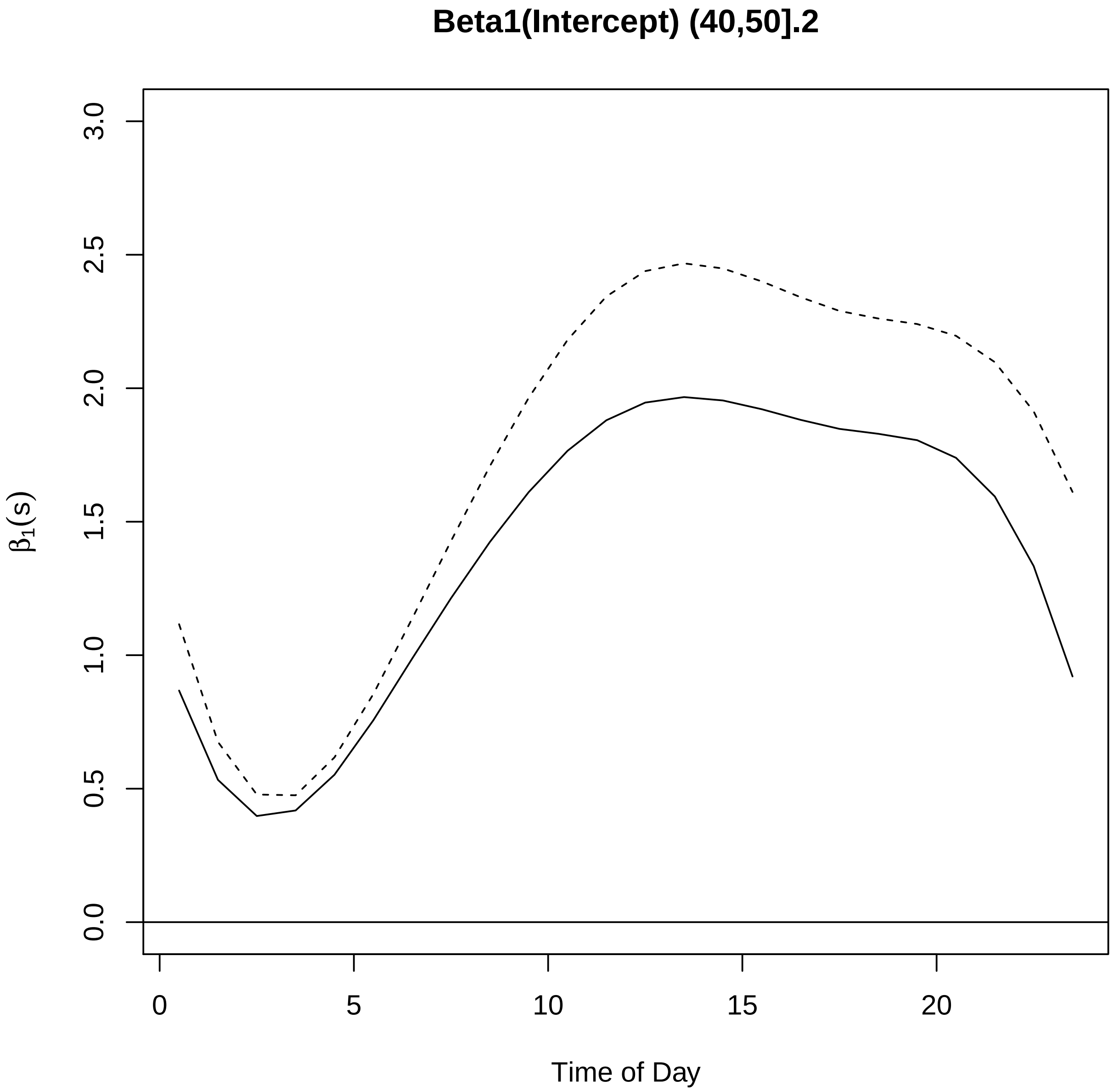

Figure S11: Predicted functional trajectories $(\hat{\beta}_1(s) + \hat{\beta}_{1i}(s))$ of diurnal MIMS for males from the MuFuMES method in the NHANES application. The other continuous covariates were held at their average values. The solid line corresponds to the fixed effect prediction $\hat{\beta}_1(s)$ and the dashed line corresponds to the mixed effects prediction $\hat{\beta}_1(s) + \hat{\beta}_{1i}(s)$. The age-by-race groups are indicated in the header.

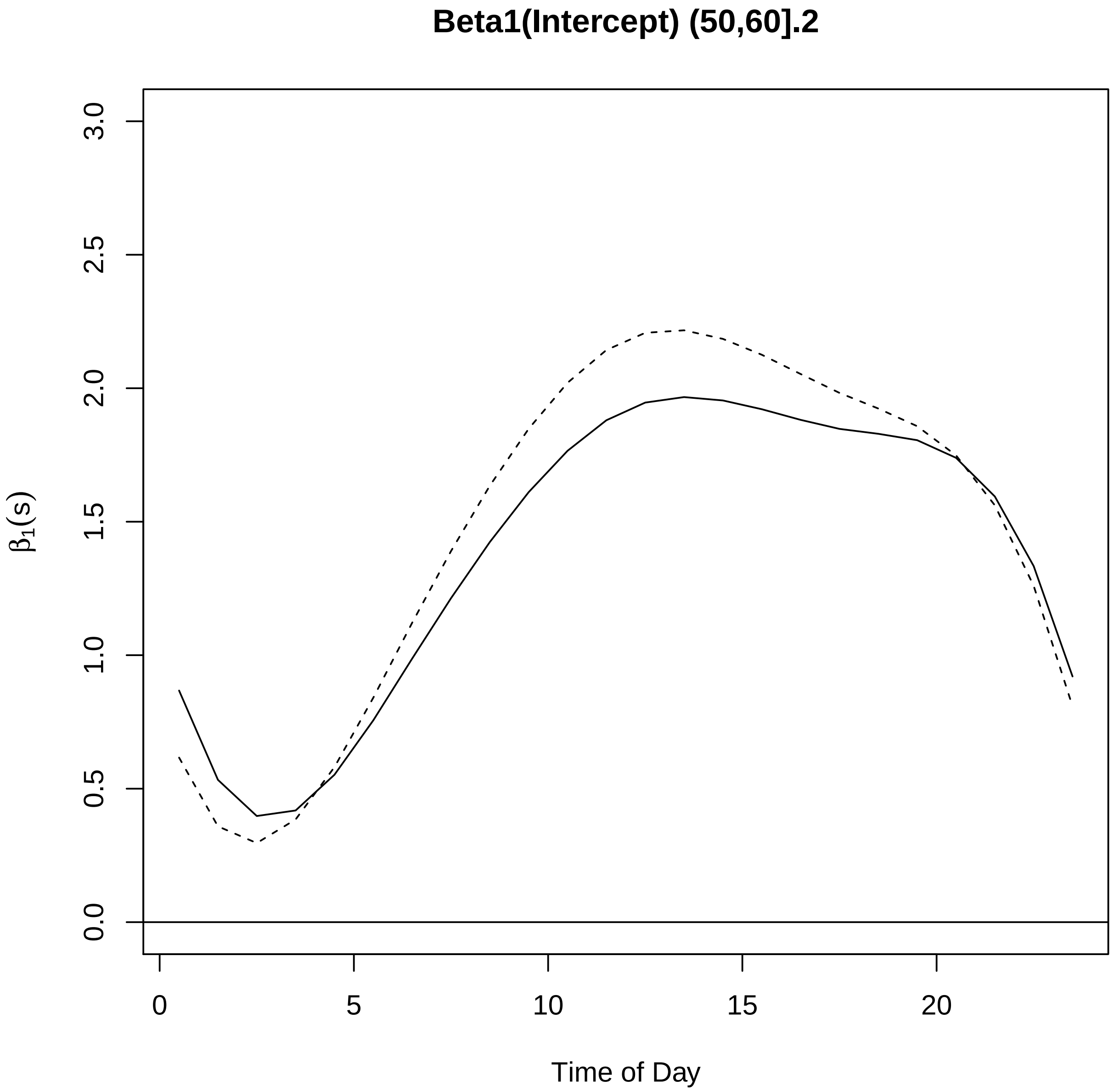

Figure S12: Predicted functional trajectories $(\hat{\beta}_1(s) + \hat{\beta}_{1i}(s))$ of diurnal MIMS for males from the MuFuMES method in the NHANES application. The other continuous covariates were held at their average values. The solid line corresponds to the fixed effect prediction $\hat{\beta}_1(s)$ and the dashed line corresponds to the mixed effects prediction $\hat{\beta}_1(s) + \hat{\beta}_{1i}(s)$. The age-by-race groups are indicated in the header.

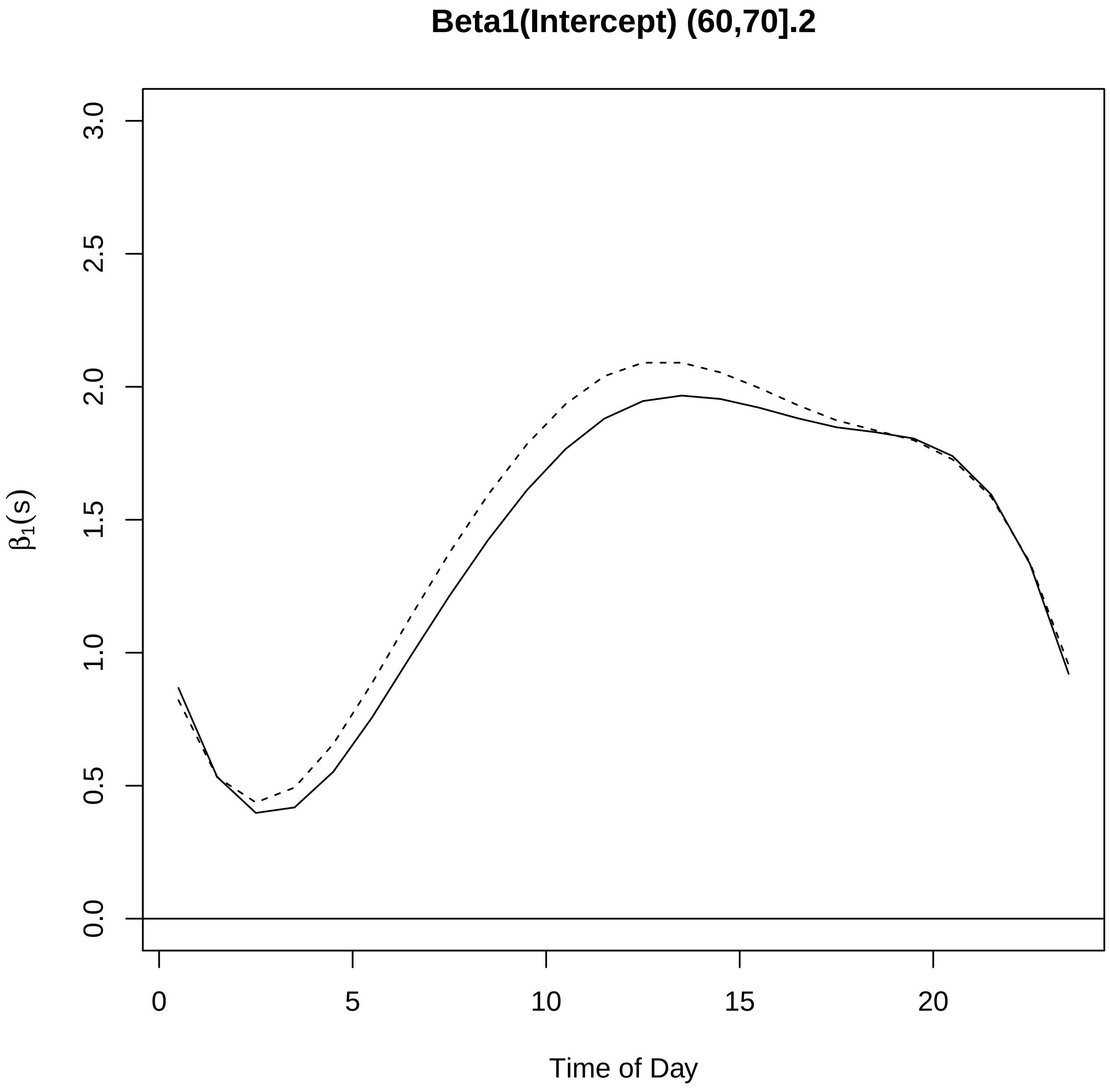

Figure S13: Predicted functional trajectories $(\hat{\beta}_1(s) + \hat{\beta}_{1i}(s))$ of diurnal MIMS for males from the MuFuMES method in the NHANES application. The other continuous covariates were held at their average values. The solid line corresponds to the fixed effect prediction $\hat{\beta}_1(s)$ and the dashed line corresponds to the mixed effects prediction $\hat{\beta}_1(s) + \hat{\beta}_{1i}(s)$. The age-by-race groups are indicated in the header.

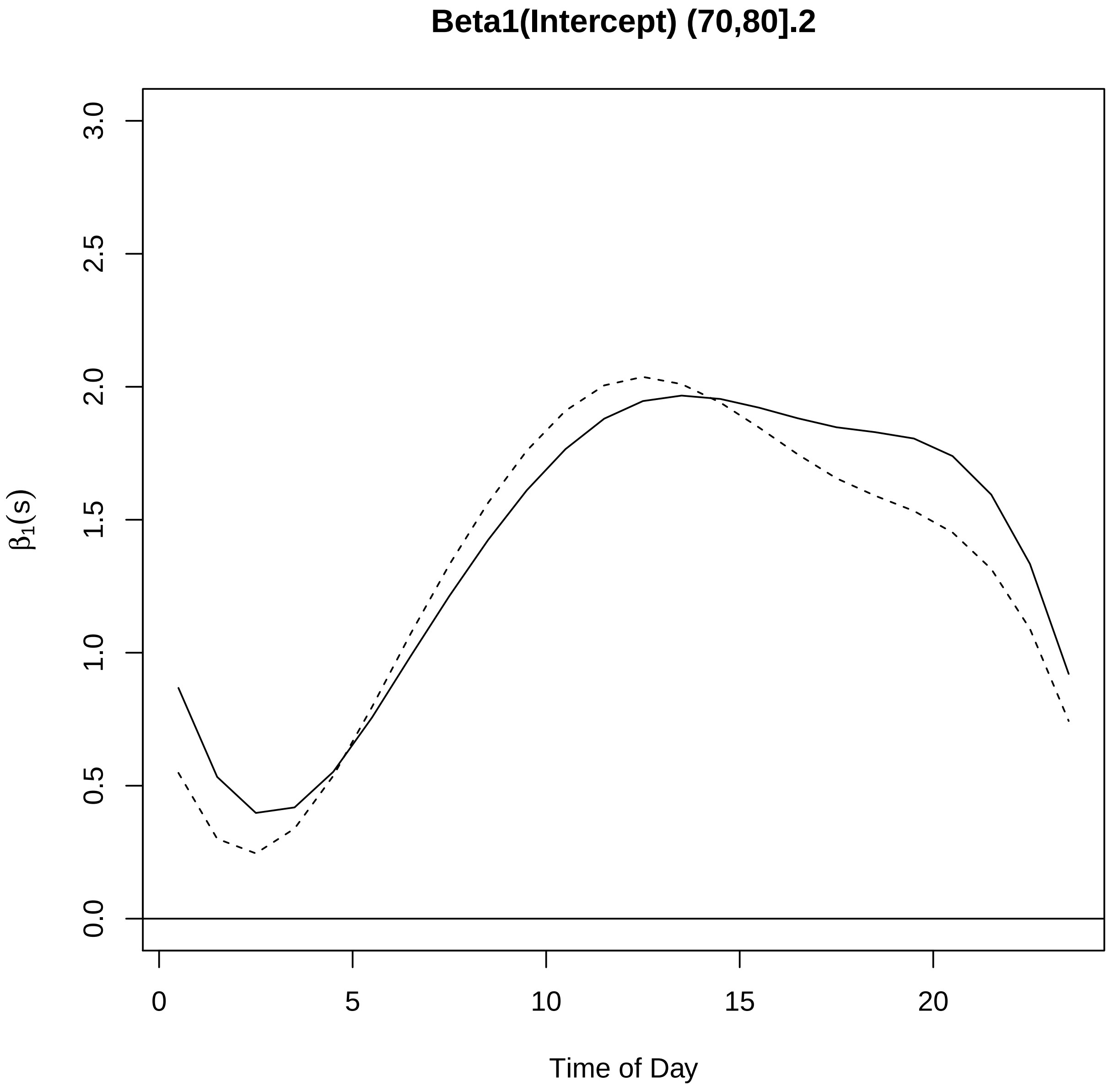

Figure S14: Predicted functional trajectories $(\hat{\beta}_1(s) + \hat{\beta}_{1i}(s))$ of diurnal MIMS for males from the MuFuMES method in the NHANES application. The other continuous covariates were held at their average values. The solid line corresponds to the fixed effect prediction $\hat{\beta}_1(s)$ and the dashed line corresponds to the mixed effects prediction $\hat{\beta}_1(s) + \hat{\beta}_{1i}(s)$. The age-by-race groups are indicated in the header.

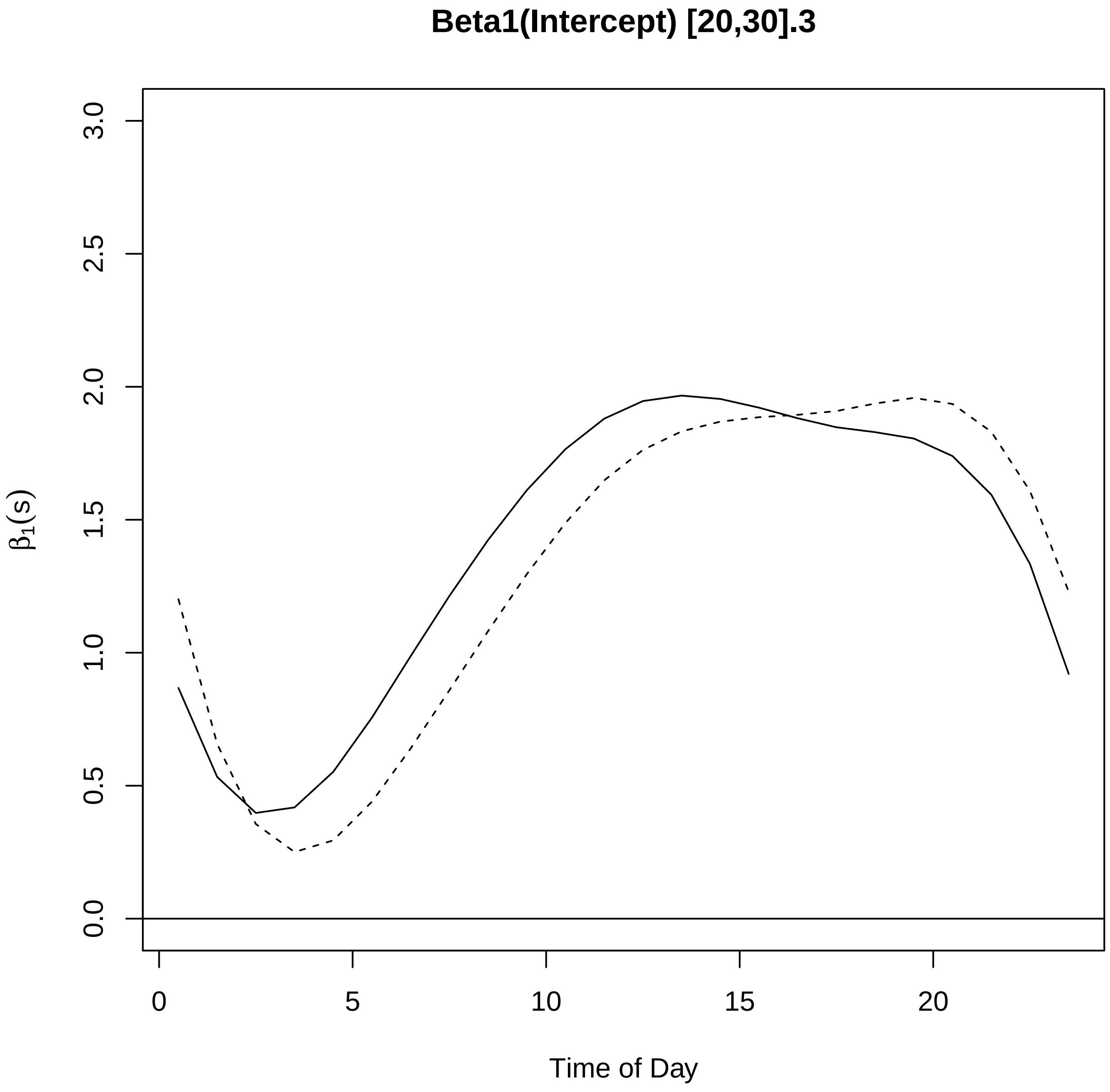

Figure S15: Predicted functional trajectories $(\hat{\beta}_1(s) + \hat{\beta}_{1i}(s))$ of diurnal MIMS for males from the MuFuMES method in the NHANES application. The other continuous covariates were held at their average values. The solid line corresponds to the fixed effect prediction $\hat{\beta}_1(s)$ and the dashed line corresponds to the mixed effects prediction $\hat{\beta}_1(s) + \hat{\beta}_{1i}(s)$. The age-by-race groups are indicated in the header.

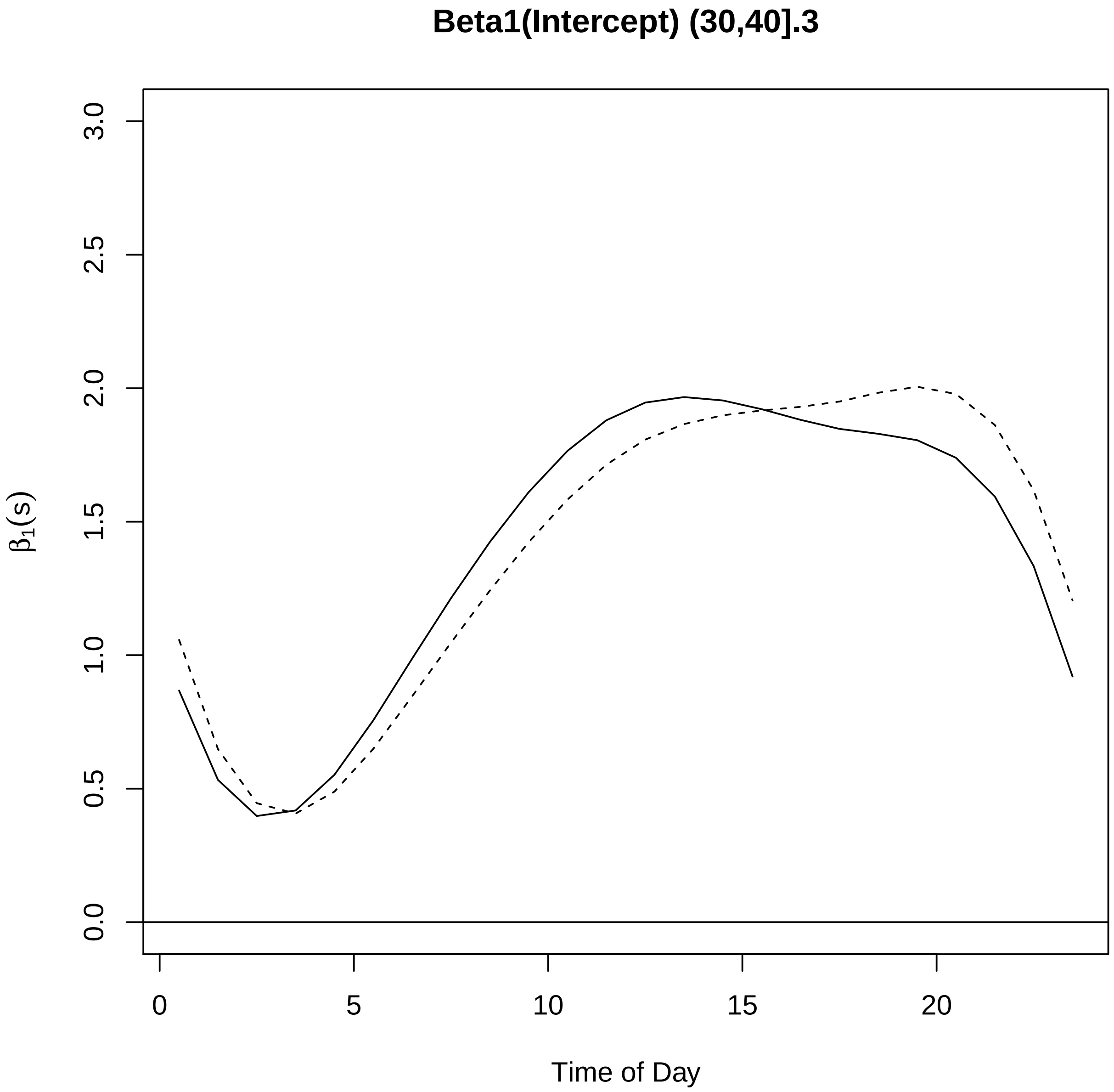

Figure S16: Predicted functional trajectories $(\hat{\beta}_1(s) + \hat{\beta}_{1i}(s))$ of diurnal MIMS for males from the MuFuMES method in the NHANES application. The other continuous covariates were held at their average values. The solid line corresponds to the fixed effect prediction $\hat{\beta}_1(s)$ and the dashed line corresponds to the mixed effects prediction $\hat{\beta}_1(s) + \hat{\beta}_{1i}(s)$. The age-by-race groups are indicated in the header.

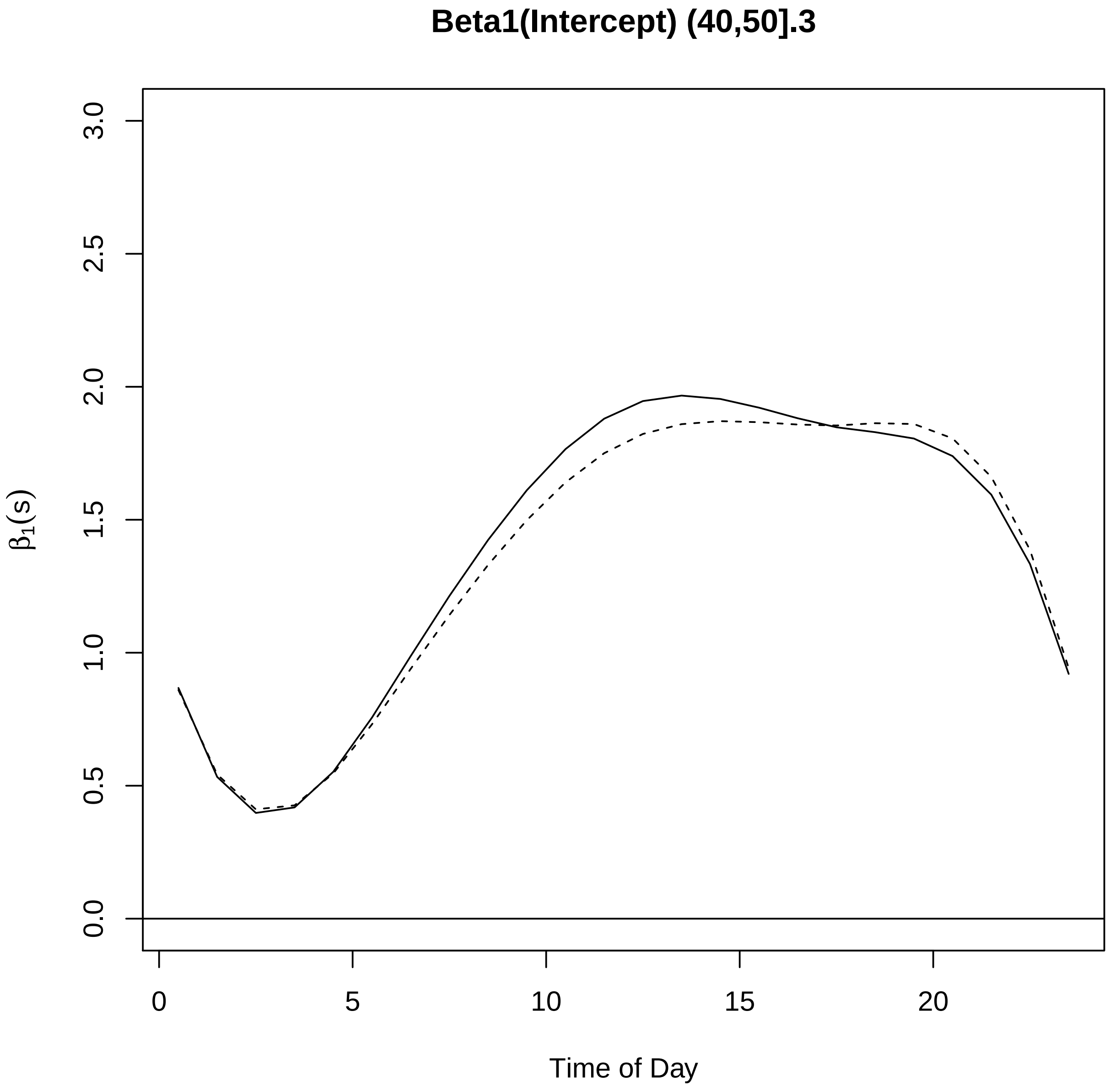

Figure S17: Predicted functional trajectories $(\hat{\beta}_1(s) + \hat{\beta}_{1i}(s))$ of diurnal MIMS for males from the MuFuMES method in the NHANES application. The other continuous covariates were held at their average values. The solid line corresponds to the fixed effect prediction $\hat{\beta}_1(s)$ and the dashed line corresponds to the mixed effects prediction $\hat{\beta}_1(s) + \hat{\beta}_{1i}(s)$. The age-by-race groups are indicated in the header.

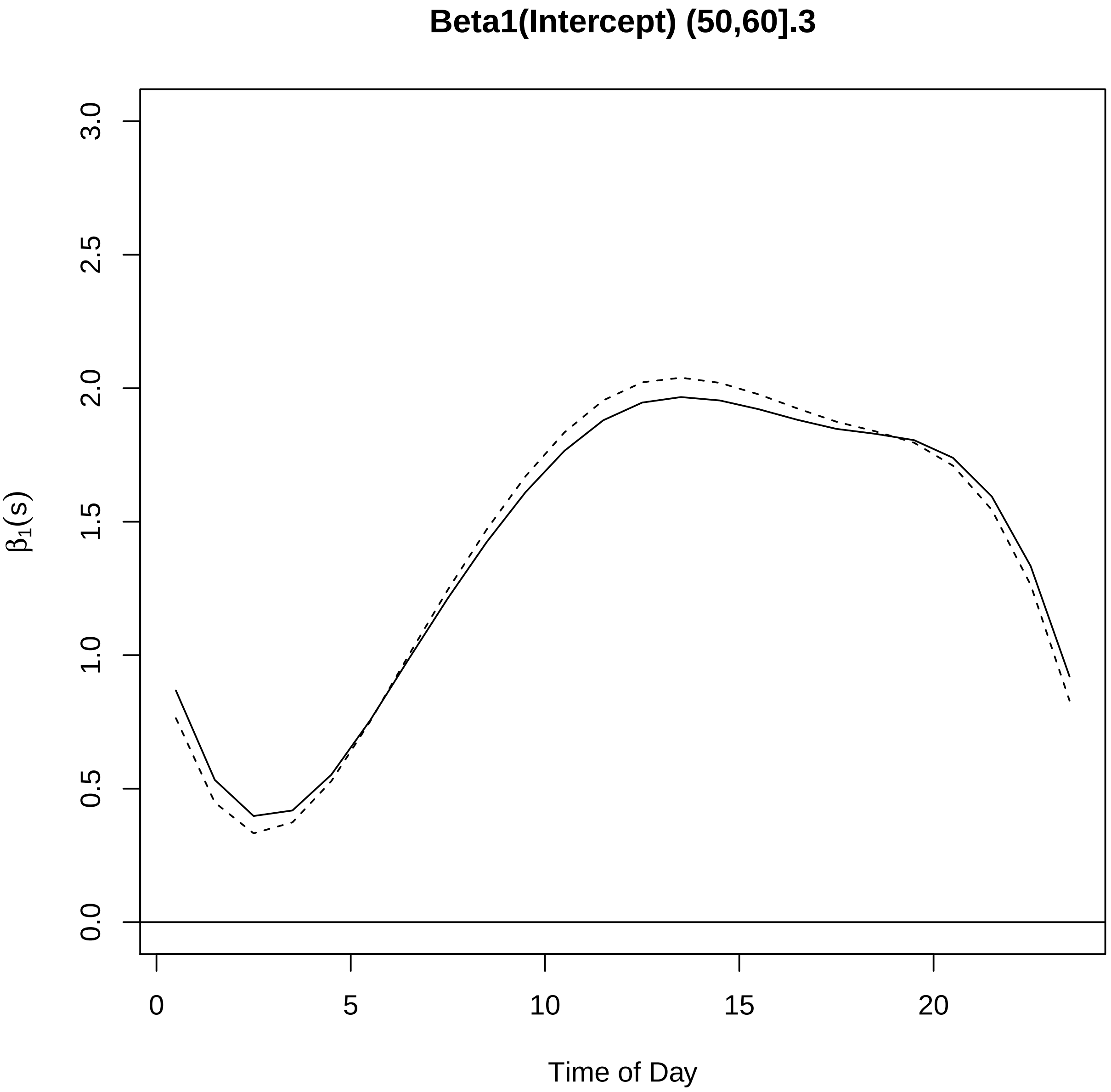

Figure S18: Predicted functional trajectories $(\hat{\beta}_1(s) + \hat{\beta}_{1i}(s))$ of diurnal MIMS for males from the MuFuMES method in the NHANES application. The other continuous covariates were held at their average values. The solid line corresponds to the fixed effect prediction $\hat{\beta}_1(s)$ and the dashed line corresponds to the mixed effects prediction $\hat{\beta}_1(s) + \hat{\beta}_{1i}(s)$. The age-by-race groups are indicated in the header.

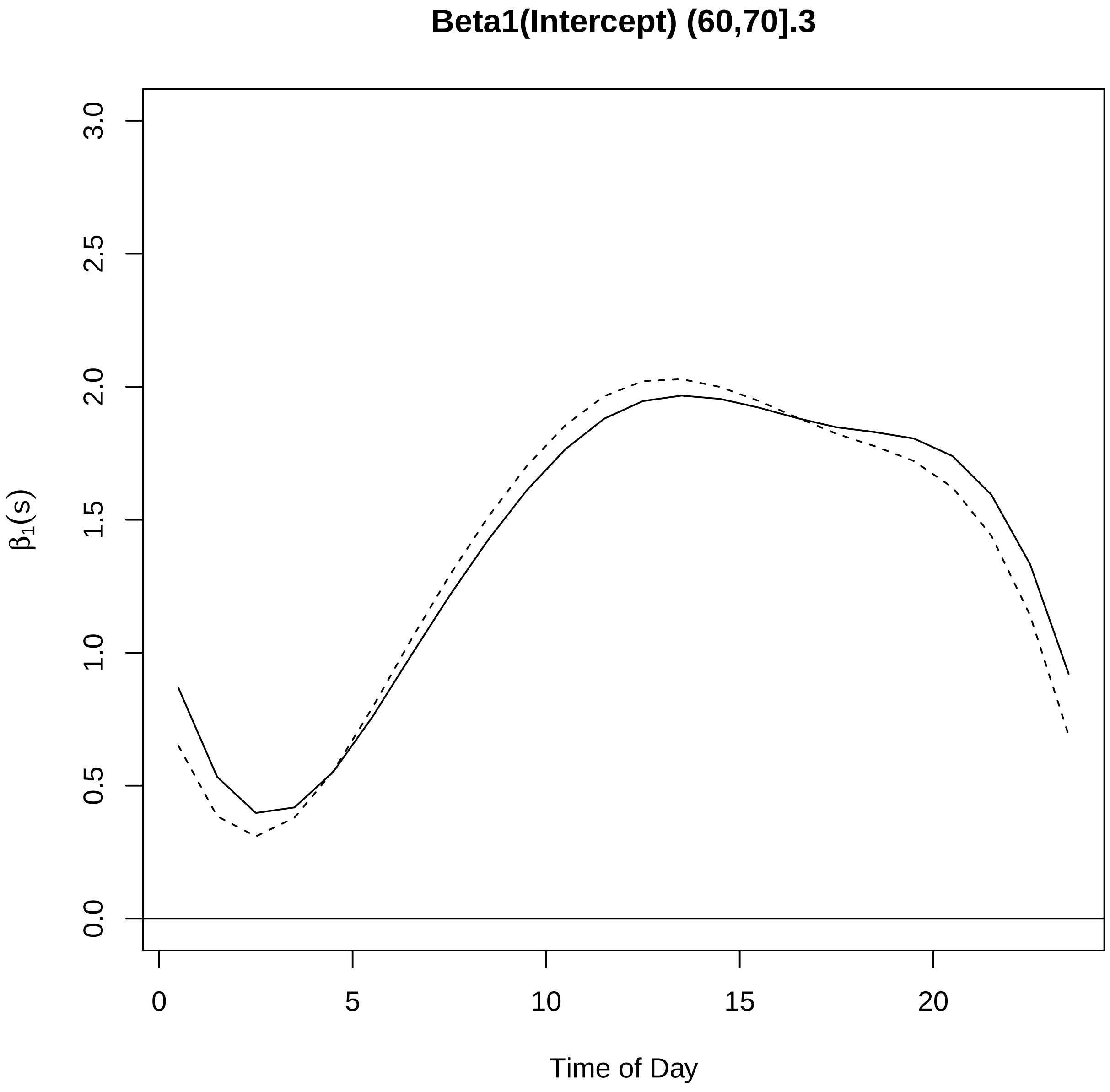

Figure S19: Predicted functional trajectories $(\hat{\beta}_1(s) + \hat{\beta}_{1i}(s))$ of diurnal MIMS for males from the MuFuMES method in the NHANES application. The other continuous covariates were held at their average values. The solid line corresponds to the fixed effect prediction $\hat{\beta}_1(s)$ and the dashed line corresponds to the mixed effects prediction $\hat{\beta}_1(s) + \hat{\beta}_{1i}(s)$. The age-by-race groups are indicated in the header.

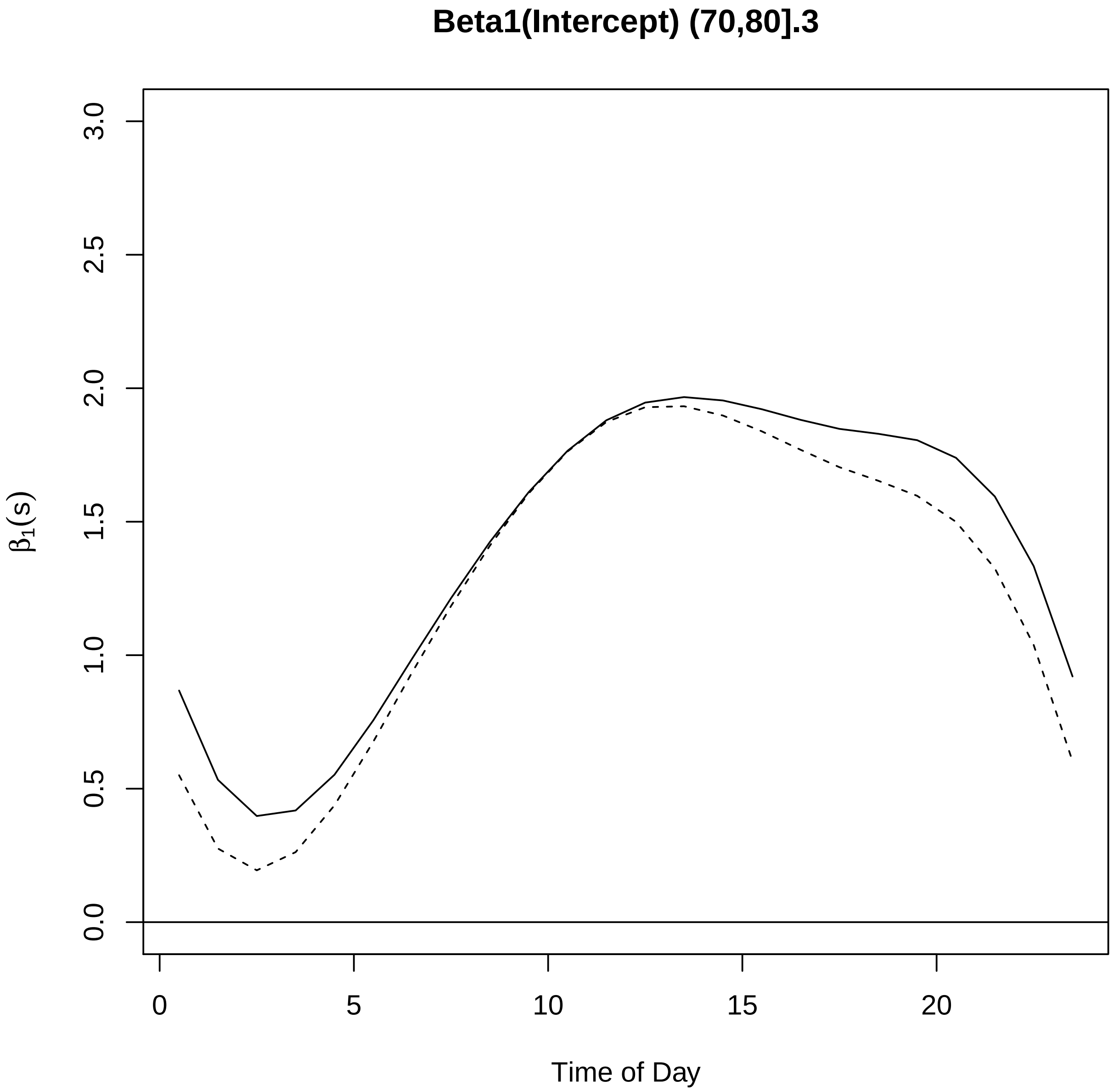

Figure S20: Predicted functional trajectories $(\hat{\beta}_1(s) + \hat{\beta}_{1i}(s))$ of diurnal MIMS for males from the MuFuMES method in the NHANES application. The other continuous covariates were held at their average values. The solid line corresponds to the fixed effect prediction $\hat{\beta}_1(s)$ and the dashed line corresponds to the mixed effects prediction $\hat{\beta}_1(s) + \hat{\beta}_{1i}(s)$. The age-by-race groups are indicated in the header.

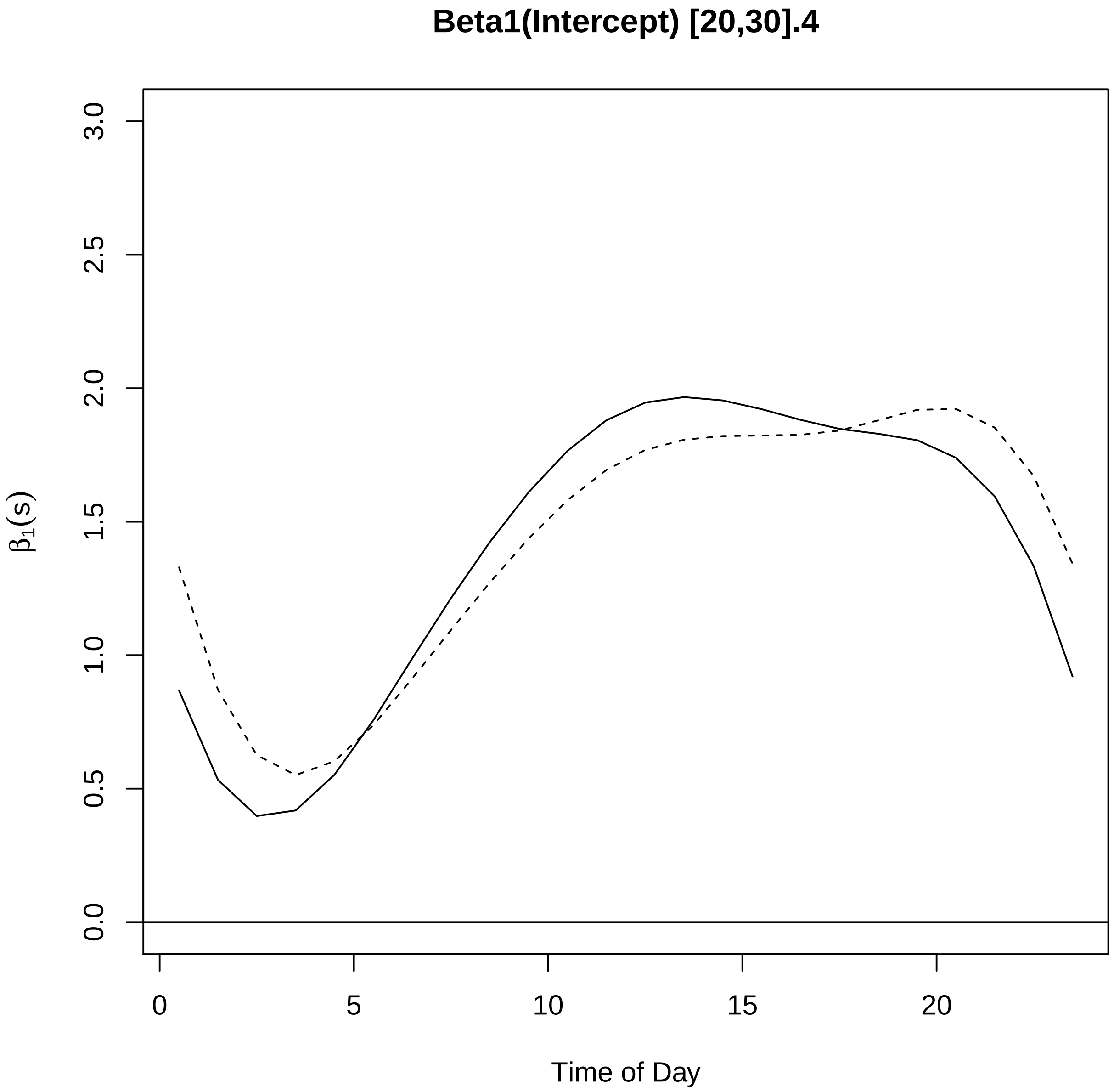

Figure S21: Predicted functional trajectories $(\hat{\beta}_1(s) + \hat{\beta}_{1i}(s))$ of diurnal MIMS for males from the MuFuMES method in the NHANES application. The other continuous covariates were held at their average values. The solid line corresponds to the fixed effect prediction $\hat{\beta}_1(s)$ and the dashed line corresponds to the mixed effects prediction $\hat{\beta}_1(s) + \hat{\beta}_{1i}(s)$. The age-by-race groups are indicated in the header.

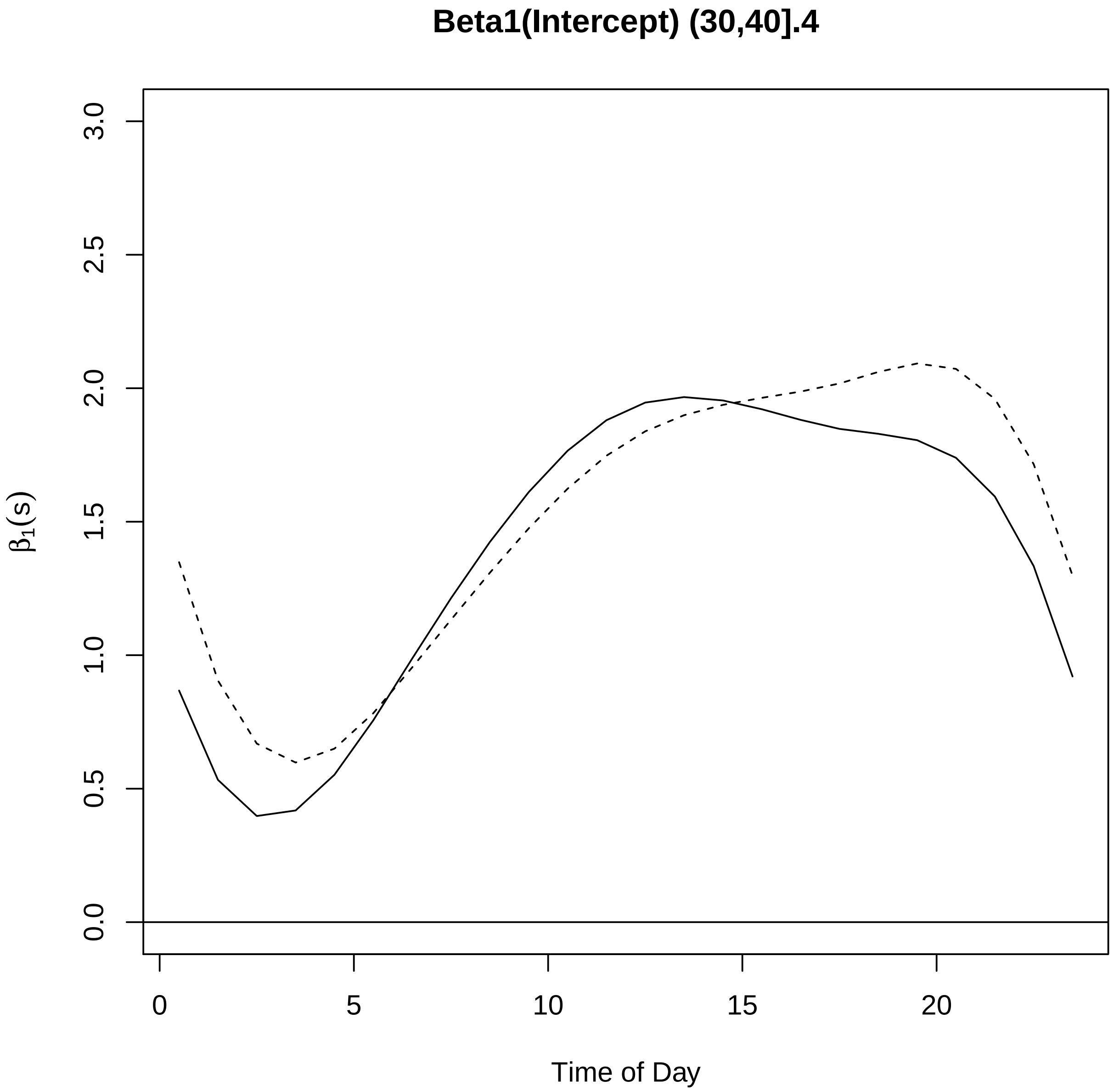

Figure S22: Predicted functional trajectories $(\hat{\beta}_1(s) + \hat{\beta}_{1i}(s))$ of diurnal MIMS for males from the MuFuMES method in the NHANES application. The other continuous covariates were held at their average values. The solid line corresponds to the fixed effect prediction $\hat{\beta}_1(s)$ and the dashed line corresponds to the mixed effects prediction $\hat{\beta}_1(s) + \hat{\beta}_{1i}(s)$. The age-by-race groups are indicated in the header.

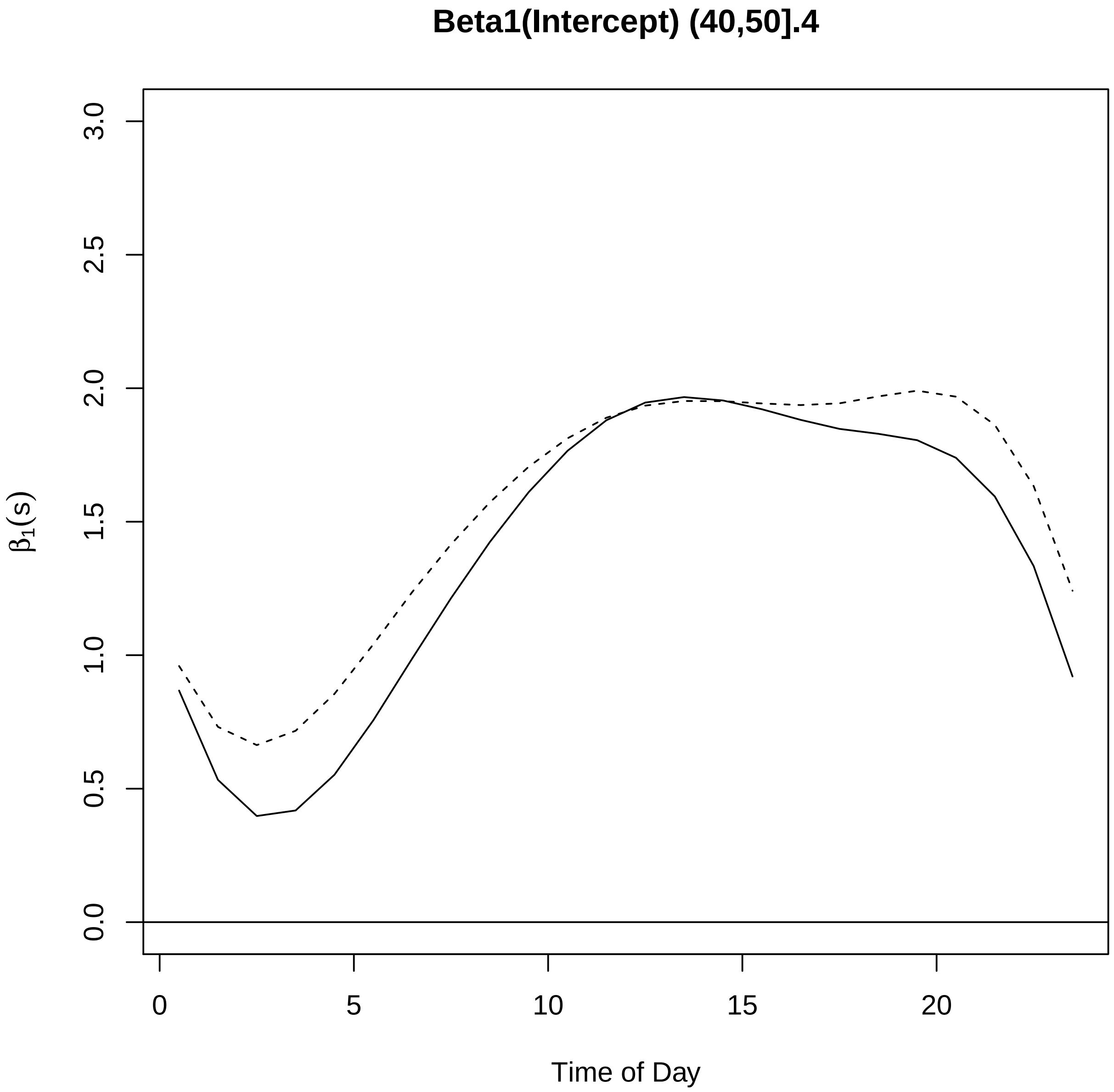

Figure S23: Predicted functional trajectories $(\hat{\beta}_1(s) + \hat{\beta}_{1i}(s))$ of diurnal MIMS for males from the MuFuMES method in the NHANES application. The other continuous covariates were held at their average values. The solid line corresponds to the fixed effect prediction $\hat{\beta}_1(s)$ and the dashed line corresponds to the mixed effects prediction $\hat{\beta}_1(s) + \hat{\beta}_{1i}(s)$. The age-by-race groups are indicated in the header.

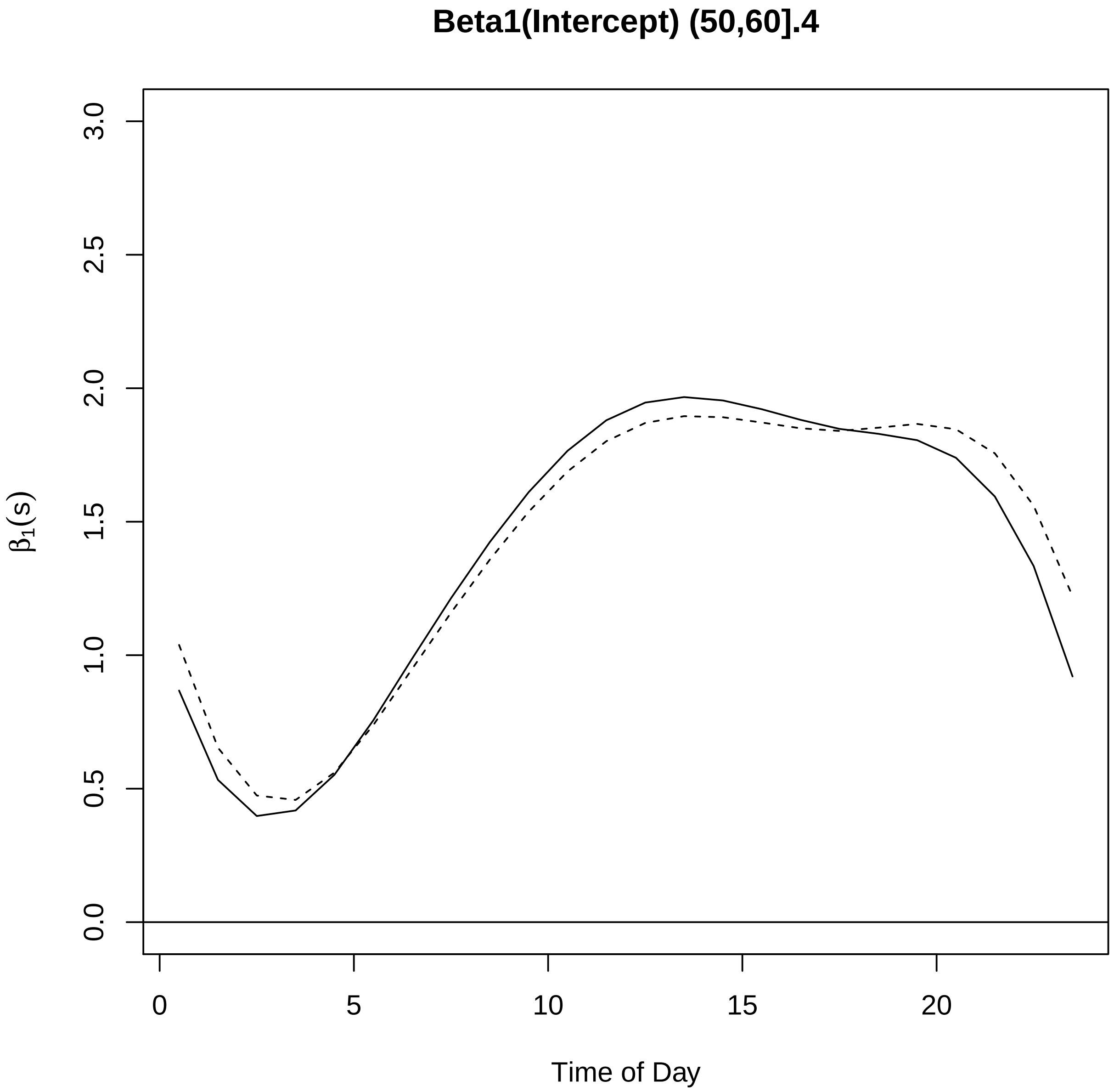

Figure S24: Predicted functional trajectories $(\hat{\beta}_1(s) + \hat{\beta}_{1i}(s))$ of diurnal MIMS for males from the MuFuMES method in the NHANES application. The other continuous covariates were held at their average values. The solid line corresponds to the fixed effect prediction $\hat{\beta}_1(s)$ and the dashed line corresponds to the mixed effects prediction $\hat{\beta}_1(s) + \hat{\beta}_{1i}(s)$. The age-by-race groups are indicated in the header.

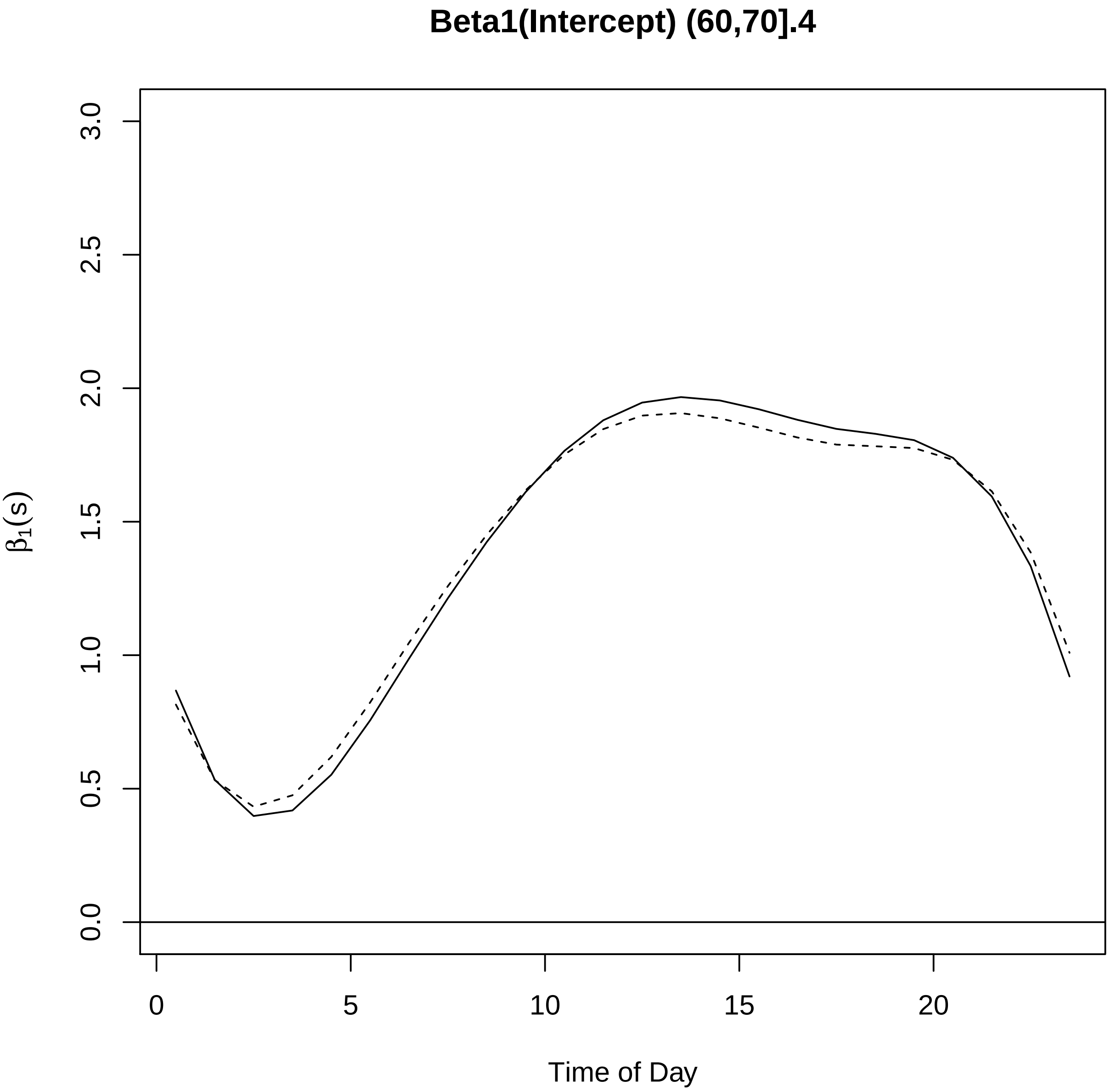

Figure S25: Predicted functional trajectories $(\hat{\beta}_1(s) + \hat{\beta}_{1i}(s))$ of diurnal MIMS for males from the MuFuMES method in the NHANES application. The other continuous covariates were held at their average values. The solid line corresponds to the fixed effect prediction $\hat{\beta}_1(s)$ and the dashed line corresponds to the mixed effects prediction $\hat{\beta}_1(s) + \hat{\beta}_{1i}(s)$. The age-by-race groups are indicated in the header.

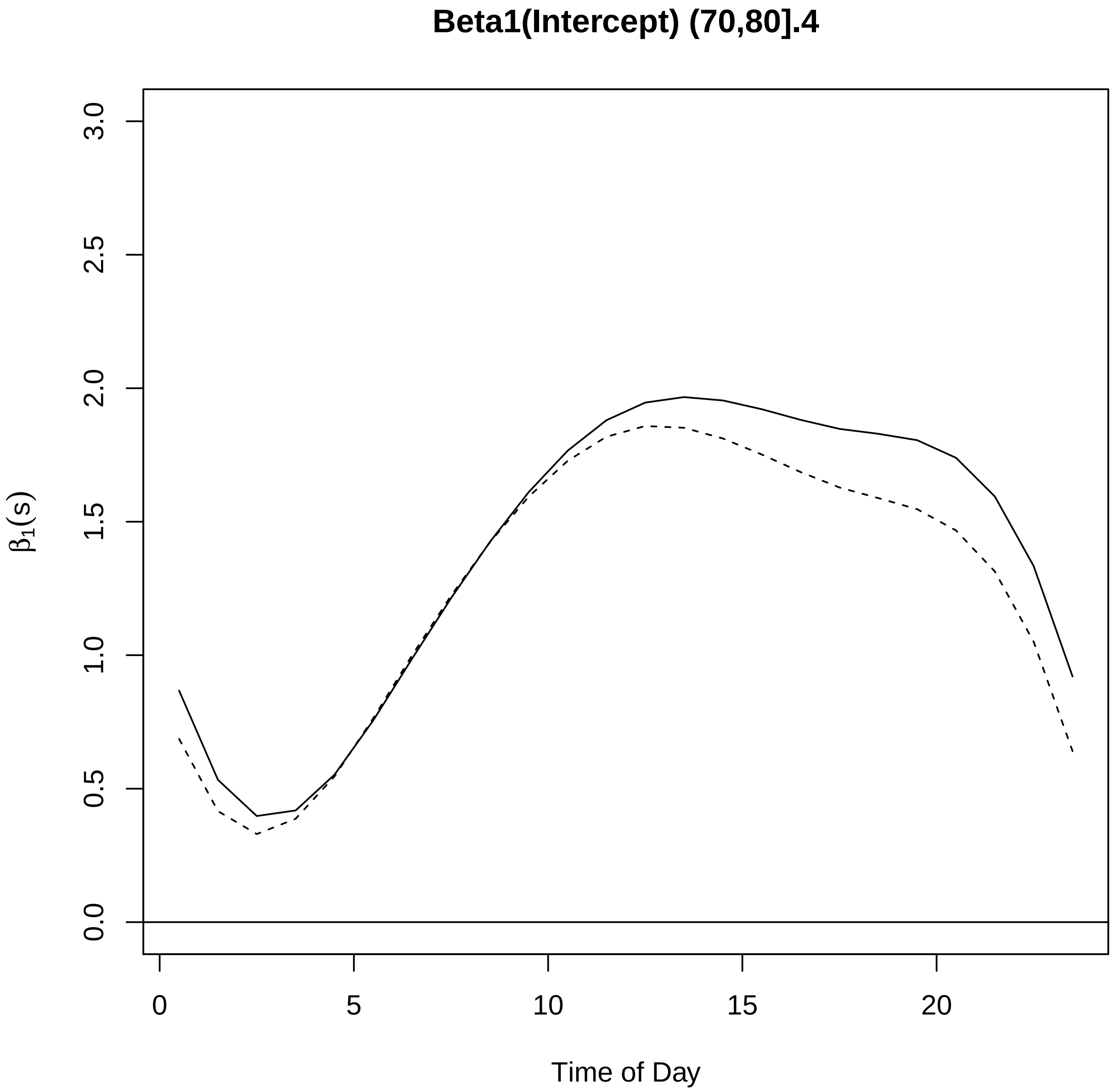

Figure S26: Predicted functional trajectories $(\hat{\beta}_1(s) + \hat{\beta}_{1i}(s))$ of diurnal MIMS for males from the MuFuMES method in the NHANES application. The other continuous covariates were held at their average values. The solid line corresponds to the fixed effect prediction $\hat{\beta}_1(s)$ and the dashed line corresponds to the mixed effects prediction $\hat{\beta}_1(s) + \hat{\beta}_{1i}(s)$. The age-by-race groups are indicated in the header.

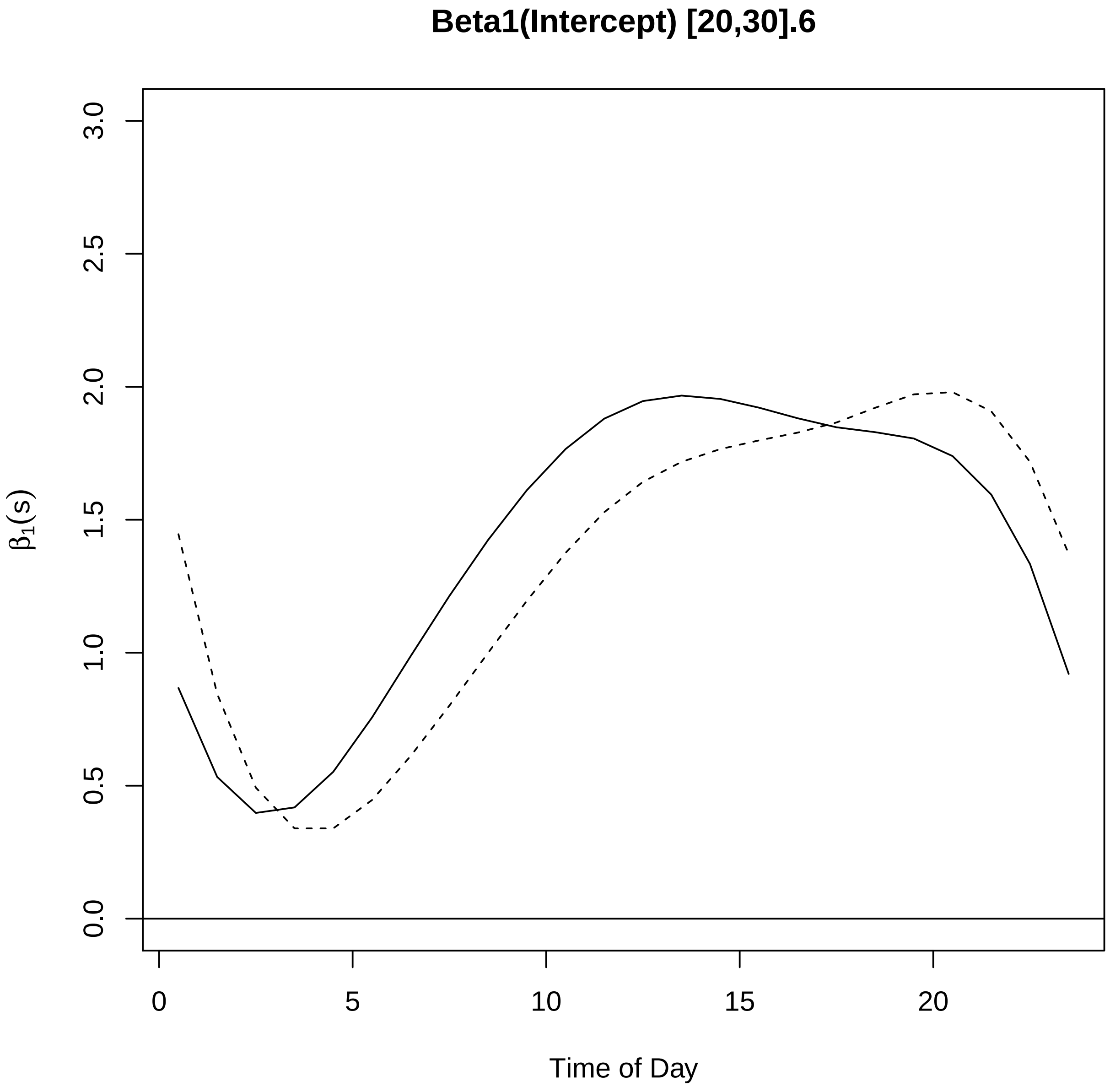

Figure S27: Predicted functional trajectories $(\hat{\beta}_1(s) + \hat{\beta}_{1i}(s))$ of diurnal MIMS for males from the MuFuMES method in the NHANES application. The other continuous covariates were held at their average values. The solid line corresponds to the fixed effect prediction $\hat{\beta}_1(s)$ and the dashed line corresponds to the mixed effects prediction $\hat{\beta}_1(s) + \hat{\beta}_{1i}(s)$. The age-by-race groups are indicated in the header.

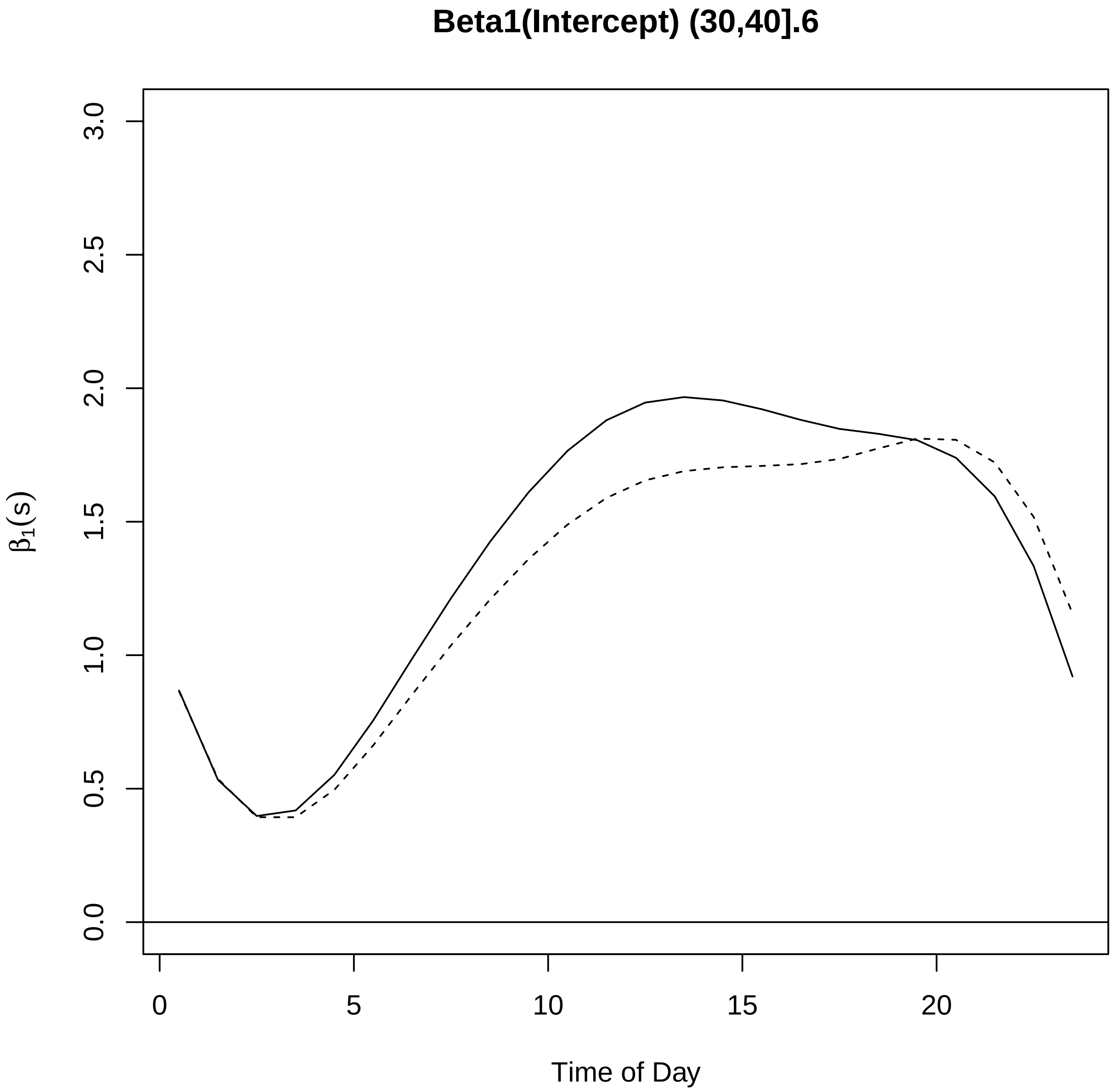

Figure S28: Predicted functional trajectories $(\hat{\beta}_1(s) + \hat{\beta}_{1i}(s))$ of diurnal MIMS for males from the MuFuMES method in the NHANES application. The other continuous covariates were held at their average values. The solid line corresponds to the fixed effect prediction $\hat{\beta}_1(s)$ and the dashed line corresponds to the mixed effects prediction $\hat{\beta}_1(s) + \hat{\beta}_{1i}(s)$. The age-by-race groups are indicated in the header.

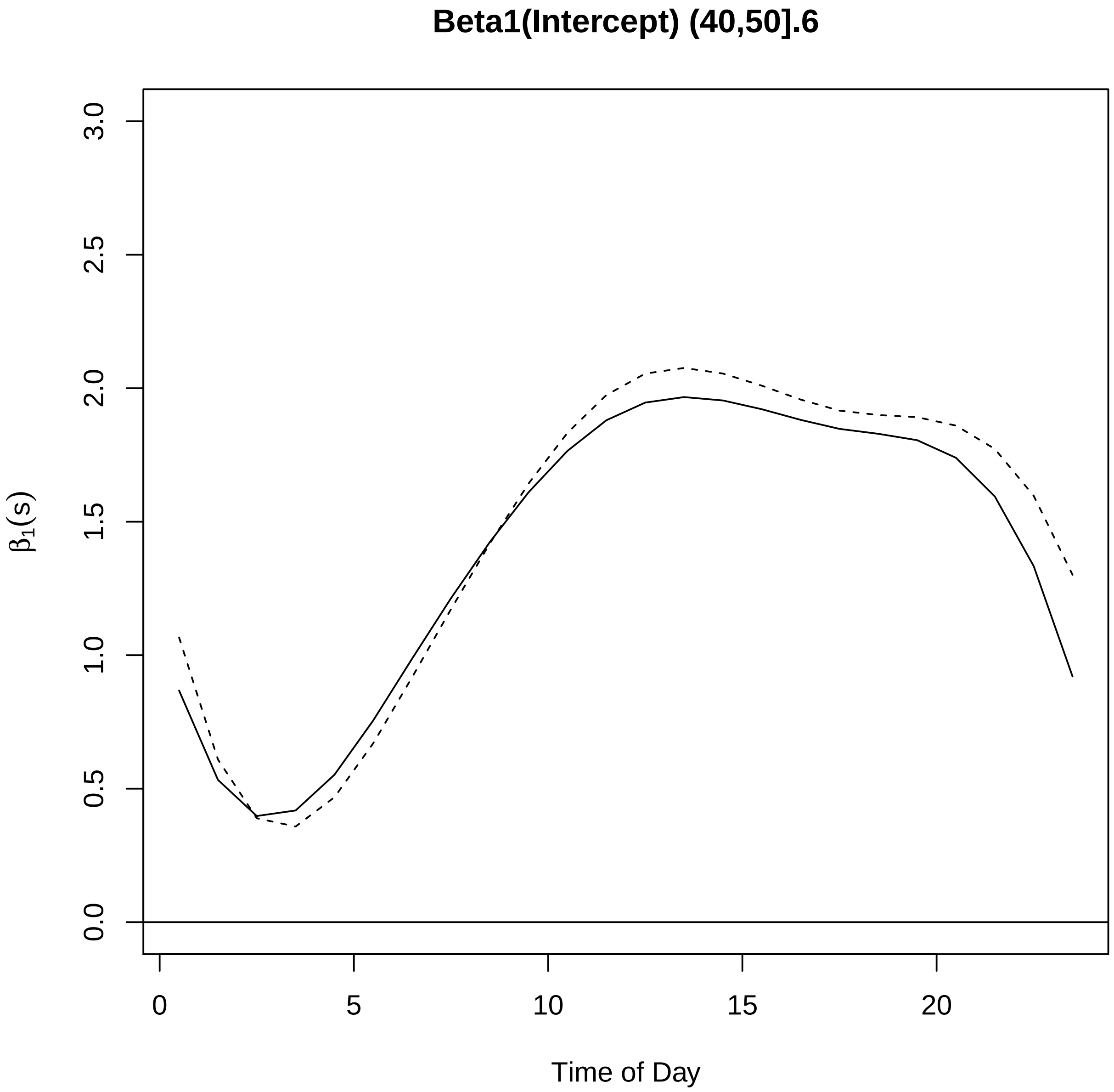

Figure S29: Predicted functional trajectories $(\hat{\beta}_1(s) + \hat{\beta}_{1i}(s))$ of diurnal MIMS for males from the MuFuMES method in the NHANES application. The other continuous covariates were held at their average values. The solid line corresponds to the fixed effect prediction $\hat{\beta}_1(s)$ and the dashed line corresponds to the mixed effects prediction $\hat{\beta}_1(s) + \hat{\beta}_{1i}(s)$. The age-by-race groups are indicated in the header.

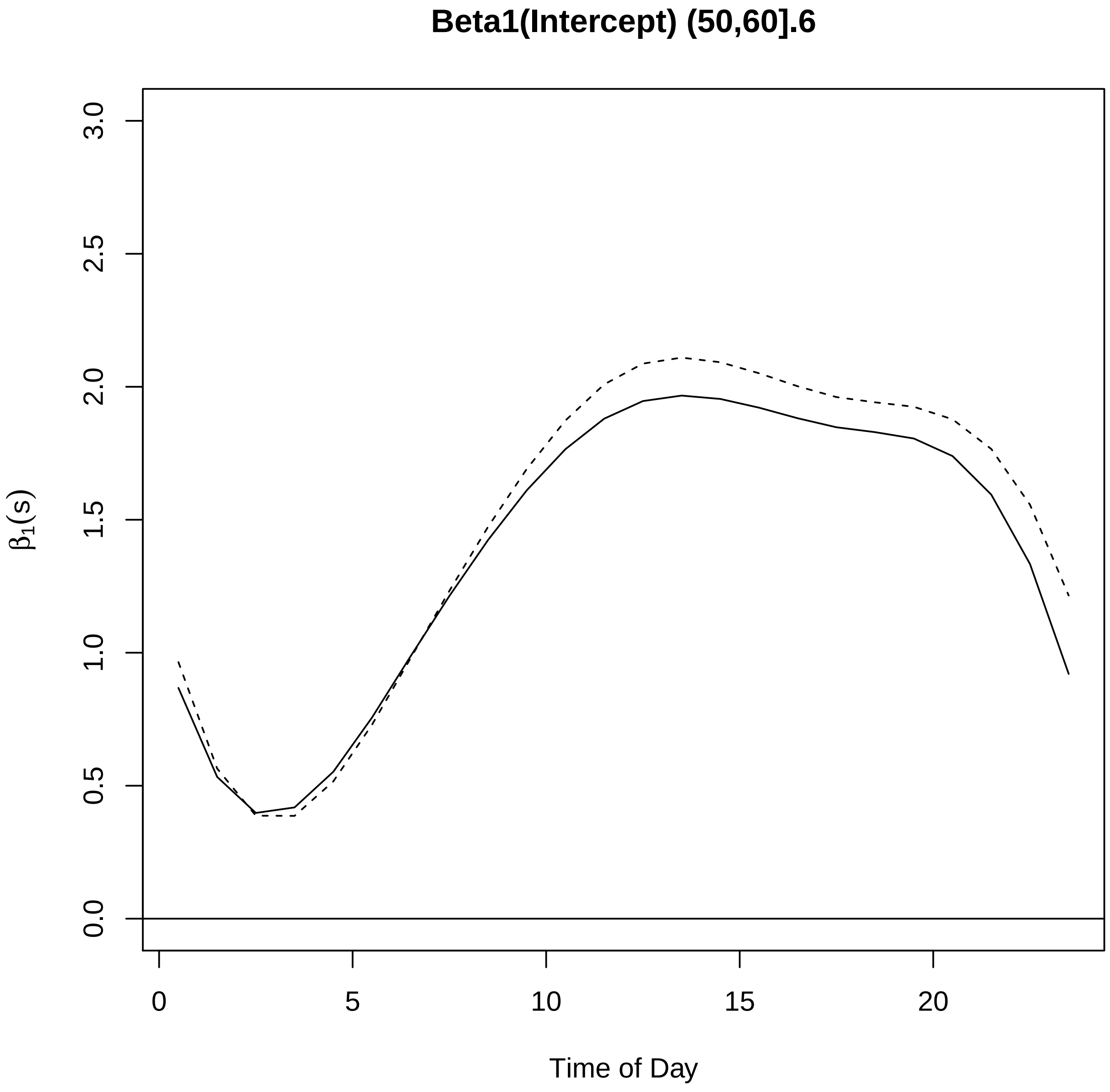

Figure S30: Predicted functional trajectories $(\hat{\beta}_1(s) + \hat{\beta}_{1i}(s))$ of diurnal MIMS for males from the MuFuMES method in the NHANES application. The other continuous covariates were held at their average values. The solid line corresponds to the fixed effect prediction $\hat{\beta}_1(s)$ and the dashed line corresponds to the mixed effects prediction $\hat{\beta}_1(s) + \hat{\beta}_{1i}(s)$. The age-by-race groups are indicated in the header.

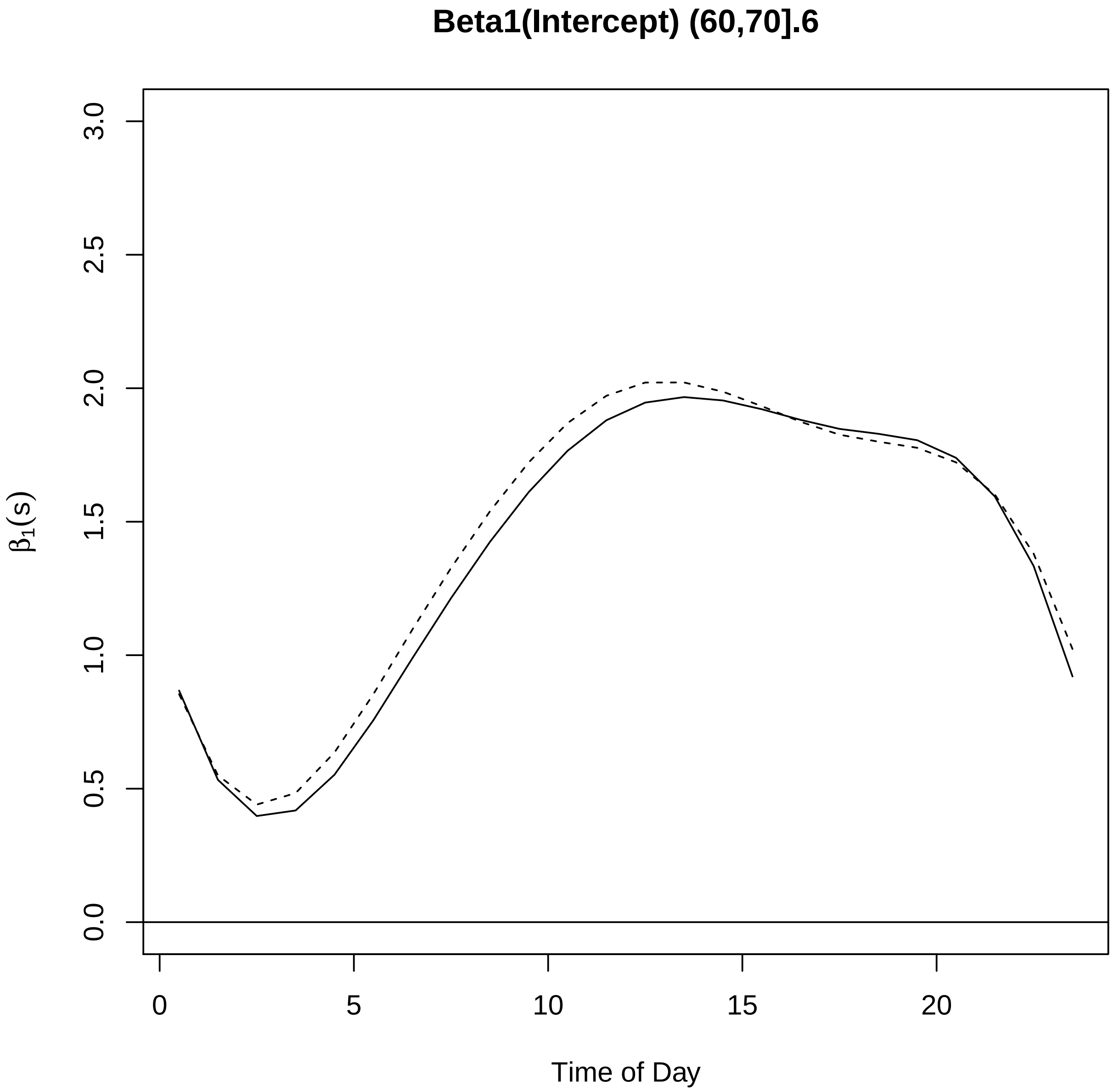

Figure S31: Predicted functional trajectories $(\hat{\beta}_1(s) + \hat{\beta}_{1i}(s))$ of diurnal MIMS for males from the MuFuMES method in the NHANES application. The other continuous covariates were held at their average values. The solid line corresponds to the fixed effect prediction $\hat{\beta}_1(s)$ and the dashed line corresponds to the mixed effects prediction $\hat{\beta}_1(s) + \hat{\beta}_{1i}(s)$. The age-by-race groups are indicated in the header.

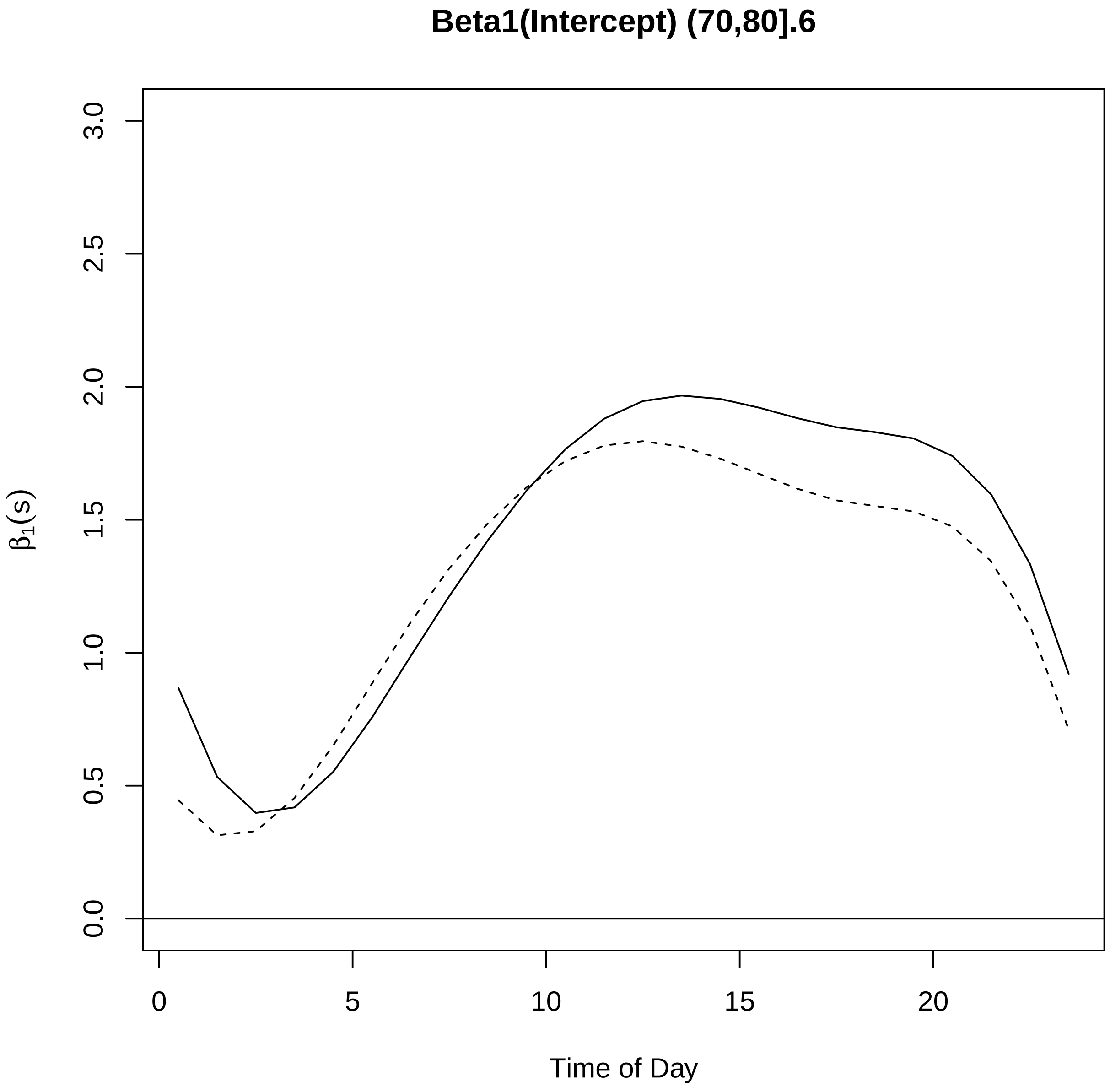

Figure S32: Predicted functional trajectories $(\hat{\beta}_1(s) + \hat{\beta}_{1i}(s))$ of diurnal MIMS for males from the MuFuMES method in the NHANES application. The other continuous covariates were held at their average values. The solid line corresponds to the fixed effect prediction $\hat{\beta}_1(s)$ and the dashed line corresponds to the mixed effects prediction $\hat{\beta}_1(s) + \hat{\beta}_{1i}(s)$. The age-by-race groups are indicated in the header.

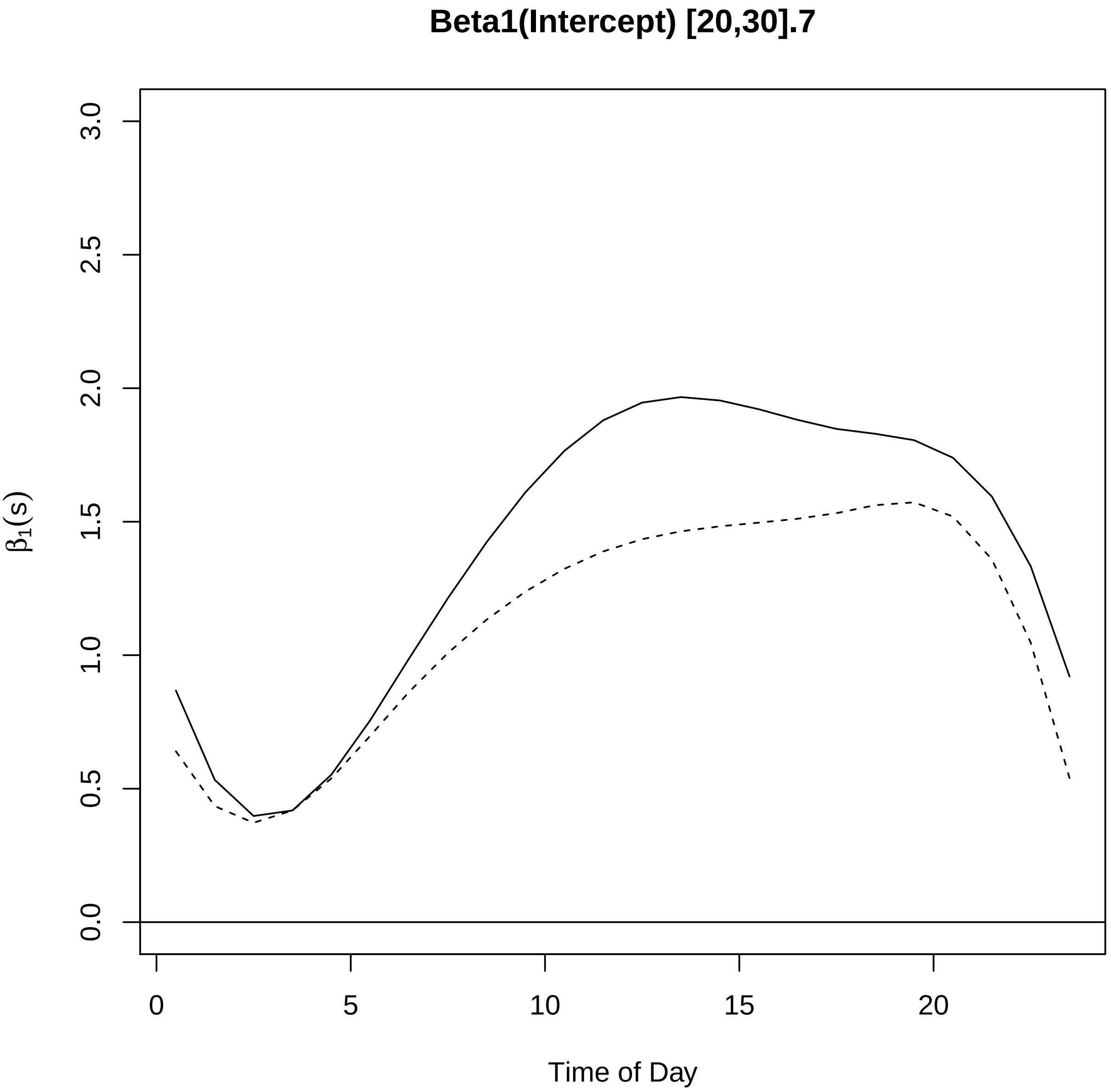

Figure S33: Predicted functional trajectories $(\hat{\beta}_1(s) + \hat{\beta}_{1i}(s))$ of diurnal MIMS for males from the MuFuMES method in the NHANES application. The other continuous covariates were held at their average values. The solid line corresponds to the fixed effect prediction $\hat{\beta}_1(s)$ and the dashed line corresponds to the mixed effects prediction $\hat{\beta}_1(s) + \hat{\beta}_{1i}(s)$. The age-by-race groups are indicated in the header.

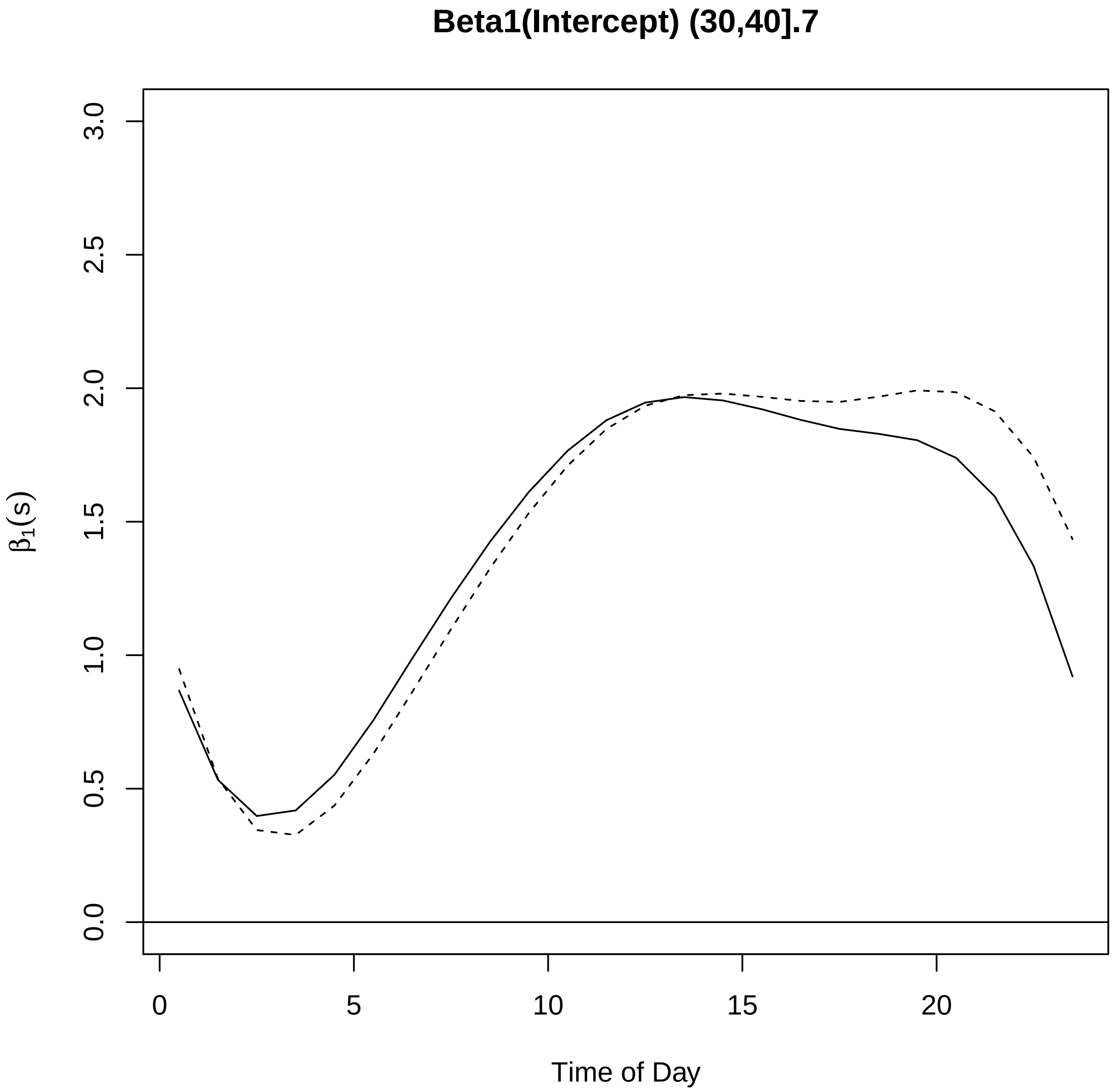

Figure S34: Predicted functional trajectories ($\hat{\beta}_1(s) + \hat{\beta}_{1i}(s)$) of diurnal MIMS for males from the MuFuMES method in the NHANES application. The other continuous covariates were held at their average values. The solid line corresponds to the fixed effect prediction $\hat{\beta}_1(s)$ and the dashed line corresponds to the mixed effects prediction $\hat{\beta}_1(s) + \hat{\beta}_{1i}(s)$. The age-by-race groups are indicated in the header.

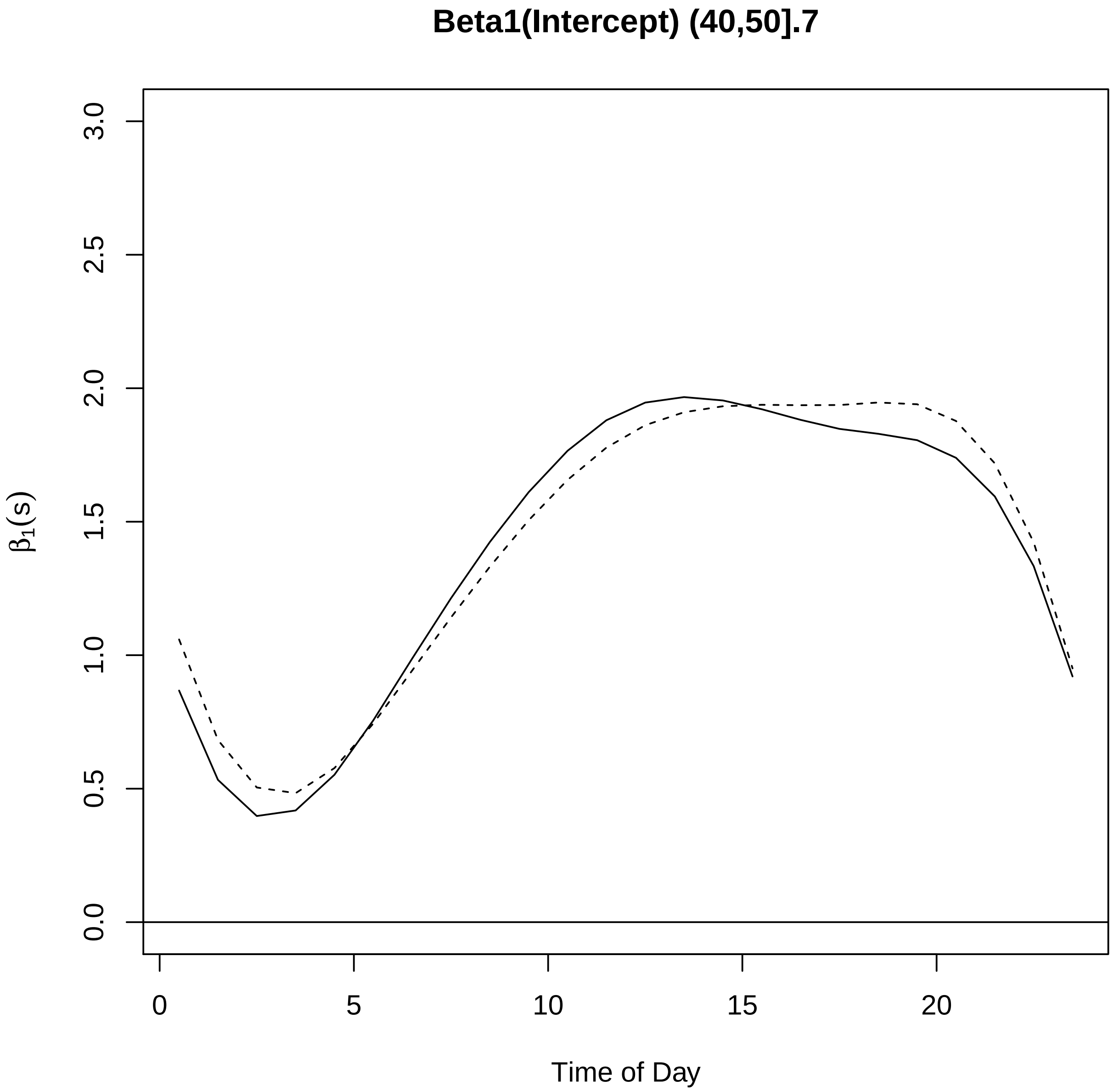

Figure S35: Predicted functional trajectories $(\hat{\beta}_1(s) + \hat{\beta}_{1i}(s))$ of diurnal MIMS for males from the MuFuMES method in the NHANES application. The other continuous covariates were held at their average values. The solid line corresponds to the fixed effect prediction $\hat{\beta}_1(s)$ and the dashed line corresponds to the mixed effects prediction $\hat{\beta}_1(s) + \hat{\beta}_{1i}(s)$. The age-by-race groups are indicated in the header.

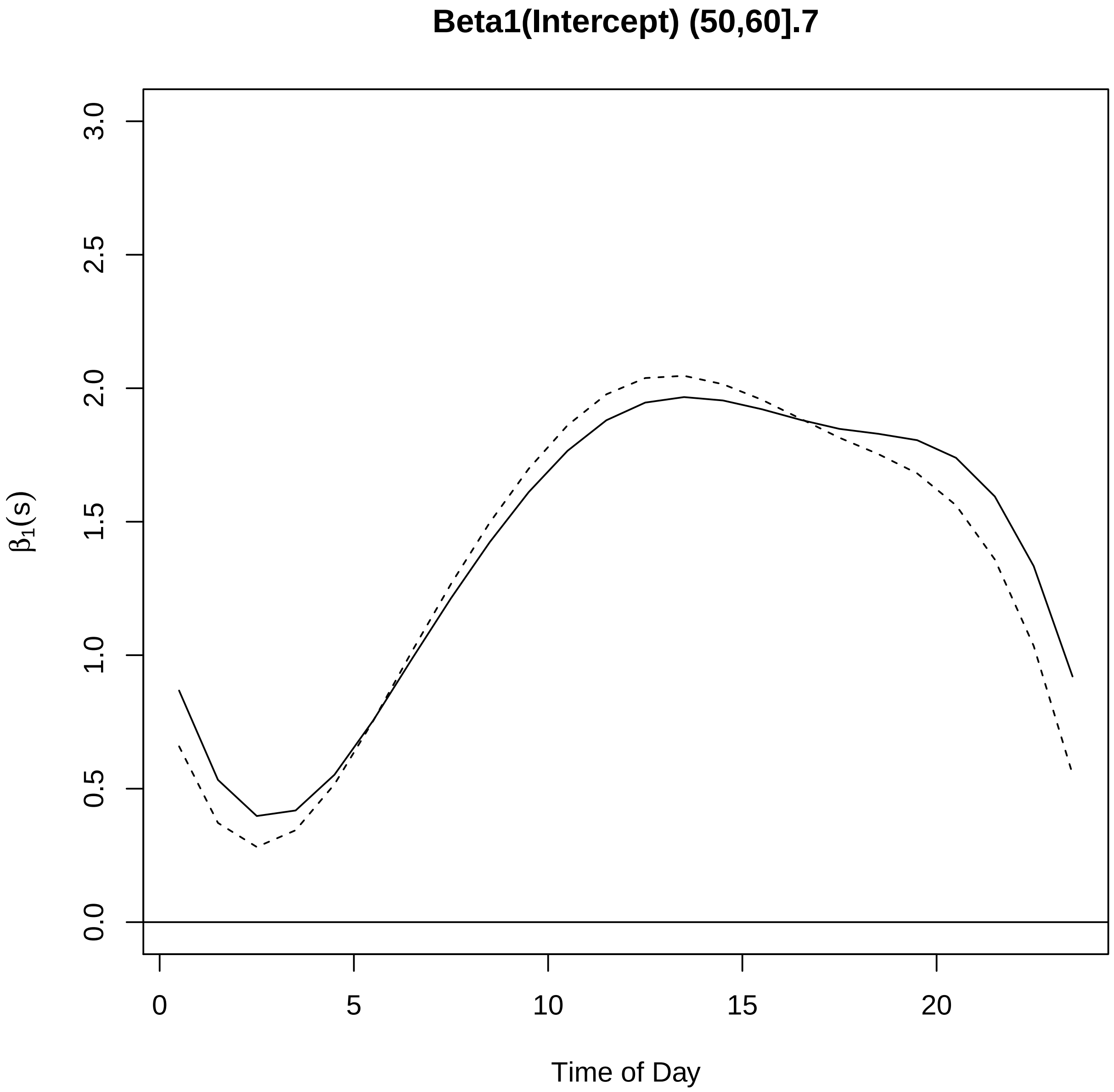

Figure S36: Predicted functional trajectories $(\hat{\beta}_1(s) + \hat{\beta}_{1i}(s))$ of diurnal MIMS for males from the MuFuMES method in the NHANES application. The other continuous covariates were held at their average values. The solid line corresponds to the fixed effect prediction $\hat{\beta}_1(s)$ and the dashed line corresponds to the mixed effects prediction $\hat{\beta}_1(s) + \hat{\beta}_{1i}(s)$. The age-by-race groups are indicated in the header.

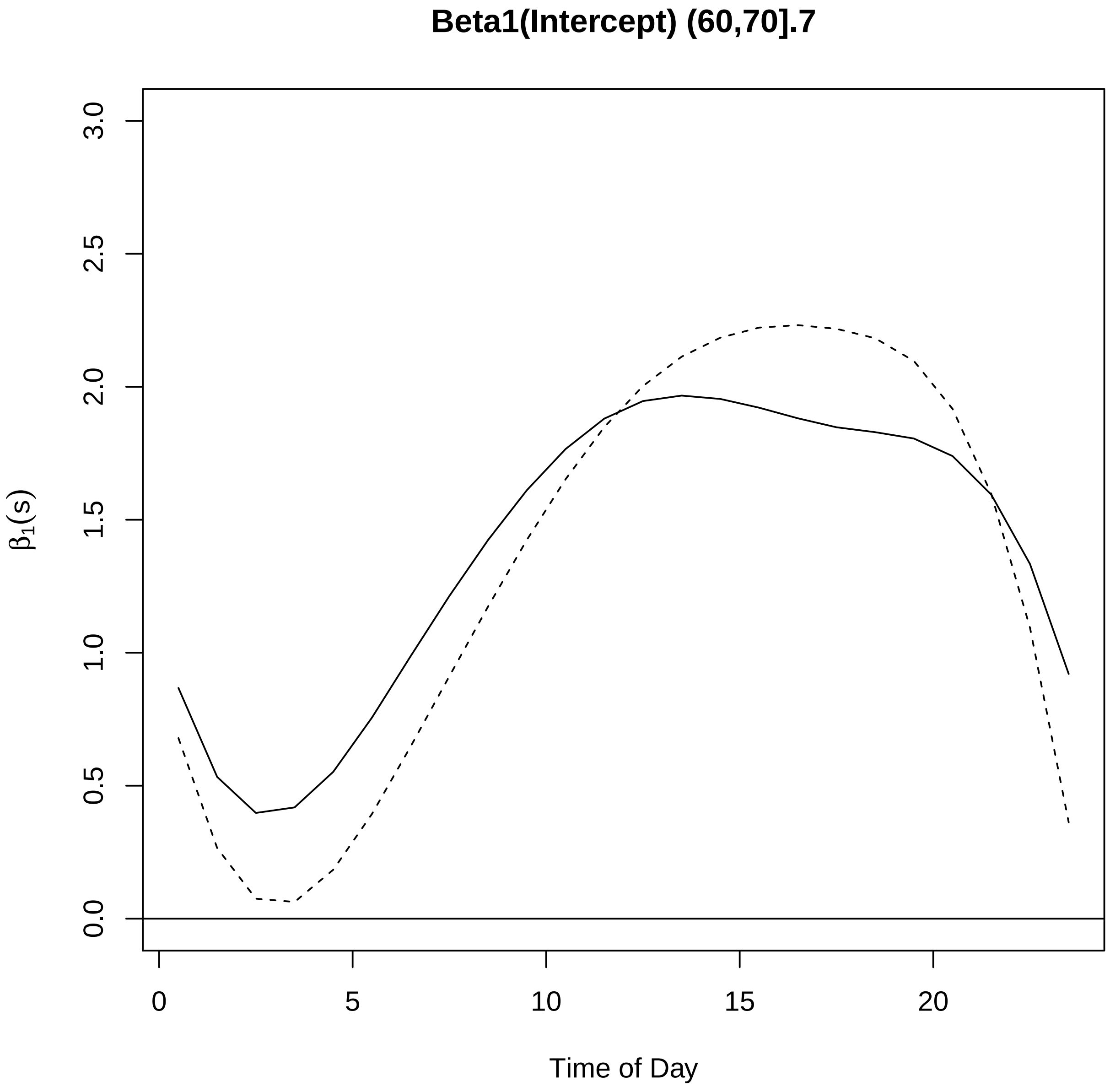

Figure S37: Predicted functional trajectories $(\hat{\beta}_1(s) + \hat{\beta}_{1i}(s))$ of diurnal MIMS for males from the MuFuMES method in the NHANES application. The other continuous covariates were held at their average values. The solid line corresponds to the fixed effect prediction $\hat{\beta}_1(s)$ and the dashed line corresponds to the mixed effects prediction $\hat{\beta}_1(s) + \hat{\beta}_{1i}(s)$. The age-by-race groups are indicated in the header.

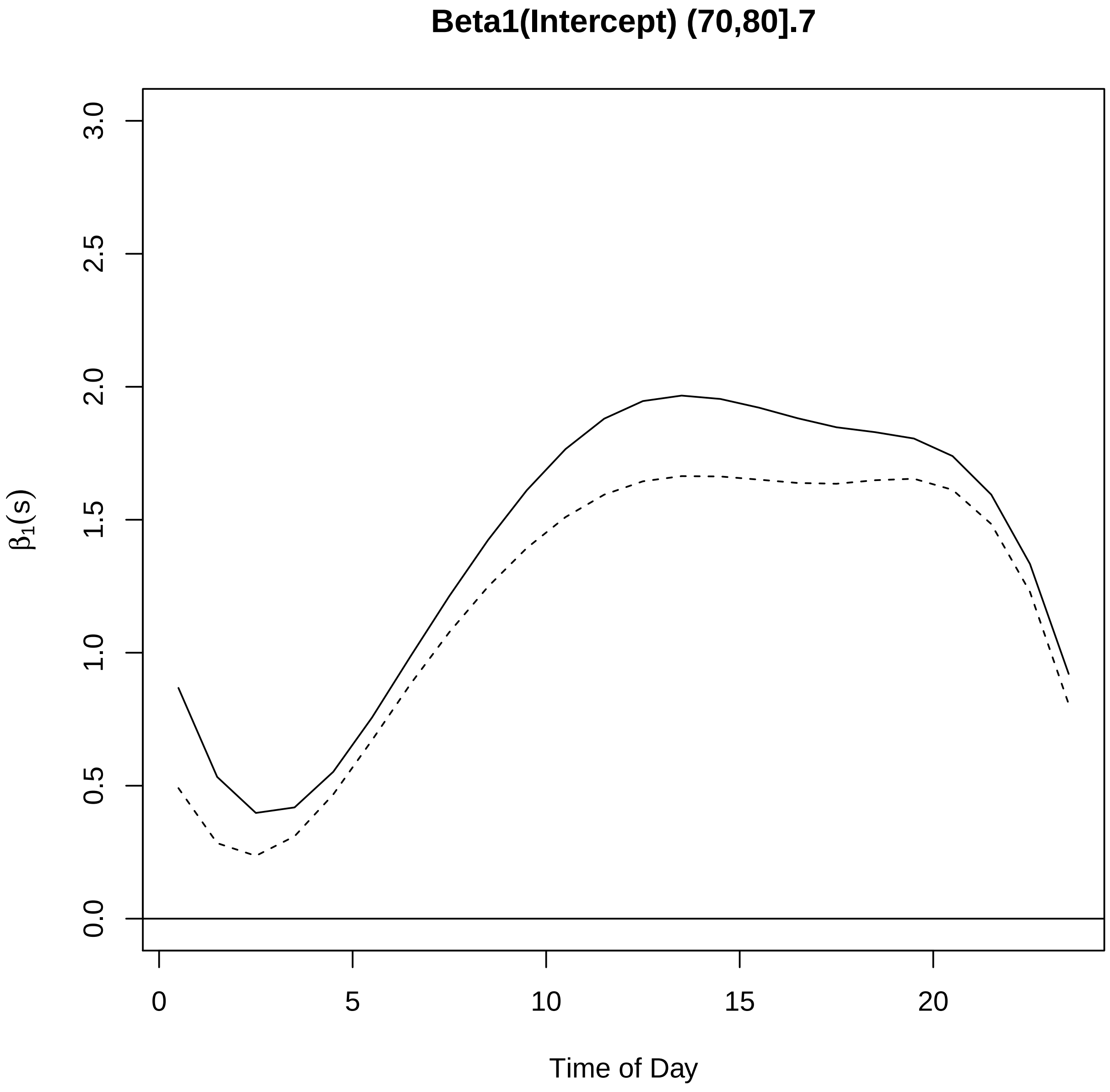

Figure S38: Predicted functional trajectories ($\hat{\beta}_1(s) + \hat{\beta}_{1i}(s)$) of diurnal MIMS for males from the MuFuMES method in the NHANES application. The other continuous covariates were held at their average values. The solid line corresponds to the fixed effect prediction $\hat{\beta}_1(s)$ and the dashed line corresponds to the mixed effects prediction $\hat{\beta}_1(s) + \hat{\beta}_{1i}(s)$. The age-by-race groups are indicated in the header.

References

Cui, E., A. Leroux, E. Smirnova, and C. M. Crainiceanu (2022). Fast univariate inference for longitudinal functional models. *Journal of Computational and Graphical*

Statistics 31(1), 219–230.

Scheipl, F., A.-M. Staicu, and S. Greven (2015). Functional additive mixed models. *Journal of Computational and Graphical Statistics 24*(2), 477–501.
