# Supplementary material for: Variable Selection for Fixed and Random Effects in Multilevel Functional Mixed Effects Models
Source: Stat Med. 2026 Aug 9;45(18-19):e70694. doi: 10.1002/sim.70694 (PMC13454179; doi:10.1002/sim.70694)
Supplement: Supplementary file 1 [file SIM-45-0-s001.zip › MuFuMES_Software.zip!/MuFuMES.html]

Multilevel Functional Mixed Effects Selection (MuFuMES)


# Multilevel Functional Mixed Effects Selection (MuFuMES)

#### Rahul Ghosal, Marcos Matabuena and Enakshi Saha

#### 10/23/2025

- 1 Introduction
- 2 Example: Multilevel Functional Mixed Effects Model

# 1 Introduction

This document presents an illustration of the Multilevel Functional Mixed Effects Selection (MuFuMES) method developed in Ghosal et al., 2025.

# 2 Example: Multilevel Functional Mixed Effects Model

First we generate data from a multilevel functional mixed effects model (Scenario B in paper).

```
###########Generating data##############################
library(Rcpp)
library(fda)
library(grpreg)
id=1 
set.seed(id)
# Generating data: Scenario B of the paper#
n<-I <- 50 #number of clusters
J <- 10 #number of observations per subject#
L <- 10 #dimension of the functional domain#
SNR_B <- 0.5 ## relative importance of random effects ##             
SNR_sigma <- 4 ## signal-to-noise ratio ##

p<- 10 #number of scalar predictors for X fixed effects,intercept separate#

q<-7 #number of random effect predictors Z except intercept#

S <- seq(0, 1, length = L) #functional domain

beta_true <- matrix(0, (p+1), L) #including intercept#


beta_true[1,] = 8*sin(2*S*pi) #intercept
beta_true[2,] = 2*dnorm(S, .6, .15) 
beta_true[3,] = 2.5*dnorm(S, .4, .15)
beta_true[4,] = 3*cos(2*pi*S)  
beta_true[5,] = 5*sin(2*pi*S)+5*cos(2*pi*S)   

##defining random effects, basis defined first##
psi_true <- matrix(NA, 4, L)
psi_true[1,] <- sin(2*S*pi)
psi_true[2,] <- cos(2*S*pi)
psi_true[3,] <- sin(1*S*pi)
psi_true[4,] <- cos(1*S*pi)

###this iteration of simulation starts##

## number of visits for each subject # could be set to random also
J_subj <- rep(J,I) 


## generate fixed effects design matrix #
nd <- sum(J_subj) #total subject days
X_des = cbind(1, matrix(rnorm(nd*p, 0, 1*2),nd,p)) #X matrix nd*(p+1)#
X_des_2 = cbind(1, matrix(rnorm(nd*q, 0, 1*2),nd,q)) #will be used for defining Zijk: random effect design matrices#

fixef <- X_des %*% beta_true #fixed effects

# generating random effects #
# random functional intercept#
subj <- as.factor(rep(1:I, J_subj)) 
Z_des <- model.matrix( ~ 0 + subj) #random subject-specific design matrix
library(mvtnorm)
c_true <- rmvnorm(I, mean = rep(0, 2), sigma = diag(c(3, 1.5)))
b_true <- c_true %*% psi_true[1:2,]
ranef = Z_des %*% b_true

# random functional slope (for fourth covariate)#
Z_des_2<-X_des_2[,4]*Z_des 
c_true_2 <- rmvnorm(I, mean = rep(0, 4), sigma = diag(c(1.5, 0.75,0.5,0.25))) 
b_true_2 <- c_true_2 %*% psi_true 
ranef2 = Z_des_2 %*% b_true_2

ranef<-ranef+ranef2
ranef <- sd(fixef)/sd(ranef)/SNR_B*ranef ## adjust for relative importance of random effects

# Generating Subject-Visit Level Random Effects v_ij(s)
SNR_V <- 2.0 
c_true_visit <- rmvnorm(nd, mean = rep(0, 2), sigma = diag(c(0.8, 0.4)))
ranef_visit <- c_true_visit %*% psi_true[3:4,]
# Scaling the new visit-level random effect relative to the fixed effect
ranef_visit <- sd(fixef)/sd(ranef_visit)/SNR_V*ranef_visit

eta_true <- fixef + ranef + ranef_visit

## generate multilevel functional data
Y_obs <- matrix(NA, nd, L) #(nd*L matrix format)
sd_signal <- sd(eta_true)

for(i in 1:nd){
  for(j in 1:L){
    Y_obs[i, j] <- rnorm(1, mean = eta_true[i, j], sd = sd_signal/SNR_sigma)
  }
}

X_matrix<-X_des   #fixed effect covariates
Z_matrix<-X_des_2 #random effect covariates
p<-ncol(X_matrix) #number of fixed effects (including intercept)
q<-ncol(Z_matrix) #number of random effects (including intercept)
```

Next we get the basis matrices corresponding to the fixed effect and random effect design matrices as described in equation (3) in the paper. We source the file “SourceMuFuMES.R” which contains all the required R functions for the MuFuMES method.

```
source("SourceMuFuMES.R") # source the main functions for MuFuMES#
#library(devtools)
#find_rtools()
nbasis=10 #number of B-spline basis functions for fixed and random effects
# get_basis_Matrices.MuFuMES: function for getting #
# the fixed and random effect design matrices #
out<-get_basis_Matrices.MuFuMES(Y_obs=Y_obs,X_matrix=X_matrix,Z_matrix=Z_matrix,subj=subj,nbasis=10)
Ylist=out$Ylist
Xmatlist=out$Xmatlist
Zmatlist=out$Zmatlist
Wmatlist=out$Wmatlist
bbasismat=out$bbasismat
```

Next we fit the MuFuMES method to select the important fixed and random functional effects.

```
#
sourceCpp('test.cpp') #source helper functions in cpp for MuFuMES#
lambda0grid<-c(20) #set to seq(50,5,by=-5) for larger grid-search in the full simulation##
lambda0_prime_grid<-c(20) ##set to seq(50,5,by=-5) for larger grid-search in the full simulation##
result<-MuFuMES.select.cv(Ylist=Ylist,Xmatlist=Xmatlist,Zmatlist=Zmatlist,Wmatlist=Wmatlist,lambda0grid=lambda0grid,lambda0_prime_grid=lambda0_prime_grid,nbasis=nbasis)
```

```
## [1] 1
##      difference1 difference2
## [1,]           1   0.9999828
## [1] 2
##      difference1 difference2
## [1,]  0.07062327    11.26279
## [1] 3
##      difference1 difference2
## [1,]  0.01168901    0.999934
## [1] 4
##       difference1 difference2
## [1,] 0.0007741327   0.9903531
## [1] 5
##       difference1  difference2
## [1,] 0.0002631979 8.831911e-05
## [1] 6
##       difference1 difference2
## [1,] 7.923677e-05 0.001257678
## [1] 7
##      difference1 difference2
## [1,] 2.62535e-05   0.9769709
## [1] 8
##       difference1 difference2
## [1,] 1.715652e-05 7.02306e-06
## [1] 1
```

```
result$varseleceted_fixed  #selected fixed effects, true ones are selected
```

```
## [1] 1 2 3 4 5
```

```
result$varseleceted_random #selected random effects, true ones are selected
```

```
## [1] 1 4
```

```
gamma<-result$gamma_vec #estimated fixed effect basis coefficients
L_p_vec<-result$L_p_vec #variance covariance parameters
#bvec<- result$bvec     #MAP estimates of b
```

We observe that the proposed MuFuMES method captures the true fixed and random functional effects accurately. Next we obtain estimates of the fixed functional effects from the estimated coefficients and display them along with the true curves.

```
#beta_func_est: function for getting functional fixed effect estimates from
# the MAP estimates of the basis coefficients and basis matrices #
beta_func_est<-get_func_fixed.est(bbasismat=bbasismat,gamma=gamma,p=p)
#list of estimates beta_k(s)#

#plotting slope parameters of interest: selected fixed effects#
par(mar = c(4, 4, 2, 2)) # Set the margin on all sides to 2
par(mfrow=c(2,2))
plot(S,beta_true[2,],type="l",ylab=expression(paste(beta[2](s))))
lines(S,beta_func_est[[2]],col=4,lty=2) 

plot(S,beta_true[3,],type="l",ylab=expression(paste(beta[3](s))))
lines(S,beta_func_est[[3]],col=4,lty=2) 

plot(S,beta_true[4,],type="l",ylab=expression(paste(beta[4](s))))
lines(S,beta_func_est[[4]],col=4,lty=2) 

plot(S,beta_true[5,],type="l",ylab=expression(paste(beta[5](s))))
lines(S,beta_func_est[[5]],col=4,lty=2)
```

The functional fixed effects are closely captured by the proposed MuFuMES method.
